# Voxel-wise functional-to-gray matter volume ratios reveal distinct brain patterns in cirrhosis stratified by hepatic encephalopathy history and clinical status

**DOI:** 10.3389/fneur.2026.1969935

**Published:** 2026-10-19

**Authors:** Zhen Yu, Minghui Mao, Qianghong Xue, Chengkun Hong, Hao Huang, Aling Shen, Liyuan Fu

**Affiliations:** 1 Fuzong Teaching Hospital of Fujian University of Traditional Chinese Medicine (900th Hospital), Fuzhou, China; 2 College of Integrative Medicine, Fujian University of Traditional Chinese Medicine, Fuzhou, China; 3 The Third People’s Hospital Affiliated to Fujian University of Traditional Chinese Medicine, Fuzhou, China

**Keywords:** amplitude of low-frequency fluctuation, cirrhosis, gray matter volume, hepatic encephalopathy, neuroimaging, regional homogeneity, resting-state fMRI

## Abstract

**Background:**

Hepatic encephalopathy (HE) is associated with distributed structural and functional brain abnormalities, but how local resting-state functional signals vary relative to gray matter volume (GMV) remains uncertain.

**Methods:**

We retrospectively analyzed previously acquired magnetic resonance imaging (MRI) and clinical data from 97 participants after motion exclusion: 38 healthy controls (HCs), 29 patients with cirrhosis who had no history of overt HE and were classified as West Haven grade 0 at enrollment (NHE), and 30 patients in an operationally defined HE group. Voxel-wise regional homogeneity-to-GMV (ReHo-to-GMV) and amplitude of low-frequency fluctuation-to-GMV (ALFF-to-GMV) ratios were assessed in separate 3-group models adjusted for age, sex, education, and mean framewise displacement. Gaussian random-field cluster inference was used for the primary analysis, with 10,000-permutation threshold-free cluster enhancement (TFCE) as a non-parametric sensitivity analysis.

**Results:**

Corrected abnormalities were concentrated in HE versus HCs, whereas neither metric showed a corrected HE-versus-NHE difference. ReHo-to-GMV and ALFF-to-GMV each yielded 13 corrected clusters across the test-specific omnibus and directional results. Permutation and local-GMV-adjusted sensitivity analyses supported the principal HE-versus-HC effects. Cross-metric overlap was limited to 31 HCs > HE voxels (Dice = 0.0541), and none of the 48 exploratory region-of-interest clinical associations survived false-discovery-rate correction.

**Conclusion:**

These ratios identify partly distinct group-level neuroimaging patterns of local functional signals relative to GMV in cirrhosis, but they should be interpreted as operational imaging measures rather than direct physiological coupling or validated biomarkers.

## 1 Introduction

Hepatic encephalopathy (HE) is a brain dysfunction associated with liver insufficiency and/or portosystemic shunting, ranging from subtle cognitive impairment to overt neuropsychiatric manifestations (1, 2). Covert HE can affect attention, working memory, psychomotor speed, inhibition, and executive function, while the boundary between minimal and grade 1 HE remains difficult to reproduce consistently in clinical practice (3). Structural MRI studies have reported gray matter volume (GMV) abnormalities in thalamic, insular, basal ganglia, cingulate, frontal, parietal, and cerebellar regions, but the distribution and direction of these changes vary across studies (4–6).

Resting-state functional MRI provides indirect measures of spontaneous blood-oxygen-level-dependent activity. Regional homogeneity (ReHo) describes local temporal concordance, whereas the amplitude of low-frequency fluctuation (ALFF) reflects fluctuation magnitude within a predefined low-frequency band (7). Previous studies in cirrhosis and HE have identified distributed ReHo- and ALFF-related abnormalities, but findings vary with phenotype, metric, preprocessing strategy, and statistical threshold (8–10). Examining GMV, ReHo, and ALFF separately, however, does not describe how a local functional signal varies relative to its structural background.

Voxel-wise functional-to-GMV ratios provide an operational way to summarize local functional signal relative to GMV and have been applied in other neurological settings (11, 12). Such ratios are mathematical constructs rather than direct physiological measures: a ratio may change because of its functional numerator, its GMV denominator, or both, and ratio-based analyses can be vulnerable to denominator variability and interpretive bias (13, 14). These limitations make explicit component and sensitivity analyses important when ratio maps are used to characterize group-level brain patterns.

We therefore applied two voxel-wise functional-to-GMV ratios, ReHo-to-GMV and ALFF-to-GMV, to characterize group-level brain patterns across healthy controls (HCs), an operational NHE group defined by no previous overt HE (OHE) and West Haven grade 0 at enrollment, and an operationally defined HE group. The principal analysis evaluated ReHo-to-GMV across the three groups, while ALFF-to-GMV was examined as a secondary cross-metric analysis. Particular attention was given to whether either metric showed corrected separation between the two operational patient groups. We additionally assessed non-parametric support, component-level spatial context, total intracranial volume (TIV) and motion sensitivity, final-sample mask stability, and cross-metric concordance. Exploratory clinical analyses were restricted to imaging-defined regions and were interpreted without diagnostic, staging, predictive, biomarker, or causal claims.

## 2 Materials and methods

### 2.1 Ethics and participants

The institutional biomedical ethics committee approved the retrospective use and analysis of previously acquired imaging and clinical data (IRB no. 2025–120). All participants had provided written informed consent for the use of their clinical and imaging data. Data were acquired at a single tertiary hospital between January 2023 and December 2025. The initial sample comprised 42 HCs, 37 participants in the NHE group, and 39 participants in the HE group. Cirrhosis was diagnosed according to established clinical guidelines (15).

Patients were classified according to previous OHE and West Haven grade at enrollment (16). NHE required no previous OHE and West Haven grade 0 at enrollment. The HE group included participants with previous OHE and/or current West Haven grade 1–2 HE at enrollment. Participants with previous OHE who had recovered to West Haven grade 0 at enrollment remained classified in the HE group. Grade 3–4 HE was excluded. For descriptive phenotype characterization, the HE group was further classified according to current West Haven grade and previous OHE history. The Psychometric Hepatic Encephalopathy Score (PHES) was analyzed as a cognitive measure and did not redefine the primary imaging groups. Thus, NHE was an operational classification based on OHE history and West Haven grade and did not exclude minimal HE solely on the basis of PHES. The main eligibility criteria were age 18–75 years, right-handedness, ability to complete MRI and neuropsychological testing, and absence of major neurological, psychiatric, structural brain, malignant, severe infectious, cardiopulmonary, or rapidly progressive liver disease. Recent alcohol use, substance dependence, relevant psychoactive medication, MRI contraindication, incomplete examination, and inadequate image quality were also exclusion criteria.

Head-motion quality control used prespecified thresholds of maximum translation > 3 mm, maximum rotation > 3°, or mean Jenkinson framewise displacement (FD) > 0.20 mm. Twenty-one participants were excluded (4 HCs, 8 participants in the NHE group, and 9 participants in the HE group), leaving 97 participants for voxel-wise analysis (mean age, 55.8 ± 7.8 years; 40 women [41.2%]): 38 HCs, 29 participants in the NHE group, and 30 participants in the HE group (Figure 1). Group-level motion-exclusion counts and quality-control criteria are provided in Supplementary Table 1.

**Figure 1:**
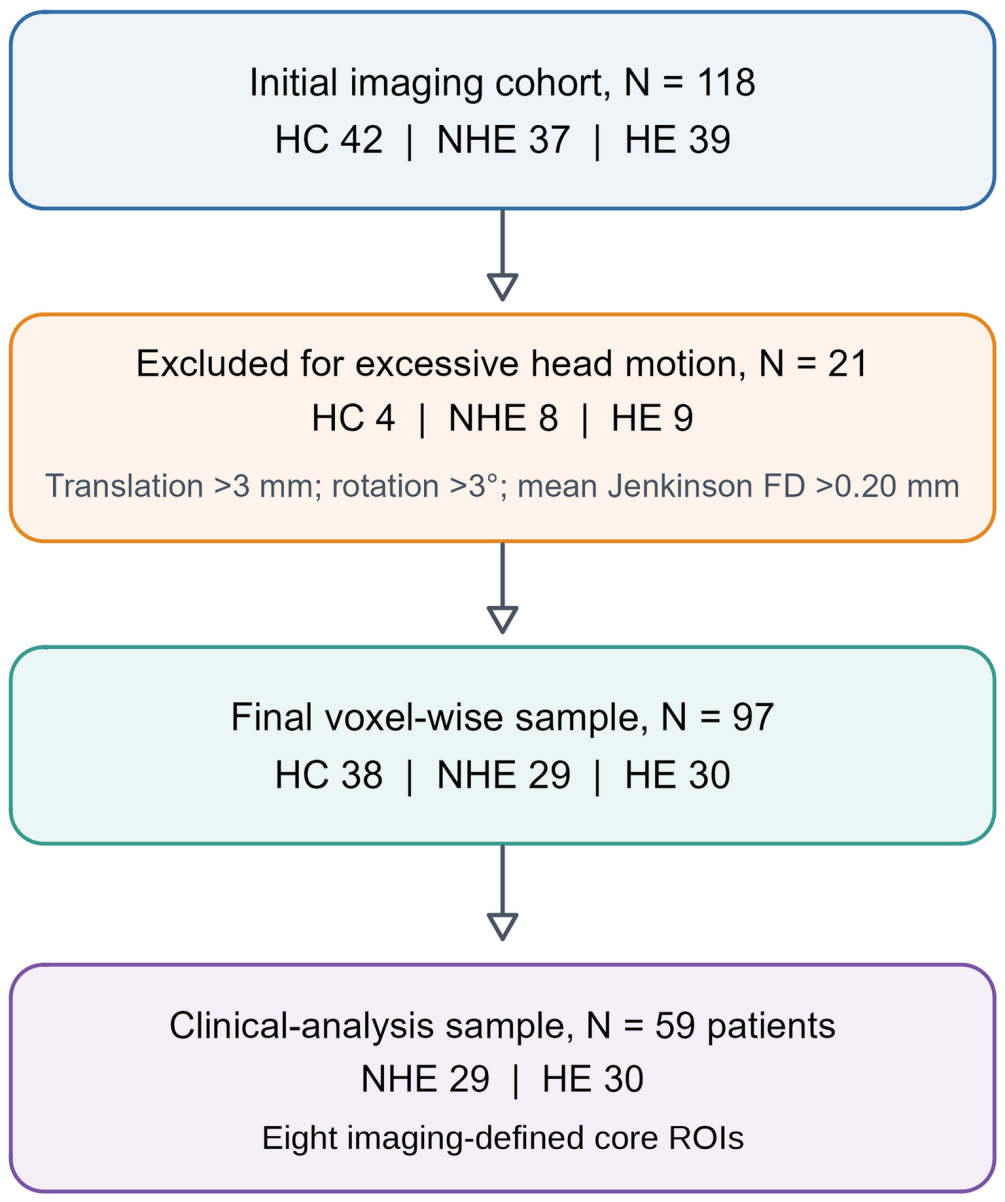
Participant flow and analysis samples. The initial cohort included 118 participants (42 healthy controls [HC], 37 patients in the operational NHE group, and 39 patients in the hepatic encephalopathy [HE] group). Twenty-one were excluded for translation > 3 mm, rotation > 3°, or mean Jenkinson framewise displacement > 0.20 mm, leaving 97 for voxel-wise analysis (38 HC, 29 NHE, and 30 HE). Exploratory clinical analyses included 59 patients and 8 imaging-defined core regions of interest. NHE required no previous overt hepatic encephalopathy (OHE) and West Haven grade 0 at enrollment; the HE group included previous OHE and/or current West Haven grade 1–2 HE. Among the 30 HE participants, 11 had previous OHE with current West Haven grade 0 and 19 had current grade 1–2 HE; 6 of the latter also had previous OHE. The Psychometric Hepatic Encephalopathy Score (PHES) was not used to redefine the primary imaging groups.

### 2.2 Clinical and cognitive assessments

All participants completed the 5-subtest PHES, Mini-Mental State Examination (MMSE), and Montreal Cognitive Assessment (MoCA) on the MRI day in a quiet, appropriately lit environment. PHES comprised Number Connection Tests A and B, Digit Symbol Test, Serial Dotting Test, and Line-Tracing Test and was scored using age- and education-adjusted Chinese norms (3, 17). Trained research personnel administered all tests using standardized procedures.

In patients, venous blood was collected within 72 h before MRI. Recorded liver-related variables included blood ammonia, albumin, total bilirubin, cholinesterase, creatinine, estimated glomerular filtration rate, sodium, coagulation measures, blood counts, Child-Pugh score/class, and Model for End-Stage Liver Disease (MELD) score. MELD was calculated with the conventional 3-variable formula; bilirubin and creatinine were converted to mg/dL, values below 1.0 were set to 1.0, creatinine was capped at 4.0 mg/dL, and the calculated value was retained without integer rounding (18). Complete MELD data were available for all 59 patients.

### 2.3 MRI acquisition

MRI was performed on a 3.0-T United Imaging uMR 770 system with a 32-channel head coil. Participants were instructed to keep their eyes closed, remain awake, relax, and minimize motion, with foam padding used for stabilization. Three-dimensional T1-weighted imaging used repetition time/echo time = 6.95/3.0 ms, flip angle = 9°, acquisition matrix = 256 × 256, reconstructed matrix = 512 × 512, 160 sagittal slices, field of view = 256 × 256 mm^2^, native voxel size = 1.0 × 1.0 × 1.0 mm^3^, and reconstructed voxel size = 0.5 × 0.5 × 1.0 mm^3^.

Resting-state echo-planar imaging comprised 240 volumes with repetition time/echo time = 2,000/30 ms, flip angle = 90°, matrix = 64 × 64, 33 axial slices, field of view = 240 × 240 mm^2^, voxel size = 3.75 × 3.75 × 4.00 mm^3^, slice thickness = 4.0 mm, no interslice gap, anterior-to-posterior phase encoding, and descending interleaved acquisition. The complete slice order is provided in the Supplementary material.

### 2.4 Image preprocessing

Resting-state data were preprocessed using DPARSFA in DPABI v6.0 (19). The first 10 volumes were discarded. Slice-timing correction and rigid-body realignment were followed by motion assessment using Jenkinson FD (20). T1-weighted images were coregistered to the mean functional image, segmented into gray matter, white matter, and cerebrospinal fluid with SPM New Segment (21), and normalized to Montreal Neurological Institute (MNI) space using DARTEL (22); functional images were resampled to 3-mm isotropic voxels.

Nuisance regression included the Friston-24 motion parameters (23), mean white-matter and cerebrospinal-fluid signals, and a linear trend. Global signal regression, scrubbing, and susceptibility-distortion correction were not performed. For ReHo, the residual time series were filtered at 0.01–0.08 Hz, ReHo was calculated using a 27-voxel neighborhood, standardized to zReHo, and smoothed with a 4-mm full-width at half-maximum (FWHM) kernel; this smoothed standardized ReHo output is denoted szReHo below. ALFF was calculated at 0.01–0.08 Hz after 4-mm smoothing and then standardized. Modulated normalized GMV maps were smoothed at 8 mm and resampled to the functional analysis grid. TIV was calculated as the sum of native-space gray matter, white matter, and cerebrospinal-fluid volumes.

### 2.5 Voxel-wise ratio calculation and common gray-matter mask

Within the strict common gray-matter mask, the functional measure at each voxel was divided by the GMV value at the same voxel. The operational ratios were defined in Equations (1) and (2):

ReHo−to−GMV(v)=szReHo(v)/rsmwc1(v) (1)

ALFF−to−GMV(v)=zALFF(v)/rsmwc1(v) (2)

Here, szReHo and zALFF denote the standardized functional measures, and rsmwc1 denotes the smoothed, modulated GMV map resampled to the functional grid. The ratios were treated as operational descriptions of local functional signal relative to GMV, not as direct physiological coupling or causal structure–function measures. A conceptual illustration of how different numerator-denominator configurations can produce similar functional-to-GMV ratio changes is provided in Supplementary Figure 4.

The common mask was constructed in 3 × 3 × 3 mm^3^ space by intersecting an SPM gray-matter probability map > 0.20, the all-participant mean GMV map > 0.10, and voxels with GMV > 0.05 in every participant. The resulting strict mask contained 42,885 voxels and was used both to calculate ratio maps and as the explicit mask in voxel-wise models (Figure 2). This procedure was intended to reduce exposure to very small denominators.

**Figure 2:**
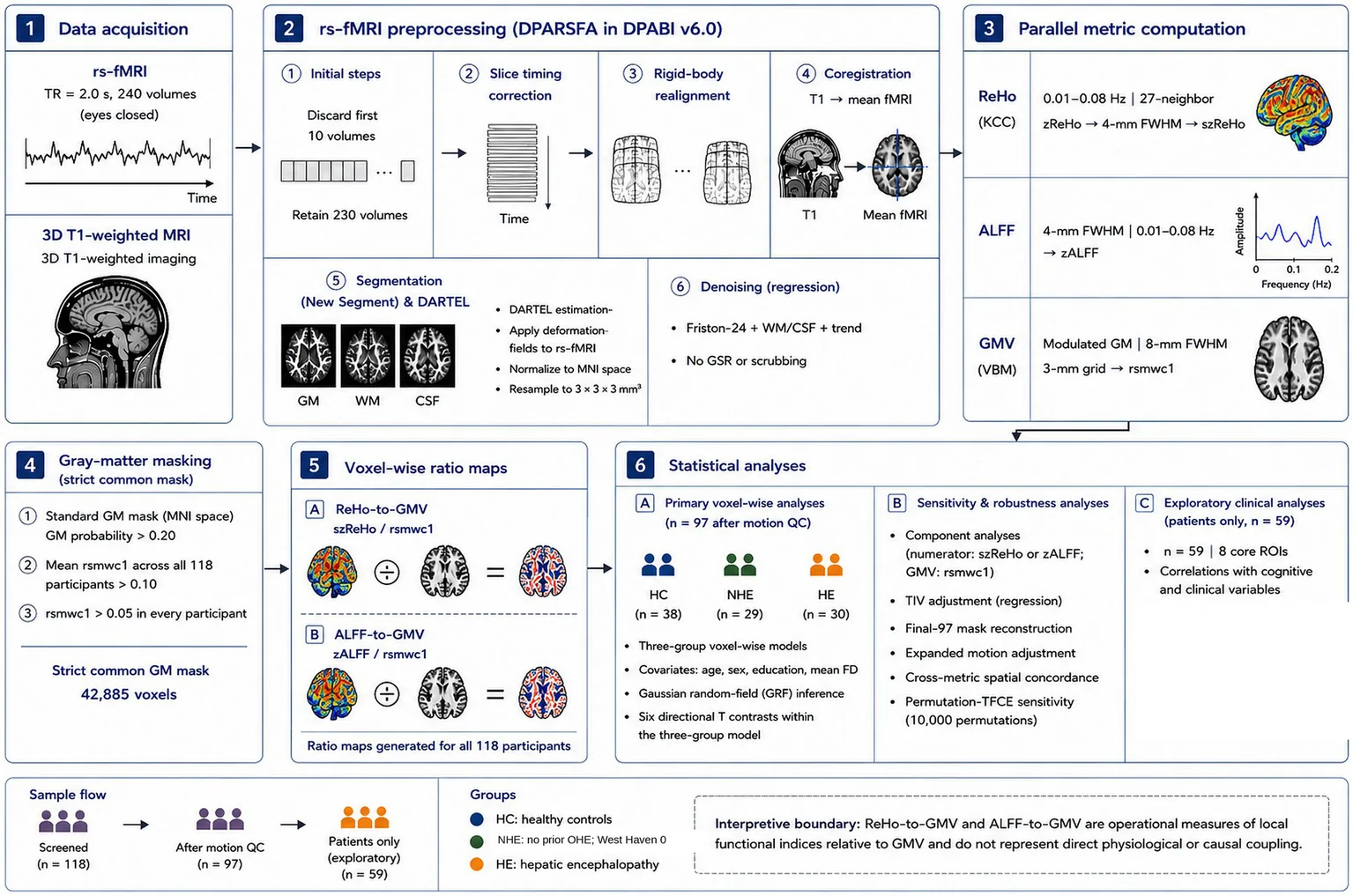
MRI preprocessing and voxel-wise ratio workflow. The first 10 of 240 resting-state functional MRI volumes were discarded. Preprocessing included slice timing, realignment, T1 coregistration and segmentation, DARTEL normalization, 3-mm resampling, and nuisance regression. ReHo was standardized and smoothed at 4 mm; ALFF was calculated at 0.01–0.08 Hz after 4-mm smoothing; modulated GMV was smoothed at 8 mm and resampled. The strict common gray-matter mask required SPM gray-matter probability > 0.20, mean GMV > 0.10, and GMV > 0.05 in every participant (42,885 voxels). ReHo-to-GMV = szReHo/rsmwc1 and ALFF-to-GMV = zALFF/rsmwc1. ALFF = amplitude of low-frequency fluctuation, CSF = cerebrospinal fluid, FD = framewise displacement, FWHM = full-width at half-maximum, GMV = gray matter volume, GSR = global signal regression, KCC = Kendall’s coefficient of concordance, MNI = Montreal Neurological Institute, ReHo = regional homogeneity, TIV = total intracranial volume, WM = white matter.

### 2.6 Primary voxel-wise statistical analysis

ReHo-to-GMV and ALFF-to-GMV were analyzed in separate three-group one-way voxel-wise models. Groups were specified as independent, and equal variances were not assumed. Age, sex, years of education, and mean FD were centered on the overall mean and entered as covariates with common slopes; group-by-covariate interactions were not modeled. The strict common gray-matter mask was specified explicitly. Threshold masking, implicit masking, global-mean calculation, global scaling, and global normalization were not applied.

For each metric, one omnibus F test and six directional pairwise contrasts were specified. Tests were organized into an omnibus family, an HE–NHE directional family, and an HC-related directional family. The voxel-defining threshold was *p* < 0.001 uncorrected, k = 0, with SPM 18-connectivity. Cluster-level family-wise error (FWE) thresholds were *p* < 0.050 for the omnibus test, *p* < 0.025 for each HE–NHE direction, and *p* < 0.0125 for each HC-related direction. Directional contrasts were evaluated independently and were not gated or inclusively masked by the omnibus result. ReHo-to-GMV constituted the principal ratio analysis, whereas ALFF-to-GMV was evaluated as a secondary cross-metric analysis. Because the two metrics were fitted in separate voxel-wise models and served different analytical roles, multiplicity correction was applied within metric-specific, analysis-defined hypothesis families rather than jointly across both metrics. No single experiment-wide alpha allocation was applied across both ratio metrics and all secondary or exploratory analyses.

### 2.7 Sensitivity, component, and spatial-concordance analyses

Several within-sample analyses assessed robustness and interpretation. A TIV-adjusted model added TIV to the primary covariates. For each primary cluster, spatial consistency with the same-direction TIV-adjusted corrected map was summarized by overlap coverage and nearest-peak distance. Strong spatial consistency required overlap, coverage ≥ 0.50, and nearest-peak distance ≤ 12 mm; clusters with overlap not meeting both criteria were classified as partial, while corrected sensitivity voxels without overlap were described as spatially shifted. These categories were descriptive and did not estimate reproducibility.

Eight core regions of interest (ROIs) were selected before clinical association testing using an imaging-only rule: eligible clusters arose from directional contrasts, retained TIV-adjusted overlap, and intersected the corresponding corrected omnibus map. Clinical variables and association results were not used in ROI selection. *Post hoc* whole-brain component models separately examined szReHo, zALFF, and GMV using the same group design and inferential thresholds. Corrected ratio maps were compared with same-direction functional-numerator maps and opposite-direction GMV maps to provide threshold-dependent spatial context; these comparisons did not quantify component contributions.

Two additional sensitivity analyses reconstructed the strict common mask using only the final 97 participants and expanded the motion model by adding centered maximum translation and maximum rotation. Non-parametric sensitivity analysis used Permutation Analysis of Linear Models (PALM) with 10,000 permutations, Freedman-Lane nuisance handling, 2-tailed threshold-free cluster enhancement (TFCE), and within-contrast FWE *p* < 0.050. Joint correction across the 3 pairwise contrasts was an additional conservative analysis. Because all sensitivity analyses used the same cohort, they were interpreted as internal robustness checks rather than independent validation.

To address potential denominator-driven effects, additional voxel-wise local-GMV-adjusted functional models were fitted separately for szReHo and zALFF. Group was the factor of interest, with age, sex, education, mean FD, and voxel-wise local GMV as covariates. Local GMV was centered across the 97 participants at each voxel and entered as a voxel-wise explanatory variable with a common slope across groups. PALM used 500 permutations with tail acceleration, Freedman-Lane nuisance handling, 2-tailed TFCE, and joint correction across the 3 pairwise contrasts. The strict mask contained 42,885 voxels; all were retained for ReHo, whereas 365 voxels with ALFF values identically equal to 0 across all participants were excluded, yielding an effective ALFF mask of 42,520 voxels. Same-direction spatial correspondence with the original ratio maps was summarized using voxel retention, Dice coefficients, and Jaccard indices. Additional methodological details and effective-mask quality control are provided in Supplementary material (Section S3) and Supplementary Table 22.

To further address clinical heterogeneity within the operational HE group, an exploratory within-HE phenotype sensitivity analysis compared participants with current West Haven grade 1–2 HE (*n* = 19) with those who had previous OHE but had recovered to West Haven grade 0 at enrollment (*n* = 11). ReHo-to-GMV and ALFF-to-GMV were analyzed separately using PALM with age, sex, education, and mean FD as covariates. Inference used 10,000 permutations, Freedman-Lane nuisance handling, 2-tailed TFCE, and FWE correction at *p* < 0.050. The original strict gray-matter mask was retained; the effective masks comprised 42,885 voxels for ReHo-to-GMV and 42,520 voxels for ALFF-to-GMV. This analysis was considered exploratory and was not used to redefine the primary imaging groups. Additional subgroup details are provided in Supplementary material (Section S5) and Supplementary Table 26.

### 2.8 Exploratory clinical analyses

Exploratory clinical analyses included 59 patients (29 NHE and 30 HE) and 8 imaging-defined core ROIs. For covariate-adjusted partial Spearman correlations, ranked imaging and clinical variables were separately residualized for group, sex, age, education, and mean FD; Pearson correlations between residuals provided partial rank correlations. Stratified 5,000-resample bootstrap confidence intervals quantified uncertainty. PHES, MMSE/MoCA, and blood ammonia/Child-Pugh/MELD were treated as separate analysis families with Benjamini–Hochberg false-discovery-rate (FDR) control (24). A TIV-adjusted sensitivity model added ranked TIV.

### 2.9 Software and descriptive statistics

Voxel-wise models and random-field-theory inference were implemented in MATLAB R2023b and SPM25; clinical analyses were performed in R 4.6.1. Demographic comparisons used one-way analysis of variance, Pearson χ^2^ tests, or Kruskal–Wallis tests as appropriate, with Tukey or Holm-adjusted *post hoc* comparisons. Patient laboratory variables were compared with independent-samples or Welch t tests, Mann–Whitney U tests, or Fisher’s exact tests as appropriate, with FDR control across the patient-group clinical/laboratory family. Additional methodological details and complete sensitivity outputs are provided in the Supplementary material.

## 3 Results

### 3.1 ReHo-to-GMV findings

ReHo-to-GMV produced 6 corrected omnibus clusters and 7 corrected directional clusters: 1 HE > HC cluster in the right thalamus, 5 HC > HE clusters involving anterior cingulate/medial frontal, insular-putaminal-opercular, superior temporal, and perirolandic-parietal regions, and 1 NHE > HC precentral cluster. Neither HE > NHE nor NHE > HE yielded corrected ReHo-to-GMV clusters, and no HC > NHE cluster survived correction (Figure 3; Table 1). The NHE > HC cluster did not overlap the omnibus map and was retained but interpreted cautiously because directional contrasts were not gated by the omnibus test.

**Figure 3:**
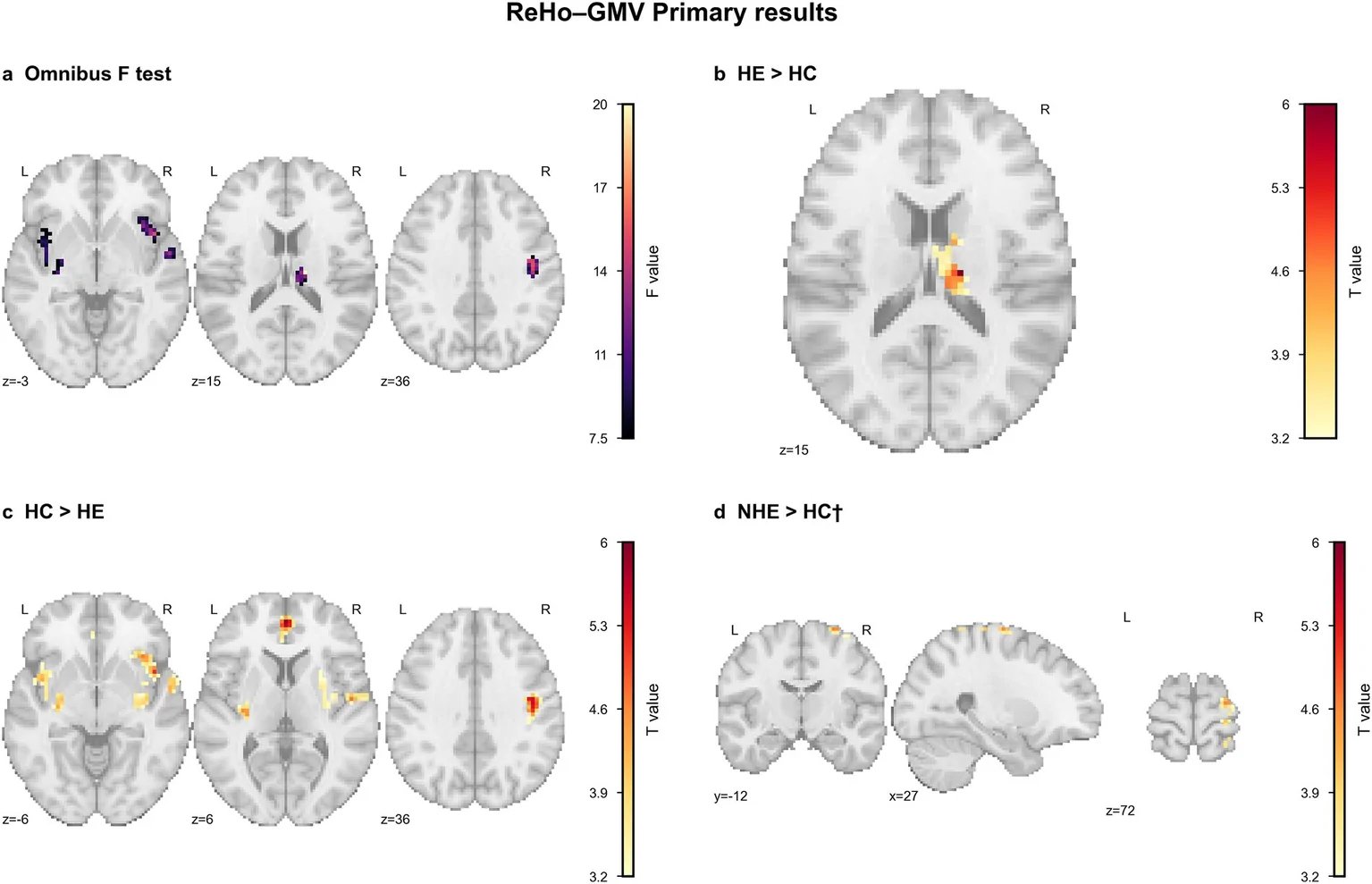
Primary ReHo-to-GMV findings. **(a)** Corrected omnibus F-test clusters. **(b)** HE > HC. **(c)** HC > HE. **(d)** NHE > HC cluster centered at Montreal Neurological Institute coordinates (27, −12, 72). Maps use neurological convention. The voxel-defining threshold was *p* < 0.001 uncorrected; cluster-level family-wise error thresholds were *p* < 0.050 for the omnibus test and *p* < 0.0125 for HC-related directional contrasts. HE > NHE and NHE > HE were null. The NHE > HC cluster did not overlap the omnibus map. HC = healthy controls, HE = hepatic encephalopathy, NHE = no previous overt hepatic encephalopathy and West Haven grade 0 at enrollment, ReHo = regional homogeneity.

**Table 1:** Formally significant primary ReHo-to-GMV clusters.

Contrast	Anatomical region	Cluster size, voxels	Cluster FWE P	Peak statistic	Peak MNI coordinates	TIV-adjusted spatial consistency
Omnibus F test	Right thalamus	31	0.046	19.86	(15, −18, 15)	Spatially shifted
Omnibus F test	Left anterior cingulate gyrus	40	0.016	18.29	(0, 51, 6)	Spatially shifted
Omnibus F test	Right perirolandic region involving the postcentral and precentral gyri	35	0.028	15.08	(45, −12, 36)	Spatially shifted
Omnibus F test	Right anterior insula	77	< 0.001	14.99	(45, 12, −3)	Strong spatial consistency
Omnibus F test	Right superior temporal and opercular region	38	0.020	13.04	(57, −3, −3)	Strong spatial consistency
Omnibus F test	Left anterior insula	75	< 0.001	13.00	(−27, −9, −6)	Partial spatial consistency
HE > HC	Right thalamus	65	0.006	5.99	(15, −18, 15)	Strong spatial consistency
HC > HE	Left anterior cingulate gyrus	99	< 0.001	5.91	(0, 51, 6)	Strong spatial consistency
HC > HE	Right perirolandic and supramarginal region	92	< 0.001	5.47	(45, −12, 36)	Strong spatial consistency
HC > HE	Right insular, putaminal, and opercular region	238	< 0.001	5.13	(45, 12, −6)	Strong spatial consistency
HC > HE	Right superior temporal and opercular region	100	< 0.001	5.03	(57, −3, −3)	Strong spatial consistency
HC > HE	Left anterior insula	118	< 0.001	4.62	(−30, −21, 3)	Strong spatial consistency
NHE > HC†	Right precentral gyrus	80	0.002	4.59	(27, −12, 72)	Strong spatial consistency

The voxel-defining threshold was *p* < 0.001 uncorrected, k = 0, with 18-connectivity. Cluster-level FWE thresholds were *p* < 0.050 for the omnibus test, *p* < 0.025 for HE–NHE directional contrasts, and *p* < 0.0125 for HC-related directional contrasts. The peak statistic is F for omnibus tests and t for directional contrasts. Coordinates are in Montreal Neurological Institute space. †This directional cluster did not overlap the corrected omnibus map. FWE = family-wise error, HC = healthy controls, HE = hepatic encephalopathy, NHE = no previous overt hepatic encephalopathy and West Haven grade 0 at enrollment, TIV = total intracranial volume.

### 3.2 Demographic and clinical characteristics

Baseline characteristics are summarized in Table 2. Within the HE group (*n* = 30), 11 participants (36.7%) were West Haven grade 0 at enrollment, 12 (40.0%) were grade 1, and 7 (23.3%) were grade 2. Seventeen participants (56.7%) had previous OHE. All 11 participants with West Haven grade 0 had previous OHE, and 6 of the 19 participants with current West Haven grade 1–2 HE also had previous OHE. Thus, 11 participants had recovered previous OHE with current grade 0, 6 had current grade 1–2 HE with previous OHE, and 13 had current grade 1–2 HE without previous OHE. Among the 29 NHE participants, 3 (10.3%) had PHES scores ≤ − 4 and 26 (89.7%) had scores above this threshold. TIV differed overall (*p* = 0.049; HE vs. HC adjusted *p* = 0.039), and mean FD differed among groups (*p* = 0.002) and was included as a model covariate. PHES, MMSE, and MoCA differed among groups (all *p* < 0.001). Blood ammonia and multiple liver-function variables differed between NHE and HE after FDR correction; complete patient-group clinical and laboratory results are provided in Supplementary Table 2.

**Table 2:** Demographic, motion, cognitive, and selected clinical/laboratory characteristics.

Variable	HC (*n* = 38)	NHE (*n* = 29)	HE (*n* = 30)	*p* value	Adjusted P/BH–FDR q
Age, years	54.8 ± 8.2	56.1 ± 7.7	56.8 ± 7.6	0.566	
Female, *n* (%)	16 (42.1%)	15 (51.7%)	9 (30.0%)	0.236	
Education, years	8.4 ± 2.9	7.9 ± 2.9	8.4 ± 2.5	0.750	
TIV, mL	1412.1 ± 125.8	1437.8 ± 117.1	1487.3 ± 128.4	0.049	HE vs. HC: adjusted *p* = 0.039
Mean Jenkinson framewise displacement, mm	0.076 [0.053, 0.111]	0.106 [0.074, 0.145]	0.114 [0.087, 0.153]	0.002	HC vs. NHE: 0.020; HC vs. HE: 0.003
PHES	0.0 [0.0, 0.0]	−1.0 [−2.0, 0.0]	−5.0 [−6.0, −4.0]	< 0.001	All pairwise adjusted *p* < 0.001
MMSE	30.0 [29.2, 30.0]	29.0 [28.0, 30.0]	27.0 [26.0, 28.8]	< 0.001	HC vs. NHE: 0.001; HC vs. HE: < 0.001; NHE vs. HE: 0.009
MoCA	30.0 [29.0, 30.0]	28.0 [27.0, 29.0]	25.5 [24.2, 28.0]	< 0.001	HC vs. NHE: < 0.001; HC vs. HE: < 0.001; NHE vs. HE: 0.009
Blood ammonia, μmol/L	—	46.2 [29.5, 78.4]	92.3 [73.5, 119.3]	< 0.001	q < 0.001
Albumin, g/L	—	35.2 ± 5.9	31.1 ± 5.2	0.007	q = 0.014
Total bilirubin, μmol/L	—	19.8 [8.8, 32.3]	35.4 [20.9, 63.0]	0.004	q = 0.010
Cholinesterase, U/L	—	4958.3 ± 2032.0	3216.2 ± 1223.2	< 0.001	q = 0.001
Platelet count, × 10^9^/L	—	120.0 [73.0, 159.0]	68.5 [47.0, 109.0]	0.004	q = 0.010
INR	—	1.12 [1.04, 1.23]	1.27 [1.20, 1.42]	< 0.001	q = 0.001
Child–Pugh score	—	6.0 [5.0, 7.0]	9.0 [7.0, 10.0]	< 0.001	q < 0.001
Child–Pugh class, n (%)	—	A: 17 (58.6%); B: 11 (37.9%); C: 1 (3.4%)	A: 2 (6.7%); B: 19 (63.3%); C: 9 (30.0%)	< 0.001	q < 0.001
MELD score	—	10.7 ± 4.0	13.0 ± 4.2	0.036	q = 0.060

Data are mean ± standard deviation, median [interquartile range], or n (%). Overall comparisons used one-way analysis of variance, Pearson χ^2^ test, or Kruskal–Wallis test as appropriate; post hoc *p* values were adjusted with Tukey or Holm procedures. Patient-only laboratory comparisons were controlled using the Benjamini–Hochberg false-discovery-rate procedure. BH–FDR = Benjamini–Hochberg false discovery rate, HC = healthy controls, HE = hepatic encephalopathy, INR = international normalized ratio, MELD = Model for End-Stage Liver Disease, MMSE = Mini-Mental State Examination, MoCA = Montreal Cognitive Assessment, NHE = no previous overt hepatic encephalopathy and West Haven grade 0 at enrollment, PHES = Psychometric Hepatic Encephalopathy Score, TIV = total intracranial volume.

### 3.3 ALFF-to-GMV findings

ALFF-to-GMV produced 3 corrected omnibus clusters and 10 corrected directional clusters: 2 HE > HC medial temporal/thalamus-adjacent clusters and 8 HC > HE clusters involving frontal, supramarginal, precuneus, middle temporal, supplementary motor, and perirolandic regions. No ALFF-to-GMV cluster survived correction for HE > NHE, NHE > HE, NHE > HC, or HC > NHE (Figure 4; Table 3). Complete atlas-based anatomical composition of all 26 primary clusters is provided in Supplementary Table 3.

**Figure 4:**
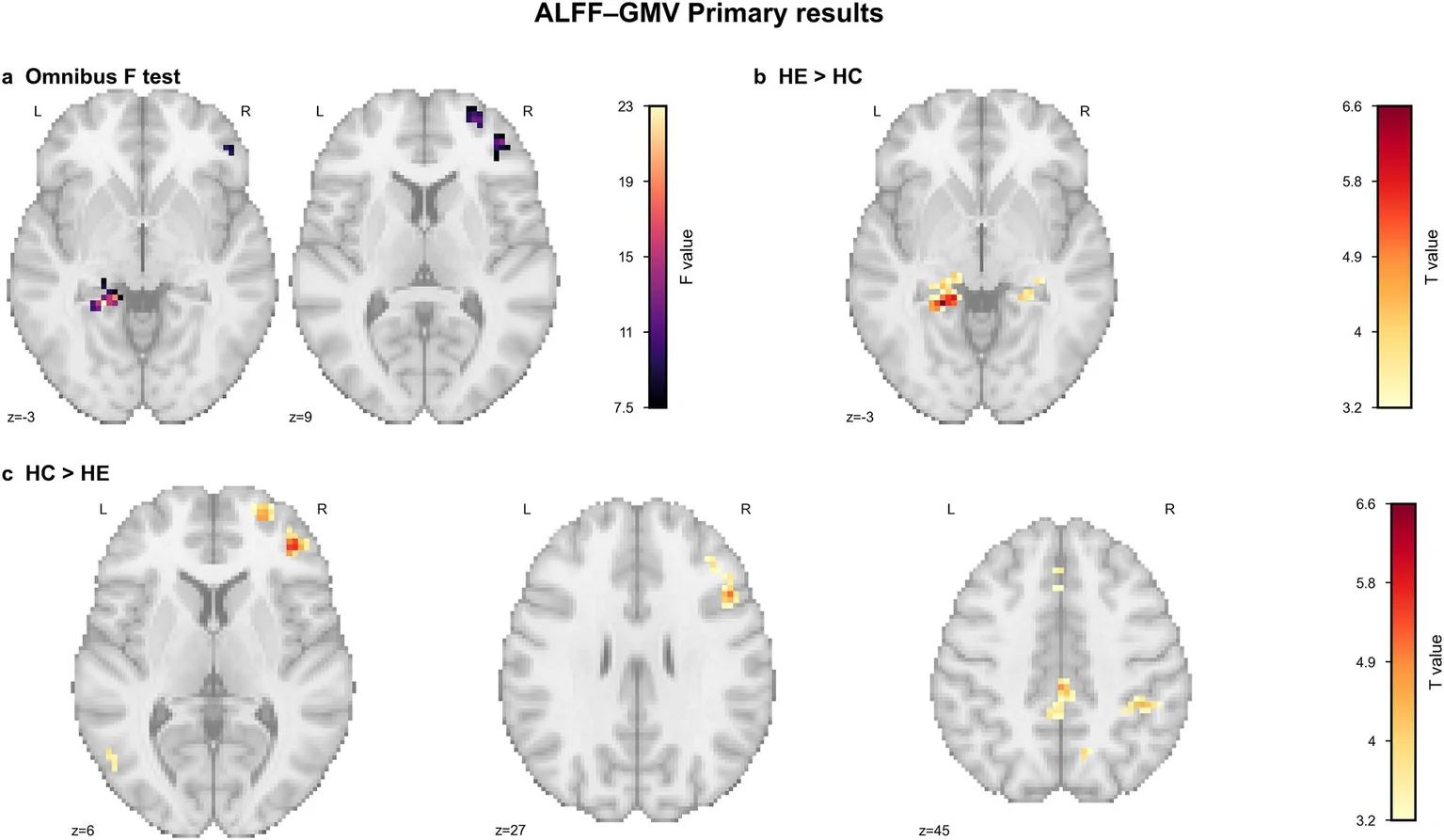
Primary ALFF-to-GMV findings. **(a)** Corrected omnibus F-test clusters. **(b)** HE > HC. **(c)** HC > HE. Maps use neurological convention. The voxel-defining threshold was *p* < 0.001 uncorrected; cluster-level family-wise error thresholds were *p* < 0.050 for the omnibus test and *p* < 0.0125 for HC-related directional contrasts. HE > NHE, NHE > HE, NHE > HC, and HC > NHE were null. ALFF = amplitude of low-frequency fluctuation, HC = healthy controls, HE = hepatic encephalopathy, NHE = no previous overt hepatic encephalopathy and West Haven grade 0 at enrollment.

**Table 3:** Formally significant primary ALFF-to-GMV clusters.

Contrast	Anatomical region	Cluster size, voxels	Cluster FWE P	Peak statistic	Peak MNI coordinates	TIV-adjusted spatial consistency
Omnibus F test	Left medial temporal region involving the parahippocampal and lingual gyri	40	< 0.001	22.92	(−21, −42, −3)	Strong spatial consistency
Omnibus F test	Right middle frontal gyrus	36	< 0.001	16.01	(42, 42, 6)	Strong spatial consistency
Omnibus F test	Right superior frontal gyrus	22	0.015	12.60	(27, 57, 9)	Strong spatial consistency
HE > HC	Left medial temporal and thalamic region	120	< 0.001	6.61	(−21, −42, −3)	Strong spatial consistency
HE > HC†	Right medial temporal region involving the hippocampus and lingual gyrus	49	< 0.001	5.22	(27, −39, −6)	Spatially shifted
HC > HE	Right middle frontal gyrus	111	< 0.001	5.56	(42, 42, 6)	Strong spatial consistency
HC > HE†	Right middle frontal gyrus	50	< 0.001	5.11	(48, 18, 27)	Strong spatial consistency
HC > HE†	Right supramarginal gyrus	45	0.001	5.06	(48, −39, 48)	Strong spatial consistency
HC > HE†	Left precuneus	142	< 0.001	4.82	(0, −30, 45)	Strong spatial consistency
HC > HE†	Left middle temporal gyrus	45	0.001	4.76	(−48, −72, 0)	Strong spatial consistency
HC > HE†	Left supplementary motor cortex	34	0.007	4.23	(−3, 27, 51)	Spatially shifted
HC > HE†	Right perirolandic region involving the postcentral and precentral gyri	32	0.010	4.14	(48, −18, 39)	Spatially shifted
HC > HE†	Right precuneus	41	0.002	4.05	(9, −63, 39)	Partial spatial consistency

The voxel-defining threshold was *p* < 0.001 uncorrected, k = 0, with 18-connectivity. Cluster-level FWE thresholds were *p* < 0.050 for the omnibus test, *p* < 0.025 for HE–NHE directional contrasts, and *p* < 0.0125 for HC-related directional contrasts. The peak statistic is F for omnibus tests and t for directional contrasts. Coordinates are in Montreal Neurological Institute space. †This directional cluster did not overlap the corrected omnibus map. ALFF = amplitude of low-frequency fluctuation, FWE = family-wise error, HC = healthy controls, HE = hepatic encephalopathy, NHE = no previous overt hepatic encephalopathy and West Haven grade 0 at enrollment, TIV = total intracranial volume.

### 3.4 Non-parametric and sensitivity findings

After TIV adjustment, 10 of 13 primary clusters for each metric retained strong or partial spatial overlap; 1 sensitivity-only cluster occurred for each metric and remained supplementary. Reconstructing the mask in the final 97 participants yielded 42,981 voxels and reproduced all 26 primary clusters. Expanded motion adjustment retained same-direction overlap in 15 of 17 directional clusters. All ReHo-to-GMV directional clusters were retained, whereas 2 smaller ALFF-to-GMV HC > HE clusters were not retained and were therefore considered more sensitive to motion-model specification. Detailed TIV, component, mask, and motion results are provided in Supplementary Tables 4–7, 9–17 and Supplementary Figures 1, 2.

Cross-metric overlap occurred only for HC > HE (31 voxels; Dice = 0.0541; Jaccard = 0.0278). Component comparisons showed same-direction functional-numerator overlap in 13 of 17 directional ratio clusters (11 with numerator-only overlap and 2 with joint numerator/GMV overlap); 4 showed neither corrected component overlap, and none showed GMV-only overlap. These threshold-dependent findings suggested closer spatial correspondence with the functional numerator than with the opposite-direction GMV contrast, but they did not establish quantitative component contributions.

Within-contrast TFCE-FWE identified HE-versus-HC effects for ReHo-to-GMV (43 positive and 2,376 negative voxels) and ALFF-to-GMV (1,558 positive and 8,280 negative voxels); neither metric showed HE-versus-NHE effects. After additional joint correction, HE-versus-HC effects remained for both metrics, whereas NHE-versus-HC evidence was weaker (Table 4; Figure 5). Of 19 archived primary-cluster masks available for direct audit, 18 were fully retained, and 1 partially overlapped the corresponding TFCE result. Cross-metric concordance and TIV-adjusted spatial consistency are summarized in Figure 6. Complete PALM and retention results are provided in Supplementary Tables 18–21.

**Table 4:** Non-parametric permutation-TFCE sensitivity analysis.

Metric	Test/direction	Within +	Within −	Across +	Across −	Omnibus result
ReHo-to-GMV	C1: NHE − HC	16	56	0	0	—
ReHo-to-GMV	C2: HE − HC	43	2,376	10	1,451	—
ReHo-to-GMV	C3: HE − NHE	0	0	0	0	—
ALFF-to-GMV	C1: NHE − HC	0	1,406	0	381	—
ALFF-to-GMV	C2: HE − HC	1,558	8,280	820	6,614	—
ALFF-to-GMV	C3: HE − NHE	0	0	0	0	—
ReHo-to-GMV	Omnibus F	—	—	—	—	1,015 voxels; 13 clusters; no directional attribution
ALFF-to-GMV	Omnibus F	—	—	—	—	3,875 voxels; 73 clusters; no directional attribution

Values are numbers of significant voxels at TFCE-FWE *p* < 0.050. “Within” denotes correction within each pairwise contrast; “Across” denotes additional joint correction across C1–C3. C1 = NHE − HC, C2 = HE − HC, C3 = HE − NHE, ALFF = amplitude of low-frequency fluctuation, FWE = family-wise error, HC = healthy controls, HE = hepatic encephalopathy, NHE = no previous overt hepatic encephalopathy and West Haven grade 0 at enrollment, ReHo = regional homogeneity, TFCE = threshold-free cluster enhancement.

**Figure 5:**
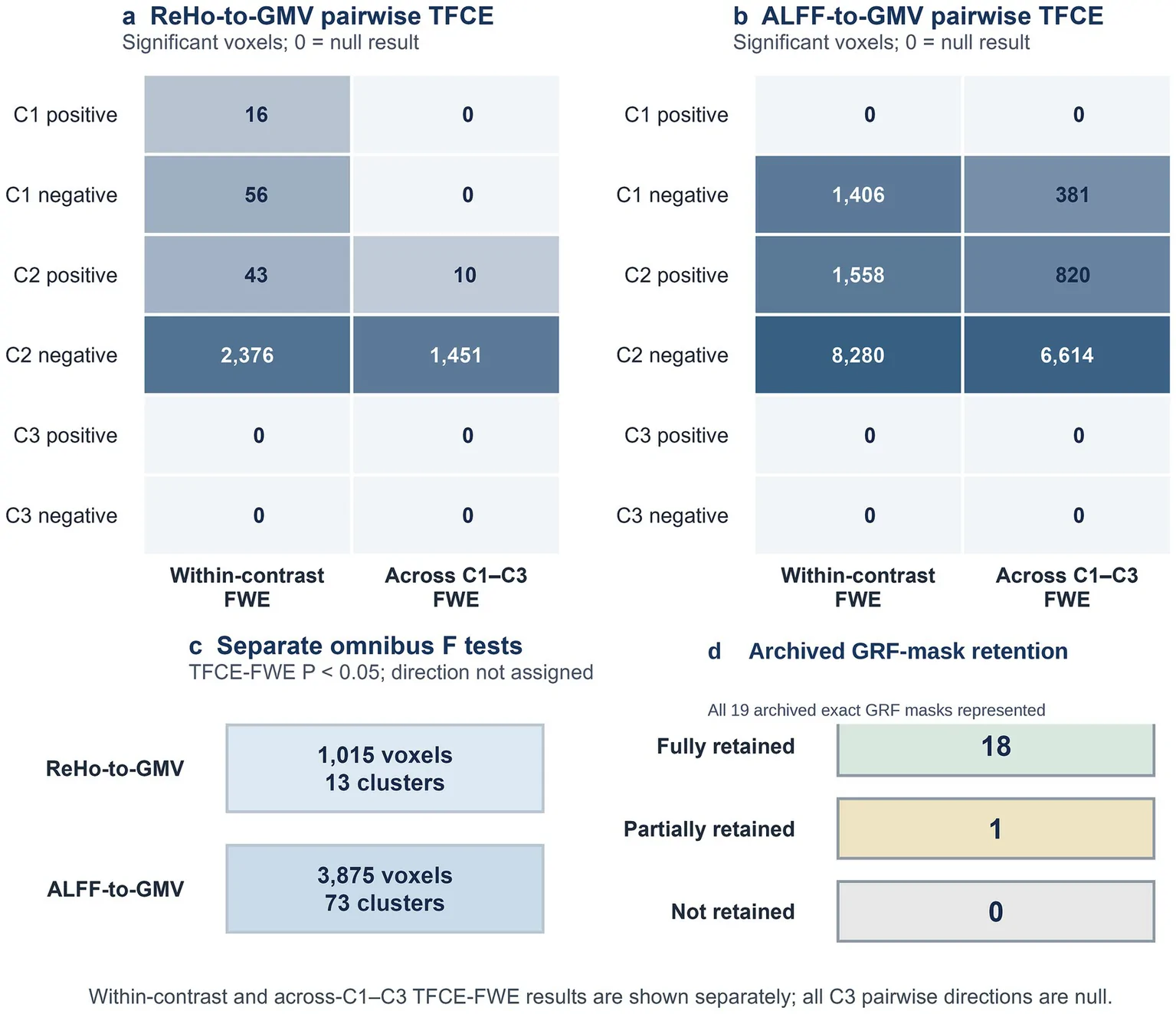
Non-parametric permutation threshold-free cluster enhancement (TFCE) sensitivity analysis. A: ReHo-to-GMV pairwise results. B: ALFF-to-GMV pairwise results. C: Separate omnibus F tests. D: Retention audit of 19 archived exact Gaussian random-field (GRF) cluster masks available for one-to-one comparison: 18 fully retained, 1 partially retained, and none without overlap. Seven of the 26 primary clusters lacked archived exact masks and were not included in this audit. C1 = NHE − HC, C2 = HE − HC, C3 = HE − NHE. Pairwise panels show positive and negative significant voxel counts under within-contrast and joint C1–C3 TFCE family-wise error correction. ALFF = amplitude of low-frequency fluctuation, HC = healthy controls, HE = hepatic encephalopathy, NHE = no previous overt hepatic encephalopathy and West Haven grade 0 at enrollment, ReHo = regional homogeneity.

**Figure 6:**
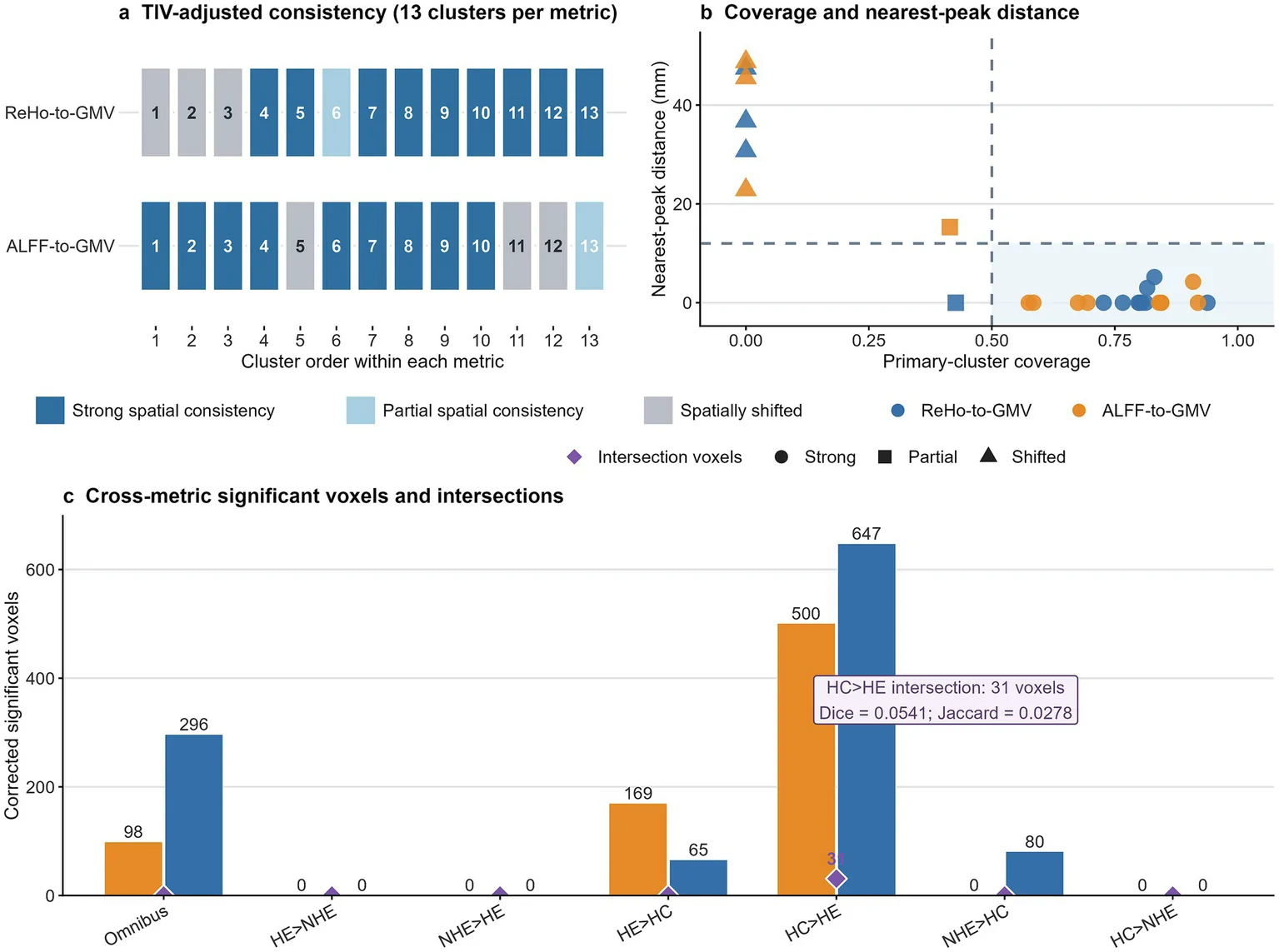
TIV-adjusted spatial consistency and cross-metric concordance. **(a)** Spatial-consistency classifications for 13 ReHo-to-GMV and 13 ALFF-to-GMV primary clusters. **(b)** Primary-cluster coverage versus distance to the nearest sensitivity-model peak; strong consistency required overlap, coverage ≥ 0.50, and distance ≤ 12 mm. **(c)** Corrected voxel counts and intersections. HC > HE maps overlapped in 31 voxels (Dice coefficient, 0.0541; Jaccard coefficient, 0.0278); omnibus and HE > HC maps did not overlap. These analyses were descriptive. ALFF = amplitude of low-frequency fluctuation, HC = healthy controls, HE = hepatic encephalopathy, NHE = no previous overt hepatic encephalopathy and West Haven grade 0 at enrollment, ReHo = regional homogeneity, TIV = total intracranial volume.

Voxel-wise local-GMV adjustment did not eliminate the principal HE-versus-HC functional effects. For ReHo, joint TFCE correction identified 722 HC > HE voxels, with no corrected NHE-versus-HC or HE-versus-NHE effect. Of the 647 voxels in the original ReHo-to-GMV HC > HE map, 415 (64.1%) were retained in the same direction (Dice = 0.606; Jaccard = 0.435), with no overlap with the original opposite-direction HE > HC map. For ALFF, the jointly corrected HE-versus-HC map comprised 1,683 HE > HC and 6,221 HC > HE voxels after direction-specific separation by the sign of the raw t statistic. Of the original ALFF-to-GMV voxels, 160 of 169 HE > HC voxels (94.7%) and all 500 HC > HE voxels (100%) were retained with concordant direction; corresponding Dice coefficients were 0.173 and 0.149, reflecting the substantially greater spatial extent of the local-GMV-adjusted ALFF effects. Opposite-direction overlap was 0. HE-versus-NHE remained null; a 4-voxel ALFF HC > NHE effect also survived joint correction. Detailed local-GMV-adjusted contrast, cluster, and spatial-overlap results are provided in Supplementary Tables 22–25.

In the exploratory within-HE phenotype sensitivity analysis, neither ReHo-to-GMV nor ALFF-to-GMV showed a TFCE-FWE-corrected difference between participants with current West Haven grade 1–2 HE and those with previous OHE who had recovered to grade 0. The minimum corrected *p* values were 0.223 for ReHo-to-GMV and 0.460 for ALFF-to-GMV, with no corrected voxels for either metric. In exploratory unadjusted subgroup comparisons, the current HE subgroup had lower PHES, MMSE, and MoCA scores than the recovered subgroup, whereas age, sex, education, mean FD, ammonia, Child-Pugh score, and MELD score showed no statistically significant differences (Supplementary Table 26).

### 3.5 Exploratory clinical findings

None of the 48 ROI-clinical associations survived within-family FDR correction; the complete analysis matrix and forest plot are provided in Supplementary Table 8 and Supplementary Figure 3. These exploratory null findings were retained as an evidential boundary and were not used to support patient-level diagnostic, staging, predictive, biomarker, or causal claims.

## 4 Discussion

### 4.1 Principal findings

This study compared 2 voxel-wise functional-to-GMV ratios across HCs, the NHE group, and an operationally defined HE group. Corrected abnormalities were concentrated in HE-versus-HC comparisons, whereas neither metric distinguished HE from NHE after correction. The absence of corrected HE-versus-NHE effects is therefore as important to the interpretation as the positive HE-versus-HC findings. ReHo-to-GMV abnormalities involved thalamic, medial frontal-cingulate, insular-basal ganglia-opercular, sensorimotor, and parietal territories; ALFF-to-GMV abnormalities involved frontal, precuneus, medial temporal, thalamus-adjacent, temporo-occipital, and sensorimotor regions. The two metrics showed little spatial overlap. Major HE-versus-HC patterns were supported by TFCE and were broadly preserved across internal sensitivity analyses, whereas exploratory clinical associations were null. Because these effects were identified primarily in HE-versus-HC comparisons and the patient groups differed in liver disease severity, they should not be interpreted as HE-specific abnormalities. The findings therefore support distributed, metric-dependent group-level alterations rather than a single HE-specific imaging signature or validated biomarker.

### 4.2 ReHo-to-GMV abnormalities

The ReHo-to-GMV pattern suggests regionally heterogeneous alteration of local synchrony relative to GMV. The thalamus is a relay and higher-order integrative hub within distributed cortical networks (25), whereas medial frontal, cingulate, insular, basal ganglia, and sensorimotor systems contribute to attention, salience allocation, behavioral switching, and psychomotor performance. One possible literature-based explanation is that hyperammonemia-related astrocytic stress and systemic inflammation may affect neurotransmitter cycling, energy metabolism, extracellular homeostasis, and neurovascular regulation (26–28). Such processes could contribute to regionally heterogeneous imaging patterns; however, none of these mechanisms was directly measured in the present study.

The component analysis provided important interpretive context. Corrected ReHo-to-GMV clusters more often overlapped the same-direction ReHo numerator map than the opposite-direction GMV map. This pattern is compatible with a substantial functional contribution under the applied thresholds, but it does not quantify component weights or establish that GMV is unimportant. Conversely, the absence of corrected component overlap for some ratio clusters leaves their numerator-denominator basis unresolved. These observations reinforce the need to interpret ratio maps as operational spatial summaries rather than direct measures of structure–function physiology.

### 4.3 ALFF-to-GMV abnormalities

ALFF-to-GMV was lower in HE in frontal, precuneus, temporal, and sensorimotor regions and higher in medial temporal and thalamus-adjacent regions. Frontal and precuneus abnormalities are common, although not directionally consistent, in previous resting-state studies of cirrhosis and minimal HE (8–10, 29). The medial temporal findings may reflect altered spontaneous activity, GMV, or both; a higher ratio should not be interpreted as enhanced neuronal function or compensation. Previous literature has implicated astrocyte-neuron metabolic disturbance, oxidative stress, inflammatory signaling, and altered neurovascular regulation in HE. These mechanisms provide possible biological context for the observed group-level patterns (30, 31), but they were not directly measured and cannot be distinguished by the present ratio analysis. As with ReHo-to-GMV, component comparisons showed more frequent corrected overlap with the ALFF numerator than with the GMV contrast in the opposite direction. This closer spatial correspondence is informative but threshold-dependent. It should not be read as a formal decomposition of the ratio, and it does not exclude more subtle or spatially shifted structural effects. Given the absence of FDR-corrected imaging-clinical associations, these mechanistic interpretations should be regarded as hypothesis-generating rather than explanatory evidence. The strongest inference supported by the present data is the presence of distributed group-level imaging differences, rather than evidence for a specific biological mechanism.

### 4.4 Cross-metric concordance and robustness

The limited ReHo-to-GMV/ALFF-to-GMV concordance is expected because ReHo measures local temporal concordance, whereas ALFF measures within-voxel fluctuation magnitude (7). A region may therefore show amplitude change without altered neighborhood synchrony, or vice versa. In the present data, corrected maps overlapped only for HC > HE and only across 31 voxels. The two ratios consequently provided partly different spatial descriptions, but this limited overlap does not demonstrate mechanistic independence or complementary biological pathways.

The TFCE analysis showed that the main HE-versus-HC patterns were not solely dependent on the original Gaussian random-field cluster procedure. Non-parametric support was particularly extensive for ALFF-to-GMV and remained substantial after joint correction across pairwise contrasts. TIV adjustment, final-sample mask reconstruction, and expanded motion adjustment preserved much of the primary spatial pattern. In particular, the ReHo-to-GMV directional findings and the larger HE-versus-HC ALFF-to-GMV effects retained spatial support after expanded motion adjustment, whereas two smaller ALFF-to-GMV HC > HE clusters were not retained. These latter findings should therefore be interpreted more cautiously as motion-model-sensitive. Nevertheless, all of these analyses reused the same participants and data and therefore constitute internal robustness checks rather than replication or external validation.

The local-GMV-adjusted models provided an additional denominator-focused robustness check. ReHo retained appreciable same-direction correspondence with the original ratio map, whereas the original ALFF-to-GMV abnormalities were almost completely contained within more spatially extensive local-GMV-adjusted ALFF effects. This pattern argues against a purely denominator-driven explanation of the principal HE-versus-HC findings, while the incomplete or asymmetric spatial overlap also shows that division-based ratios and covariate-adjusted functional models are not interchangeable representations.

### 4.5 HE-versus-NHE null findings and clinical interpretation

The absence of corrected HE-versus-NHE differences is an important evidential boundary, but it does not establish statistical equivalence. Rather, it indicates that the present operationally defined patient groups could not be separated using these ratio measures after multiple-comparison correction. The operational HE group combined participants with current West Haven grade 1–2 HE and participants with previous OHE who had recovered to West Haven grade 0 at enrollment, thereby introducing heterogeneity with respect to current clinical status and previous HE history. Current guidelines emphasize distinctions among covert HE, current overt manifestations, and previous episodes (16, 32). In addition, the NHE group was defined by the absence of previous OHE and West Haven grade 0 at enrollment rather than by PHES, and therefore may include patients with psychometric abnormalities compatible with minimal HE. These features may have reduced the expectation of a sharp imaging boundary between NHE and HE. Because the current-versus-recovered analysis was exploratory and based on a modest sample, and detailed previous OHE burden, cirrhosis etiology, transjugular intrahepatic portosystemic shunt (TIPS) status, and HE-directed treatment exposure were not modeled separately, the primary imaging contrasts should still be interpreted as comparisons between operational groups rather than state-specific HE effects.

An exploratory within-HE analysis likewise did not identify corrected ReHo-to-GMV or ALFF-to-GMV differences between participants with current West Haven grade 1–2 HE and those with previous OHE who had recovered to grade 0, despite clear between-subgroup differences in cognitive performance. This null imaging result does not establish equivalence, particularly given the modest subgroup sizes, but it indicates that the present ratio measures did not provide corrected separation of current and recovered HE phenotypes in this sample.

None of the exploratory ROI associations with cognition, ammonia, Child-Pugh score, or MELD survived FDR correction. This null result is clinically important because it limits the patient-level interpretability of the ratio measures and argues against treating them as established diagnostic, staging, prognostic, or monitoring biomarkers. A single ammonia measurement and global liver-severity scores may also be insensitive to regional and time-varying brain dysfunction. Accordingly, the present findings are best interpreted as group-level imaging differences rather than clinically validated individual-level markers. Clinical utility will require independent cohorts, prespecified endpoints, and direct evaluation of discrimination, calibration, longitudinal change, and test–retest reliability.

### 4.6 Strengths and limitations

Strengths of the study include parallel analysis of two functional-to-GMV metrics, a strict standard/sample/participant GMV mask to reduce small-denominator exposure, explicit contrast-family correction, non-parametric TFCE sensitivity analysis, final-sample mask reconstruction, expanded motion adjustment, voxel-wise local-GMV adjustment, and direct evaluation of numerator/denominator spatial context. The analysis also retained and explicitly reported corrected HE-versus-NHE and clinical-association null findings rather than treating them as secondary failures.

Several limitations remain. Ratio measures are susceptible to denominator variability, non-normality, heteroscedasticity, and mathematical coupling (13, 14). The strict mask reduced exposure to very small denominators but cannot remove these mathematical properties. Direct voxel-wise local-GMV adjustment reduced, but did not eliminate, uncertainty related to ratio formulation: the ReHo map retained 64.1% of the original HC > HE ratio-defined voxels, whereas ALFF retained 94.7% of the original HE > HC and 100% of the original HC > HE voxels, but the adjusted ALFF effects were substantially more extensive and therefore had low symmetric Dice coefficients. These sensitivity results argue against a purely denominator-driven explanation without establishing independence from GMV or equivalence between model formulations. Functional maps were smoothed at 4 mm, whereas GMV was smoothed at 8 mm; the resulting difference in effective smoothness may affect cluster extent and localization, and a matched-kernel sensitivity analysis was unavailable.

Susceptibility-distortion correction and scrubbing were not performed, limiting confidence particularly in orbitofrontal, insular, and medial temporal regions and leaving residual motion as a concern. Mean FD differed among groups, and two smaller ALFF-to-GMV HC > HE clusters were not retained after expanded-motion adjustment. The single-center, cross-sectional design and clinically heterogeneous HE group limit phenotype-specific interpretation. The HE group included both participants with current West Haven grade 1–2 HE and participants with previous OHE who had recovered to grade 0, whereas the NHE group was defined by OHE history and West Haven grade rather than by a normal PHES. Same-sample ROI selection, the limited clinical sample, and the absence of external validation or test–retest data further restrict generalizability. The absence of FDR-corrected imaging-clinical associations further limits inference regarding the clinical relevance of the observed group-level differences. Future studies should prioritize validation of these findings in clinically more homogeneous subgroups defined prospectively by current West Haven status, previous OHE history, and standardized criteria for minimal HE. Although current-versus-recovered HE status was examined in an exploratory within-HE analysis, cirrhosis etiology, TIPS status, detailed previous OHE burden, and HE-directed treatment exposure were not incorporated as separate imaging-model factors, so residual clinical confounding cannot be excluded. Multiplicity was controlled within metric-specific, analysis-defined hypothesis families rather than under a single experiment-wide alpha allocation spanning both ratio metrics and all secondary analyses. All robustness analyses reused the same cohort and should not be interpreted as independent replication.

## 5 Conclusion

ReHo-to-GMV and ALFF-to-GMV provided distributed and partly distinct neuroimaging descriptions of group-level brain differences in cirrhosis, with corrected effects concentrated primarily in comparisons between HE and HC and no corrected HE-versus-NHE difference or validated clinical association. These ratios are best regarded as operational spatial measures of local functional signal relative to GMV rather than direct physiological coupling measures. Local-GMV-adjusted sensitivity analyses further indicated that the principal HE-versus-HC abnormalities were not solely attributable to variation in the GMV denominator. Independent multicenter and longitudinal studies should stratify participants by current West Haven status and previous OHE history, retain direct numerator and denominator analyses, replicate direct comparisons of ratio-based and local-GMV-adjusted functional models, and evaluate clinical performance in external samples.

## Data Availability

The raw data supporting the conclusions of this article will be made available by the authors, without undue reservation.
