# Supplementary material for: Voxel-wise functional-to-gray matter volume ratios reveal distinct brain patterns in cirrhosis stratified by hepatic encephalopathy history and clinical status
Source: Front Neurol. 2026 Oct 19;17:1969935. doi: 10.3389/fneur.2026.1969935 (PMC13724522; doi:10.3389/fneur.2026.1969935)
Supplement: Supplementary file 1 [file Table_1.docx]

Supplementary Material

# S1. Supplementary Methods, Preprocessing, Quality Control, and Primary Sensitivity Analyses

The primary Gaussian random-field whole-brain analyses are described in the main manuscript. The supplementary analyses below provide additional quality-control evidence, anatomical detail, sensitivity analyses, component-level spatial context, and complete exploratory clinical results. The NHE label denotes an operational group with no previous OHE and West Haven grade 0 at enrollment; PHES was not used to exclude minimal HE or to redefine the imaging groups.

Resting-state EPI used a descending interleaved slice order: 33, 31, 29, 27, 25, 23, 21, 19, 17, 15, 13, 11, 9, 7, 5, 3, 1, followed by 32, 30, 28, 26, 24, 22, 20, 18, 16, 14, 12, 10, 8, 6, 4, and 2. Slice-timing correction used slice 33 as the reference slice.

TIV-adjusted sensitivity models added total intracranial volume to age, sex, education, and mean Jenkinson framewise displacement while retaining the primary mask, contrasts, and inferential thresholds. For each corrected primary cluster, spatial consistency was summarized by overlap with the same-direction TIV-adjusted map, primary-cluster coverage, and the distance from the primary peak to the nearest corrected sensitivity-model peak. Strong spatial consistency required nonzero overlap, coverage ≥0.50, and peak distance ≤12 mm; other overlapping clusters were classified as partial spatial consistency, corrected sensitivity voxels without overlap were classified as spatially shifted, and directions without corrected sensitivity voxels were classified as not surviving the sensitivity model. Sensitivity-only clusters were not used to replace primary findings or define clinical ROIs.

Primary clusters were labeled with the Neuromorphometrics atlas in 3 × 3 × 3 mm³ space. Complete corrected clusters served as ROIs. Eight imaging-defined core ROIs were selected before clinical association testing from directional clusters that showed strong or partial TIV-adjusted spatial consistency and overlapped the corresponding corrected omnibus map. Clinical variables were not used for ROI selection.

Post hoc component analyses evaluated szReHo (the standardized ReHo map after 4-mm smoothing), zALFF, and modulated normalized GMV in the final 97 participants using the same three-group design, covariates, mask, contrasts, and within-component family-wise error thresholds as the primary ratio analyses. Ratio maps were compared with same-direction functional numerator maps and opposite-direction GMV maps using voxel intersections, Dice and Jaccard coefficients, cluster coverage, and peak distances. These comparisons were threshold dependent and descriptive. Same-sample adjusted means and HC3 confidence intervals were reported without additional within-cluster significance testing.

Two additional sensitivity analyses reconstructed the strict common gray-matter mask using the final 97 participants and re-estimated the primary models after adding centered maximum translation and maximum rotation to mean framewise displacement. Design rank, condition diagnostics, and variance-inflation factors were assessed before estimation.

Exploratory clinical association analyses included 59 patients and eight core ROIs. Rank-based partial Spearman correlations adjusted for group, sex, age, education, and mean framewise displacement; a sensitivity model additionally included ranked TIV. Percentile 95% confidence intervals were estimated from 5,000 group-stratified bootstrap resamples. Benjamini–Hochberg FDR correction was applied within the PHES, MoCA/MMSE, and disease-severity families.

Supplementary Table 1. Participant flow and motion-quality-control verification.

| **Group** | **Initial N** | **Excluded N** | **Final N** | **Exclusion basis** |
| --- | --- | --- | --- | --- |
| HC | 42 | 4 | 38 | Exceeded ≥1 motion threshold |
| NHE | 37 | 8 | 29 | Exceeded ≥1 motion threshold |
| HE | 39 | 9 | 30 | Exceeded ≥1 motion threshold |
| Total | 118 | 21 | 97 | Aggregate motion exclusions |

Note.—Participants were excluded if they exceeded at least one analysis-defined motion threshold: translation >3 mm, rotation >3°, or mean Jenkinson framewise displacement >0.20 mm. HC = healthy controls, HE = hepatic encephalopathy, NHE = no previous overt hepatic encephalopathy and West Haven grade 0 at enrollment.

Supplementary Table 2. Complete patient-group clinical and laboratory characteristics.

| **Variable** | **NHE** | **HE** | **Presentation** | **Statistical test** | **Statistic** | **P value** | **BH-FDR q** |
| --- | --- | --- | --- | --- | --- | --- | --- |
| Blood ammonia, μmol/L | 46.2 [29.5, 78.4] | 92.3 [73.5, 119.3] | Median [IQR] | Mann-Whitney U test | 179.0000 | <0.001 | <0.001 |
| Serum sodium, mmol/L | 141.4 [139.7, 144.6] | 140.8 [138.2, 142.4] | Median [IQR] | Mann-Whitney U test | 551.0000 | 0.080 | 0.120 |
| Creatinine, μmol/L | 63.1 [57.5, 77.6] | 66.5 [56.8, 84.8] | Median [IQR] | Mann-Whitney U test | 411.0000 | 0.722 | 0.722 |
| Albumin, g/L | 35.2 ± 5.9 | 31.1 ± 5.2 | Mean ± SD | Independent-samples t test | 2.8010 | 0.007 | 0.014 |
| Total bilirubin, μmol/L | 19.8 [8.8, 32.3] | 35.4 [20.9, 63.0] | Median [IQR] | Mann-Whitney U test | 246.5000 | 0.004 | 0.010 |
| ALT, U/L | 29.0 [22.0, 40.6] | 24.1 [15.2, 39.5] | Median [IQR] | Mann-Whitney U test | 523.0000 | 0.184 | 0.255 |
| AST, U/L | 27.0 [21.0, 49.0] | 33.0 [23.0, 54.8] | Median [IQR] | Mann-Whitney U test | 379.0000 | 0.400 | 0.433 |
| Cholinesterase, U/L | 4958.3 ± 2032.0 | 3216.2 ± 1223.2 | Mean ± SD | Welch t test | 3.9730 | <0.001 | 0.001 |
| eGFR, mL/min/1.73 m² | 103.1 [85.2, 113.0] | 97.8 [79.9, 111.3] | Median [IQR] | Mann-Whitney U test | 490.0000 | 0.409 | 0.433 |
| White blood cell count, ×10⁹/L | 4.44 ± 1.45 | 4.08 ± 1.60 | Mean ± SD | Independent-samples t test | 0.9130 | 0.365 | 0.433 |
| Red blood cell count, ×10¹²/L | 3.86 ± 0.84 | 3.40 ± 0.78 | Mean ± SD | Independent-samples t test | 2.1830 | 0.033 | 0.060 |
| Hemoglobin, g/L | 115.7 ± 25.8 | 107.4 ± 26.5 | Mean ± SD | Independent-samples t test | 1.2260 | 0.225 | 0.289 |
| Platelet count, ×10⁹/L | 120.0 [73.0, 159.0] | 68.5 [47.0, 109.0] | Median [IQR] | Mann-Whitney U test | 625.5000 | 0.004 | 0.010 |
| Prothrombin time, s | 12.90 [11.90, 14.00] | 14.45 [13.95, 16.08] | Median [IQR] | Mann-Whitney U test | 195.0000 | <0.001 | 0.001 |
| INR | 1.12 [1.04, 1.23] | 1.27 [1.20, 1.42] | Median [IQR] | Mann-Whitney U test | 203.5000 | <0.001 | 0.001 |
| Child-Pugh score | 6.0 [5.0, 7.0] | 9.0 [7.0, 10.0] | Median [IQR] | Mann-Whitney U test | 143.5000 | <0.001 | <0.001 |
| MELD score | 10.7 ± 4.0 | 13.0 ± 4.2 | Mean ± SD | Independent-samples t test | -2.1420 | 0.036 | 0.060 |
| Child-Pugh class, n (%) | A: 17 (58.6%); B: 11 (37.9%); C: 1 (3.4%) | A: 2 (6.7%); B: 19 (63.3%); C: 9 (30.0%) | n (%) | Fisher exact test |  | <0.001 | <0.001 |

Note.—Values are mean ± SD, median [IQR], or n (%), as indicated. BH-FDR = Benjamini–Hochberg false discovery rate; eGFR = estimated glomerular filtration rate; INR = international normalized ratio; MELD = Model for End-Stage Liver Disease.

Supplementary Table 3. Complete anatomical composition of the 26 primary clusters.

| **Final cluster ID** | **Metric** | **Contrast** | **Cluster ID** | **Primary anatomical label** | **Hemisphere** | **Primary label, %** | **Secondary anatomical labels** | **Unlabeled, %** | **Peak atlas region** | **Multiregional** | **TIV-adjusted spatial consistency** |
| --- | --- | --- | --- | --- | --- | --- | --- | --- | --- | --- | --- |
| ReHo_Omnibus_C001 | ReHo-to-GMV | Omnibus F test | 1 | Right Thalamus Proper | Right | 90.3 | Right Thalamus Proper (90.3%); Right Lateral Ventricle (6.5%); Right Cerebral White Matter (3.2%) | 0.0 | Right Thalamus Proper | No | Spatially shifted |
| ReHo_Omnibus_C002 | ReHo-to-GMV | Omnibus F test | 2 | Left ACgG anterior cingulate gyrus | Left | 30.0 | Left ACgG anterior cingulate gyrus (30.0%); Right MSFG superior frontal gyrus medial segment (25.0%); Left MSFG superior frontal gyrus medial segment (12.5%); Right ACgG anterior cingulate gyrus (10.0%) | 22.5 | Outside atlas or unlabeled | Yes | Spatially shifted |
| ReHo_Omnibus_C003 | ReHo-to-GMV | Omnibus F test | 3 | Right Cerebral White Matter | Right | 37.1 | Right Cerebral White Matter (37.1%); Right PoG postcentral gyrus (31.4%); Right PrG precentral gyrus (31.4%) | 0.0 | Right PrG precentral gyrus | Yes | Spatially shifted |
| ReHo_Omnibus_C004 | ReHo-to-GMV | Omnibus F test | 4 | Right AIns anterior insula | Right | 53.2 | Right AIns anterior insula (53.2%); Right Cerebral White Matter (11.7%); Right FO frontal operculum (9.1%); Right Putamen (7.8%); Right LOrG lateral orbital gyrus (5.2%) | 2.6 | Right FO frontal operculum | No | Strong spatial consistency |
| ReHo_Omnibus_C006 | ReHo-to-GMV | Omnibus F test | 6 | Right Cerebral White Matter | Right | 42.1 | Right Cerebral White Matter (42.1%); Right STG superior temporal gyrus (18.4%); Right TTG transverse temporal gyrus (18.4%); Right PP planum polare (13.2%); Right CO central operculum (5.3%) | 2.6 | Right Cerebral White Matter | No | Strong spatial consistency |
| ReHo_Omnibus_C007 | ReHo-to-GMV | Omnibus F test | 7 | Left AIns anterior insula | Left | 29.3 | Left AIns anterior insula (29.3%); Left Putamen (24.0%); Left PIns posterior insula (21.3%); Left Cerebral White Matter (20.0%); Left Pallidum (1.3%) | 4.0 | Left Putamen | Yes | Partial spatial consistency |
| ReHo_HE>HC_C001 | ReHo-to-GMV | HE > HC | 1 | Right Thalamus Proper | Right | 70.8 | Right Thalamus Proper (70.8%); Right Cerebral White Matter (12.3%); Right Caudate (9.2%); Right Lateral Ventricle (7.7%) | 0.0 | Right Thalamus Proper | No | Strong spatial consistency |
| ReHo_HC>HE_C001 | ReHo-to-GMV | HC > HE | 1 | Left ACgG anterior cingulate gyrus | Left | 50.5 | Left ACgG anterior cingulate gyrus (50.5%); Right MSFG superior frontal gyrus medial segment (13.1%); Left MSFG superior frontal gyrus medial segment (13.1%); Right ACgG anterior cingulate gyrus (8.1%); Left Cerebral White Matter (1.0%) | 14.1 | Outside atlas or unlabeled | No | Strong spatial consistency |
| ReHo_HC>HE_C002 | ReHo-to-GMV | HC > HE | 2 | Right Cerebral White Matter | Right | 27.2 | Right Cerebral White Matter (27.2%); Right PoG postcentral gyrus (22.8%); Right SMG supramarginal gyrus (20.6%); Right PrG precentral gyrus (16.3%); Right SPL superior parietal lobule (13.0%) | 0.0 | Right PrG precentral gyrus | Yes | Strong spatial consistency |
| ReHo_HC>HE_C003 | ReHo-to-GMV | HC > HE | 3 | Right Cerebral White Matter | Right | 26.9 | Right Cerebral White Matter (26.9%); Right AIns anterior insula (25.6%); Right Putamen (21.9%); Right PIns posterior insula (9.2%); Right FO frontal operculum (5.5%) | 2.1 | Right AIns anterior insula | Yes | Strong spatial consistency |
| ReHo_HC>HE_C004 | ReHo-to-GMV | HC > HE | 4 | Right Cerebral White Matter | Right | 24.0 | Right Cerebral White Matter (24.0%); Right STG superior temporal gyrus (20.0%); Right CO central operculum (18.0%); Right PP planum polare (15.0%); Right TTG transverse temporal gyrus (13.0%) | 3.0 | Right Cerebral White Matter | Yes | Strong spatial consistency |
| ReHo_HC>HE_C006 | ReHo-to-GMV | HC > HE | 6 | Left AIns anterior insula | Left | 33.0 | Left AIns anterior insula (33.0%); Left Cerebral White Matter (22.0%); Left Putamen (21.2%); Left PIns posterior insula (16.1%); Left TMP temporal pole (1.7%) | 4.2 | Left Cerebral White Matter | Yes | Strong spatial consistency |
| ReHo_NHE>HC_C001 | ReHo-to-GMV | NHE > HC | 1 | Right PrG precentral gyrus | Right | 48.8 | Right PrG precentral gyrus (48.8%); Right PoG postcentral gyrus (33.8%); Right SPL superior parietal lobule (10.0%) | 7.5 | Right PrG precentral gyrus | Yes | Strong spatial consistency |
| ALFF_Omnibus_C001 | ALFF-to-GMV | Omnibus F test | 1 | Left Cerebral White Matter | Left | 45.0 | Left Cerebral White Matter (45.0%); Left PHG parahippocampal gyrus (20.0%); Left LiG lingual gyrus (15.0%); Left Hippocampus (7.5%); Left Thalamus Proper (5.0%) | 0.0 | Left Cerebral White Matter | Yes | Strong spatial consistency |
| ALFF_Omnibus_C002 | ALFF-to-GMV | Omnibus F test | 2 | Right MFG middle frontal gyrus | Right | 41.7 | Right MFG middle frontal gyrus (41.7%); Right TrIFG triangular part of the inferior frontal gyrus (38.9%); Right Cerebral White Matter (19.4%) | 0.0 | Right MFG middle frontal gyrus | Yes | Strong spatial consistency |
| ALFF_Omnibus_C010 | ALFF-to-GMV | Omnibus F test | 10 | Right SFG superior frontal gyrus | Right | 63.6 | Right SFG superior frontal gyrus (63.6%); Right MFG middle frontal gyrus (36.4%) | 0.0 | Right MFG middle frontal gyrus | Yes | Strong spatial consistency |
| ALFF_HE>HC_C001 | ALFF-to-GMV | HE > HC | 1 | Left Cerebral White Matter | Left | 45.0 | Left Cerebral White Matter (45.0%); Left Thalamus Proper (14.2%); Left PHG parahippocampal gyrus (13.3%); Left Hippocampus (10.0%); Left LiG lingual gyrus (8.3%) | 0.0 | Left Cerebral White Matter | No | Strong spatial consistency |
| ALFF_HE>HC_C004 | ALFF-to-GMV | HE > HC | 4 | Right Cerebral White Matter | Right | 42.9 | Right Cerebral White Matter (42.9%); Right Hippocampus (26.5%); Right LiG lingual gyrus (12.2%); Right Ventral DC (6.1%); Right PHG parahippocampal gyrus (6.1%) | 0.0 | Right Cerebral White Matter | Yes | Spatially shifted |
| ALFF_HC>HE_C001 | ALFF-to-GMV | HC > HE | 1 | Right MFG middle frontal gyrus | Right | 46.0 | Right MFG middle frontal gyrus (46.0%); Right SFG superior frontal gyrus (20.7%); Right TrIFG triangular part of the inferior frontal gyrus (19.8%); Right Cerebral White Matter (11.7%) | 1.8 | Right MFG middle frontal gyrus | Yes | Strong spatial consistency |
| ALFF_HC>HE_C002 | ALFF-to-GMV | HC > HE | 2 | Right MFG middle frontal gyrus | Right | 44.0 | Right MFG middle frontal gyrus (44.0%); Right OpIFG opercular part of the inferior frontal gyrus (40.0%); Right Cerebral White Matter (16.0%) | 0.0 | Right OpIFG opercular part of the inferior frontal gyrus | Yes | Strong spatial consistency |
| ALFF_HC>HE_C003 | ALFF-to-GMV | HC > HE | 3 | Right SMG supramarginal gyrus | Right | 55.6 | Right SMG supramarginal gyrus (55.6%); Right SPL superior parietal lobule (31.1%); Right Cerebral White Matter (13.3%) | 0.0 | Right Cerebral White Matter | Yes | Strong spatial consistency |
| ALFF_HC>HE_C004 | ALFF-to-GMV | HC > HE | 4 | Left PCu precuneus | Left | 30.3 | Left PCu precuneus (30.3%); Right PCu precuneus (16.9%); Left PCgG posterior cingulate gyrus (14.1%); Right PCgG posterior cingulate gyrus (8.4%); Right Cerebral White Matter (4.9%) | 6.3 | Left PCgG posterior cingulate gyrus | No | Strong spatial consistency |
| ALFF_HC>HE_C005 | ALFF-to-GMV | HC > HE | 5 | Left MTG middle temporal gyrus | Left | 51.1 | Left MTG middle temporal gyrus (51.1%); Left IOG inferior occipital gyrus (42.2%); Left Cerebral White Matter (6.7%) | 0.0 | Left IOG inferior occipital gyrus | Yes | Strong spatial consistency |
| ALFF_HC>HE_C012 | ALFF-to-GMV | HC > HE | 12 | Left SMC supplementary motor cortex | Left | 20.6 | Left SMC supplementary motor cortex (20.6%); Left MSFG superior frontal gyrus medial segment (17.6%); Right SMC supplementary motor cortex (8.8%); Left Cerebral White Matter (2.9%) | 50.0 | Left MSFG superior frontal gyrus medial segment | No | Spatially shifted |
| ALFF_HC>HE_C013 | ALFF-to-GMV | HC > HE | 13 | Right Cerebral White Matter | Right | 50.0 | Right Cerebral White Matter (50.0%); Right PoG postcentral gyrus (25.0%); Right PrG precentral gyrus (18.8%); Right SMG supramarginal gyrus (6.2%) | 0.0 | Right Cerebral White Matter | Yes | Spatially shifted |
| ALFF_HC>HE_C019 | ALFF-to-GMV | HC > HE | 19 | Right PCu precuneus | Right | 61.0 | Right PCu precuneus (61.0%); Right Cerebral White Matter (29.3%); Right Cun cuneus (9.8%) | 0.0 | Right Cerebral White Matter | Yes | Partial spatial consistency |

Note.—Percentages indicate atlas overlap within each cluster. TIV = total intracranial volume. Some “Cerebral White Matter” labels reflect nearest-atlas assignments after atlas resampling and boundary overlap within the explicit gray-matter analysis mask; they do not indicate that a separate white-matter analysis was performed.

Supplementary Table 4. ReHo-to-GMV contrast-level TIV sensitivity.

| **Contrast** | **Primary significant voxels** | **Sensitivity significant voxels** | **Intersection voxels** | **Union voxels** | **Primary coverage** | **Sensitivity coverage** | **Dice** | **Jaccard** | **Direction consistent** | **Primary and sensitivity maps significant** | **Sensitivity-only cluster** | **Both maps null** |
| --- | --- | --- | --- | --- | --- | --- | --- | --- | --- | --- | --- | --- |
| Omnibus F test | 296 | 135 | 122 | 309 | 0.41 | 0.90 | 0.5661 | 0.3948 | Yes | Yes | No | No |
| HE > NHE | 0 | 0 | 0 | 0 |  |  |  |  | No | No | No | Yes |
| NHE > HE | 0 | 0 | 0 | 0 |  |  |  |  | No | No | No | Yes |
| HE > HC | 65 | 65 | 61 | 69 | 0.94 | 0.94 | 0.9385 | 0.8841 | Yes | Yes | No | No |
| HC > HE | 647 | 555 | 514 | 688 | 0.79 | 0.93 | 0.8552 | 0.7471 | Yes | Yes | No | No |
| NHE > HC | 80 | 76 | 65 | 91 | 0.81 | 0.86 | 0.8333 | 0.7143 | Yes | Yes | No | No |
| HC > NHE | 0 | 67 | 0 | 67 |  | 0 | 0 | 0 | No | No | Yes | No |

Note.—Coverage, Dice, and Jaccard values are descriptive spatial-comparison measures. TIV = total intracranial volume.

Supplementary Table 5. ReHo-to-GMV cluster-level TIV sensitivity.

| **Contrast** | **Cluster ID** | **Extent, voxels** | **Cluster FWE P** | **Primary peak MNI** | **Overlap, voxels** | **Primary coverage** | **Sensitivity peak MNI** | **Nearest-peak distance, mm** | **Overlaps sensitivity map** | **Distance ≤12 mm** | **TIV-adjusted spatial consistency** |
| --- | --- | --- | --- | --- | --- | --- | --- | --- | --- | --- | --- |
| Omnibus F test | 1 | 31 | 0.046 | (15, -18, 15) | 0 | 0 | (30, 12, 0) | 36.7423 | No | No | Spatially shifted |
| Omnibus F test | 2 | 40 | 0.016 | (0, 51, 6) | 0 | 0 | (36, 24, -9) | 47.4342 | No | No | Spatially shifted |
| Omnibus F test | 3 | 35 | 0.028 | (45, -12, 36) | 0 | 0 | (51, -9, 6) | 30.7409 | No | No | Spatially shifted |
| Omnibus F test | 4 | 77 | <0.001 | (45, 12, -3) | 59 | 0.77 | (45, 12, -3) | 0 | Yes | Yes | Strong spatial consistency |
| Omnibus F test | 6 | 38 | 0.020 | (57, -3, -3) | 31 | 0.82 | (57, -3, -6) | 3 | Yes | Yes | Strong spatial consistency |
| Omnibus F test | 7 | 75 | <0.001 | (-27, -9, -6) | 32 | 0.43 | (-27, -9, -6) | 0 | Yes | Yes | Partial spatial consistency |
| HE > HC | 1 | 65 | 0.006 | (15, -18, 15) | 61 | 0.94 | (15, -18, 15) | 0 | Yes | Yes | Strong spatial consistency |
| HC > HE | 1 | 99 | <0.001 | (0, 51, 6) | 72 | 0.73 | (0, 51, 6) | 0 | Yes | Yes | Strong spatial consistency |
| HC > HE | 2 | 92 | <0.001 | (45, -12, 36) | 74 | 0.80 | (45, -12, 36) | 0 | Yes | Yes | Strong spatial consistency |
| HC > HE | 3 | 238 | <0.001 | (45, 12, -6) | 190 | 0.80 | (45, 12, -6) | 0 | Yes | Yes | Strong spatial consistency |
| HC > HE | 4 | 100 | <0.001 | (57, -3, -3) | 80 | 0.80 | (57, -3, -3) | 0 | Yes | Yes | Strong spatial consistency |
| HC > HE | 6 | 118 | <0.001 | (-30, -21, 3) | 98 | 0.83 | (-33, -18, 6) | 5.1962 | Yes | Yes | Strong spatial consistency |
| NHE > HC | 1 | 80 | 0.002 | (27, -12, 72) | 65 | 0.81 | (27, -12, 72) | 0 | Yes | Yes | Strong spatial consistency |

Note.—Strong spatial consistency required nonzero overlap, primary-cluster coverage ≥0.50, and nearest-peak distance ≤12 mm. TIV = total intracranial volume.

Supplementary Table 6. ALFF-to-GMV contrast-level TIV sensitivity.

| **Contrast** | **Primary significant voxels** | **Sensitivity significant voxels** | **Intersection voxels** | **Union voxels** | **Primary coverage** | **Sensitivity coverage** | **Dice** | **Jaccard** | **Direction consistent** | **Primary and sensitivity maps significant** | **Sensitivity-only cluster** | **Both maps null** |
| --- | --- | --- | --- | --- | --- | --- | --- | --- | --- | --- | --- | --- |
| Omnibus F test | 98 | 87 | 72 | 113 | 0.73 | 0.83 | 0.7784 | 0.6372 | Yes | Yes | No | No |
| HE > NHE | 0 | 0 | 0 | 0 |  |  |  |  | No | No | No | Yes |
| NHE > HE | 0 | 0 | 0 | 0 |  |  |  |  | No | No | No | Yes |
| HE > HC | 169 | 72 | 69 | 172 | 0.41 | 0.96 | 0.5726 | 0.4012 | Yes | Yes | No | No |
| HC > HE | 500 | 408 | 320 | 588 | 0.64 | 0.78 | 0.7048 | 0.5442 | Yes | Yes | Yes | No |
| NHE > HC | 0 | 0 | 0 | 0 |  |  |  |  | No | No | No | Yes |
| HC > NHE | 0 | 0 | 0 | 0 |  |  |  |  | No | No | No | Yes |

Note.—Coverage, Dice, and Jaccard values are descriptive spatial-comparison measures. TIV = total intracranial volume.

Supplementary Table 7. ALFF-to-GMV cluster-level TIV sensitivity.

| **Contrast** | **Cluster ID** | **Extent, voxels** | **Cluster FWE P** | **Primary peak MNI** | **Overlap, voxels** | **Primary coverage** | **Sensitivity peak MNI** | **Nearest-peak distance, mm** | **Overlaps sensitivity map** | **Distance ≤12 mm** | **TIV-adjusted spatial consistency** |
| --- | --- | --- | --- | --- | --- | --- | --- | --- | --- | --- | --- |
| Omnibus F test | 1 | 40 | <0.001 | (-21, -42, -3) | 27 | 0.68 | (-21, -42, -3) | 0 | Yes | Yes | Strong spatial consistency |
| Omnibus F test | 2 | 36 | <0.001 | (42, 42, 6) | 25 | 0.69 | (42, 42, 6) | 0 | Yes | Yes | Strong spatial consistency |
| Omnibus F test | 10 | 22 | 0.015 | (27, 57, 9) | 20 | 0.91 | (24, 57, 6) | 4.2426 | Yes | Yes | Strong spatial consistency |
| HE > HC | 1 | 120 | <0.001 | (-21, -42, -3) | 69 | 0.57 | (-21, -42, -3) | 0 | Yes | Yes | Strong spatial consistency |
| HE > HC | 4 | 49 | <0.001 | (27, -39, -6) | 0 | 0 | (-18, -36, -12) | 45.4973 | No | No | Spatially shifted |
| HC > HE | 1 | 111 | <0.001 | (42, 42, 6) | 102 | 0.92 | (42, 42, 6) | 0 | Yes | Yes | Strong spatial consistency |
| HC > HE | 2 | 50 | <0.001 | (48, 18, 27) | 42 | 0.84 | (48, 18, 27) | 0 | Yes | Yes | Strong spatial consistency |
| HC > HE | 3 | 45 | 0.001 | (48, -39, 48) | 38 | 0.84 | (48, -39, 48) | 0 | Yes | Yes | Strong spatial consistency |
| HC > HE | 4 | 142 | <0.001 | (0, -30, 45) | 83 | 0.58 | (0, -30, 45) | 0 | Yes | Yes | Strong spatial consistency |
| HC > HE | 5 | 45 | 0.001 | (-48, -72, 0) | 38 | 0.84 | (-48, -72, 0) | 0 | Yes | Yes | Strong spatial consistency |
| HC > HE | 12 | 34 | 0.007 | (-3, 27, 51) | 0 | 0 | (39, 33, 27) | 48.7442 | No | No | Spatially shifted |
| HC > HE | 13 | 32 | 0.010 | (48, -18, 39) | 0 | 0 | (48, -39, 48) | 22.8473 | No | No | Spatially shifted |
| HC > HE | 19 | 41 | 0.002 | (9, -63, 39) | 17 | 0.41 | (0, -51, 36) | 15.2971 | Yes | No | Partial spatial consistency |

Note.—Strong spatial consistency required nonzero overlap, primary-cluster coverage ≥0.50, and nearest-peak distance ≤12 mm. TIV = total intracranial volume.

Supplementary Table 8. All 48 exploratory ROI-clinical association tests.

| **ROI** | **Metric** | **Contrast** | **Anatomical label** | **Clinical variable** | **Clinical family** | **n** | **Partial Spearman ρ** | **P value** | **Family FDR q** | **Bootstrap 95% CI** | **TIV-adjusted ρ** | **TIV-adjusted P** | **TIV-adjusted FDR q** | **FDR significant** |
| --- | --- | --- | --- | --- | --- | --- | --- | --- | --- | --- | --- | --- | --- | --- |
| ALFF_HC>HE_C001 | ALFF-to-GMV | HC > HE | Right MFG middle frontal gyrus | Blood ammonia | Disease severity | 59 | 0.1174 | 0.398 | 0.718 | [-0.12, 0.34] | 0.1250 | 0.372 | 0.792 | No |
| ALFF_HC>HE_C001 | ALFF-to-GMV | HC > HE | Right MFG middle frontal gyrus | Child-Pugh score | Disease severity | 59 | -0.1588 | 0.251 | 0.718 | [-0.41, 0.12] | -0.2112 | 0.129 | 0.712 | No |
| ALFF_HC>HE_C001 | ALFF-to-GMV | HC > HE | Right MFG middle frontal gyrus | MELD score | Disease severity | 59 | 0.0730 | 0.600 | 0.800 | [-0.22, 0.37] | 0.0503 | 0.721 | 0.870 | No |
| ALFF_HC>HE_C001 | ALFF-to-GMV | HC > HE | Right MFG middle frontal gyrus | MMSE | Cognition | 59 | -0.0336 | 0.810 | 0.877 | [-0.29, 0.25] | -0.0354 | 0.801 | 0.855 | No |
| ALFF_HC>HE_C001 | ALFF-to-GMV | HC > HE | Right MFG middle frontal gyrus | MoCA | Cognition | 59 | -0.0883 | 0.525 | 0.788 | [-0.33, 0.19] | -0.0834 | 0.553 | 0.755 | No |
| ALFF_HC>HE_C001 | ALFF-to-GMV | HC > HE | Right MFG middle frontal gyrus | PHES | PHES Primary | 59 | -0.0753 | 0.589 | 0.937 | [-0.31, 0.19] | -0.0834 | 0.553 | 0.884 | No |
| ALFF_HE>HC_C001 | ALFF-to-GMV | HE > HC | Left Cerebral White Matter | Blood ammonia | Disease severity | 59 | 0.2925 | 0.032 | 0.501 | [-0.02, 0.56] | 0.3016 | 0.028 | 0.676 | No |
| ALFF_HE>HC_C001 | ALFF-to-GMV | HE > HC | Left Cerebral White Matter | Child-Pugh score | Disease severity | 59 | 0.1184 | 0.394 | 0.718 | [-0.15, 0.38] | 0.0918 | 0.513 | 0.821 | No |
| ALFF_HE>HC_C001 | ALFF-to-GMV | HE > HC | Left Cerebral White Matter | MELD score | Disease severity | 59 | -0.0090 | 0.949 | 0.975 | [-0.28, 0.29] | -0.0366 | 0.795 | 0.908 | No |
| ALFF_HE>HC_C001 | ALFF-to-GMV | HE > HC | Left Cerebral White Matter | MMSE | Cognition | 59 | 0.0788 | 0.571 | 0.788 | [-0.27, 0.41] | 0.0773 | 0.582 | 0.755 | No |
| ALFF_HE>HC_C001 | ALFF-to-GMV | HE > HC | Left Cerebral White Matter | MoCA | Cognition | 59 | 0.0651 | 0.640 | 0.788 | [-0.24, 0.34] | 0.0710 | 0.614 | 0.755 | No |
| ALFF_HE>HC_C001 | ALFF-to-GMV | HE > HC | Left Cerebral White Matter | PHES | PHES Primary | 59 | 0.0044 | 0.975 | 0.975 | [-0.32, 0.33] | -0.0033 | 0.981 | 0.981 | No |
| ReHo_HC>HE_C001 | ReHo-to-GMV | HC > HE | Left ACgG anterior cingulate gyrus | Blood ammonia | Disease severity | 59 | 0.0749 | 0.590 | 0.800 | [-0.21, 0.39] | 0.0627 | 0.656 | 0.870 | No |
| ReHo_HC>HE_C001 | ReHo-to-GMV | HC > HE | Left ACgG anterior cingulate gyrus | Child-Pugh score | Disease severity | 59 | -0.2603 | 0.057 | 0.501 | [-0.52, 0.03] | -0.2150 | 0.122 | 0.712 | No |
| ReHo_HC>HE_C001 | ReHo-to-GMV | HC > HE | Left ACgG anterior cingulate gyrus | MELD score | Disease severity | 59 | -0.1397 | 0.314 | 0.718 | [-0.43, 0.17] | -0.0962 | 0.493 | 0.821 | No |
| ReHo_HC>HE_C001 | ReHo-to-GMV | HC > HE | Left ACgG anterior cingulate gyrus | MMSE | Cognition | 59 | -0.1628 | 0.239 | 0.639 | [-0.40, 0.10] | -0.1617 | 0.247 | 0.660 | No |
| ReHo_HC>HE_C001 | ReHo-to-GMV | HC > HE | Left ACgG anterior cingulate gyrus | MoCA | Cognition | 59 | -0.2290 | 0.096 | 0.419 | [-0.49, 0.08] | -0.2433 | 0.079 | 0.427 | No |
| ReHo_HC>HE_C001 | ReHo-to-GMV | HC > HE | Left ACgG anterior cingulate gyrus | PHES | PHES Primary | 59 | -0.1191 | 0.391 | 0.809 | [-0.40, 0.18] | -0.1062 | 0.449 | 0.884 | No |
| ReHo_HC>HE_C002 | ReHo-to-GMV | HC > HE | Right Cerebral White Matter | Blood ammonia | Disease severity | 59 | -0.0941 | 0.499 | 0.748 | [-0.38, 0.21] | -0.0837 | 0.551 | 0.827 | No |
| ReHo_HC>HE_C002 | ReHo-to-GMV | HC > HE | Right Cerebral White Matter | Child-Pugh score | Disease severity | 59 | 0.0448 | 0.748 | 0.897 | [-0.24, 0.31] | -0.0134 | 0.924 | 0.924 | No |
| ReHo_HC>HE_C002 | ReHo-to-GMV | HC > HE | Right Cerebral White Matter | MELD score | Disease severity | 59 | -0.0044 | 0.975 | 0.975 | [-0.27, 0.26] | -0.0495 | 0.725 | 0.870 | No |
| ReHo_HC>HE_C002 | ReHo-to-GMV | HC > HE | Right Cerebral White Matter | MMSE | Cognition | 59 | -0.2735 | 0.045 | 0.419 | [-0.53, 0.03] | -0.2792 | 0.043 | 0.427 | No |
| ReHo_HC>HE_C002 | ReHo-to-GMV | HC > HE | Right Cerebral White Matter | MoCA | Cognition | 59 | -0.2337 | 0.089 | 0.419 | [-0.50, 0.06] | -0.2274 | 0.101 | 0.427 | No |
| ReHo_HC>HE_C002 | ReHo-to-GMV | HC > HE | Right Cerebral White Matter | PHES | PHES Primary | 59 | -0.1158 | 0.404 | 0.809 | [-0.40, 0.20] | -0.1303 | 0.352 | 0.884 | No |
| ReHo_HC>HE_C003 | ReHo-to-GMV | HC > HE | Right Cerebral White Matter | Blood ammonia | Disease severity | 59 | 0.1278 | 0.357 | 0.718 | [-0.18, 0.42] | 0.1190 | 0.396 | 0.792 | No |
| ReHo_HC>HE_C003 | ReHo-to-GMV | HC > HE | Right Cerebral White Matter | Child-Pugh score | Disease severity | 59 | -0.1877 | 0.174 | 0.718 | [-0.41, 0.06] | -0.1509 | 0.281 | 0.771 | No |
| ReHo_HC>HE_C003 | ReHo-to-GMV | HC > HE | Right Cerebral White Matter | MELD score | Disease severity | 59 | -0.2282 | 0.097 | 0.582 | [-0.46, 0.02] | -0.2013 | 0.148 | 0.712 | No |
| ReHo_HC>HE_C003 | ReHo-to-GMV | HC > HE | Right Cerebral White Matter | MMSE | Cognition | 59 | 0.0186 | 0.894 | 0.894 | [-0.25, 0.30] | 0.0214 | 0.879 | 0.879 | No |
| ReHo_HC>HE_C003 | ReHo-to-GMV | HC > HE | Right Cerebral White Matter | MoCA | Cognition | 59 | -0.0673 | 0.629 | 0.788 | [-0.34, 0.22] | -0.0761 | 0.588 | 0.755 | No |
| ReHo_HC>HE_C003 | ReHo-to-GMV | HC > HE | Right Cerebral White Matter | PHES | PHES Primary | 59 | -0.2785 | 0.041 | 0.332 | [-0.53, 0.02] | -0.2704 | 0.050 | 0.401 | No |
| ReHo_HC>HE_C004 | ReHo-to-GMV | HC > HE | Right Cerebral White Matter | Blood ammonia | Disease severity | 59 | -0.0965 | 0.488 | 0.748 | [-0.40, 0.26] | -0.0949 | 0.499 | 0.821 | No |
| ReHo_HC>HE_C004 | ReHo-to-GMV | HC > HE | Right Cerebral White Matter | Child-Pugh score | Disease severity | 59 | -0.1347 | 0.332 | 0.718 | [-0.36, 0.14] | -0.1573 | 0.261 | 0.771 | No |
| ReHo_HC>HE_C004 | ReHo-to-GMV | HC > HE | Right Cerebral White Matter | MELD score | Disease severity | 59 | -0.1123 | 0.419 | 0.718 | [-0.37, 0.15] | -0.1250 | 0.373 | 0.792 | No |
| ReHo_HC>HE_C004 | ReHo-to-GMV | HC > HE | Right Cerebral White Matter | MMSE | Cognition | 59 | -0.1006 | 0.469 | 0.788 | [-0.31, 0.14] | -0.1012 | 0.471 | 0.755 | No |
| ReHo_HC>HE_C004 | ReHo-to-GMV | HC > HE | Right Cerebral White Matter | MoCA | Cognition | 59 | -0.0729 | 0.600 | 0.788 | [-0.28, 0.16] | -0.0716 | 0.610 | 0.755 | No |
| ReHo_HC>HE_C004 | ReHo-to-GMV | HC > HE | Right Cerebral White Matter | PHES | PHES Primary | 59 | -0.1182 | 0.395 | 0.809 | [-0.37, 0.15] | -0.1208 | 0.389 | 0.884 | No |
| ReHo_HC>HE_C006 | ReHo-to-GMV | HC > HE | Left AIns anterior insula | Blood ammonia | Disease severity | 59 | -0.1316 | 0.343 | 0.718 | [-0.43, 0.23] | -0.1483 | 0.289 | 0.771 | No |
| ReHo_HC>HE_C006 | ReHo-to-GMV | HC > HE | Left AIns anterior insula | Child-Pugh score | Disease severity | 59 | -0.2551 | 0.063 | 0.501 | [-0.50, 0.02] | -0.2057 | 0.139 | 0.712 | No |
| ReHo_HC>HE_C006 | ReHo-to-GMV | HC > HE | Left AIns anterior insula | MELD score | Disease severity | 59 | -0.0330 | 0.813 | 0.929 | [-0.28, 0.24] | 0.0199 | 0.888 | 0.924 | No |
| ReHo_HC>HE_C006 | ReHo-to-GMV | HC > HE | Left AIns anterior insula | MMSE | Cognition | 59 | 0.0313 | 0.822 | 0.877 | [-0.29, 0.35] | 0.0354 | 0.801 | 0.855 | No |
| ReHo_HC>HE_C006 | ReHo-to-GMV | HC > HE | Left AIns anterior insula | MoCA | Cognition | 59 | -0.1158 | 0.404 | 0.788 | [-0.44, 0.19] | -0.1290 | 0.357 | 0.755 | No |
| ReHo_HC>HE_C006 | ReHo-to-GMV | HC > HE | Left AIns anterior insula | PHES | PHES Primary | 59 | -0.0531 | 0.703 | 0.937 | [-0.38, 0.26] | -0.0384 | 0.785 | 0.981 | No |
| ReHo_HE>HC_C001 | ReHo-to-GMV | HE > HC | Right Thalamus Proper | Blood ammonia | Disease severity | 59 | 0.0255 | 0.855 | 0.932 | [-0.25, 0.32] | 0.0258 | 0.854 | 0.924 | No |
| ReHo_HE>HC_C001 | ReHo-to-GMV | HE > HC | Right Thalamus Proper | Child-Pugh score | Disease severity | 59 | 0.0654 | 0.638 | 0.806 | [-0.23, 0.37] | 0.0703 | 0.617 | 0.870 | No |
| ReHo_HE>HC_C001 | ReHo-to-GMV | HE > HC | Right Thalamus Proper | MELD score | Disease severity | 59 | -0.1650 | 0.233 | 0.718 | [-0.45, 0.14] | -0.1737 | 0.213 | 0.771 | No |
| ReHo_HE>HC_C001 | ReHo-to-GMV | HE > HC | Right Thalamus Proper | MMSE | Cognition | 59 | 0.2131 | 0.122 | 0.419 | [-0.08, 0.46] | 0.2131 | 0.126 | 0.427 | No |
| ReHo_HE>HC_C001 | ReHo-to-GMV | HE > HC | Right Thalamus Proper | MoCA | Cognition | 59 | 0.2082 | 0.131 | 0.419 | [-0.11, 0.47] | 0.2088 | 0.134 | 0.427 | No |
| ReHo_HE>HC_C001 | ReHo-to-GMV | HE > HC | Right Thalamus Proper | PHES | PHES Primary | 59 | 0.0063 | 0.964 | 0.975 | [-0.33, 0.30] | 0.0061 | 0.966 | 0.981 | No |

Note.—Partial Spearman correlations were adjusted for group, sex, age, education, and mean framewise displacement. TIV-adjusted models additionally included ranked TIV. No association survived within-family FDR correction. CI = confidence interval; FDR = false discovery rate; ROI = region of interest.

Supplementary Table 9. Component-model cluster summary.

| **Model** | **Role** | **Covariates** | **Design rows** | **Design columns** | **Design rank** | **ERDF** | **Explicit mask voxels** | **Estimable voxels** | **Invariant-zero voxels** | **Status** |
| --- | --- | --- | --- | --- | --- | --- | --- | --- | --- | --- |
| szReHo | Post hoc component analysis | Age; Sex; Education; Mean Jenkinson FD | 97 | 7 | 7 | 90 | 42885 | 42885 | 0 | Completed |
| zALFF | Post hoc component analysis | Age; Sex; Education; Mean Jenkinson FD | 97 | 7 | 7 | 90 | 42885 | 42520 | 365 | Completed |
| GMV_A_NoTIV | post hoc diagnostic main analysis | Age; Sex; Education; Mean Jenkinson FD | 97 | 7 | 7 | 90 | 42885 | 42885 | 0 | Completed |
| GMV_B_TIV | post hoc TIV-adjusted sensitivity analysis | Age; Sex; Education; Mean Jenkinson FD; TIV mm3 | 97 | 8 | 8 | 89 | 42885 | 42885 | 0 | Completed |

Note.—GMV-A denotes the component model without TIV; GMV-B additionally includes TIV. ERDF = error degrees of freedom; FD = framewise displacement; GMV = gray matter volume; TIV = total intracranial volume.

Supplementary Table 10. Contrast-level ratio-component spatial comparisons.

| **Metric** | **Contrast** | **Ratio voxels** | **Numerator voxels** | **Opposite-direction GMV voxels** | **Ratio-numerator intersection** | **Ratio-numerator Dice** | **Ratio-numerator Jaccard** | **Ratio-opposite-GMV intersection** | **Ratio-opposite-GMV Dice** | **Ratio-opposite-GMV Jaccard** | **Descriptive class** |
| --- | --- | --- | --- | --- | --- | --- | --- | --- | --- | --- | --- |
| ReHo-to-GMV | Omnibus F test | 296 | 145 | 2289 | 131 | 0.5941 | 0.4226 | 70 | 0.0542 | 0.0278 | Joint numerator–denominator correspondence |
| ReHo-to-GMV | HE > HC | 65 | 0 | 1054 | 0 | 0 | 0 | 0 | 0 | 0 | Ratio-only spatial finding |
| ReHo-to-GMV | HC > HE | 647 | 434 | 1897 | 398 | 0.7364 | 0.5827 | 2 | 0.0016 | 0.0008 | Joint numerator–denominator correspondence |
| ReHo-to-GMV | HE > NHE | 0 | 0 | 0 | 0 | NA | NA | 0 | NA | NA | Indeterminate |
| ReHo-to-GMV | NHE > HE | 0 | 0 | 0 | 0 | NA | NA | 0 | NA | NA | Indeterminate |
| ReHo-to-GMV | NHE > HC | 80 | 0 | 647 | 0 | 0 | 0 | 0 | 0 | 0 | Ratio-only spatial finding |
| ReHo-to-GMV | HC > NHE | 0 | 0 | 212 | 0 | NA | NA | 0 | 0 | 0 | Indeterminate |
| ALFF-to-GMV | Omnibus F test | 98 | 173 | 2289 | 57 | 0.4207 | 0.2664 | 6 | 0.0050 | 0.0025 | Joint numerator–denominator correspondence |
| ALFF-to-GMV | HE > HC | 169 | 290 | 1054 | 60 | 0.2614 | 0.1504 | 0 | 0 | 0 | Numerator-dominant spatial correspondence |
| ALFF-to-GMV | HC > HE | 500 | 479 | 1897 | 355 | 0.7252 | 0.5689 | 29 | 0.0242 | 0.0122 | Joint numerator–denominator correspondence |
| ALFF-to-GMV | HE > NHE | 0 | 0 | 0 | 0 | NA | NA | 0 | NA | NA | Indeterminate |
| ALFF-to-GMV | NHE > HE | 0 | 0 | 0 | 0 | NA | NA | 0 | NA | NA | Indeterminate |
| ALFF-to-GMV | NHE > HC | 0 | 0 | 647 | 0 | NA | NA | 0 | 0 | 0 | Indeterminate |
| ALFF-to-GMV | HC > NHE | 0 | 0 | 212 | 0 | NA | NA | 0 | 0 | 0 | Indeterminate |

Note.—Spatial comparisons are threshold dependent and descriptive; they do not quantify causal component contributions. GMV = gray matter volume. NA = not applicable because one or both corrected component maps contained no corresponding cluster or peak.

Supplementary Table 11. Directional primary-cluster ratio-component spatial comparisons.

| **Metric** | **Contrast** | **Ratio cluster ID** | **Cluster voxels** | **Numerator overlap voxels** | **Numerator coverage, %** | **Opposite-GMV overlap voxels** | **Opposite-GMV coverage, %** | **Any-component overlap voxels** | **Ratio peak MNI** | **Nearest numerator peak, mm** | **Nearest opposite-GMV peak, mm** | **Descriptive class** |
| --- | --- | --- | --- | --- | --- | --- | --- | --- | --- | --- | --- | --- |
| ReHo-to-GMV | HC > HE | 1 | 99 | 91 | 91.9 | 2 | 2.0 | 93 | (0, 51, 6) | 0 | 16.4317 | Joint numerator–denominator correspondence |
| ReHo-to-GMV | HC > HE | 2 | 92 | 79 | 85.9 | 0 | 0.0 | 79 | (45, -12, 36) | 3 | 43.3705 | Numerator-dominant spatial correspondence |
| ReHo-to-GMV | HC > HE | 3 | 238 | 103 | 43.3 | 0 | 0.0 | 103 | (45, 12, -6) | 0 | 50.2892 | Numerator-dominant spatial correspondence |
| ReHo-to-GMV | HC > HE | 4 | 100 | 70 | 70.0 | 0 | 0.0 | 70 | (57, -3, -3) | 0 | 55.3986 | Numerator-dominant spatial correspondence |
| ReHo-to-GMV | HC > HE | 6 | 118 | 55 | 46.6 | 0 | 0.0 | 55 | (-30, -21, 3) | 10.8167 | 19.2094 | Numerator-dominant spatial correspondence |
| ReHo-to-GMV | HE > HC | 1 | 65 | 0 | 0.0 | 0 | 0.0 | 0 | (15, -18, 15) | NA | 21.2132 | Ratio-only spatial finding |
| ReHo-to-GMV | NHE > HC | 1 | 80 | 0 | 0.0 | 0 | 0.0 | 0 | (27, -12, 72) | NA | 63.6396 | Ratio-only spatial finding |
| ALFF-to-GMV | HC > HE | 1 | 111 | 94 | 84.7 | 29 | 26.1 | 105 | (42, 42, 6) | 0 | 19.4422 | Joint numerator–denominator correspondence |
| ALFF-to-GMV | HC > HE | 2 | 50 | 41 | 82.0 | 0 | 0.0 | 41 | (48, 18, 27) | 0 | 37.5899 | Numerator-dominant spatial correspondence |
| ALFF-to-GMV | HC > HE | 3 | 45 | 0 | 0.0 | 0 | 0.0 | 0 | (48, -39, 48) | 28.3019 | 53.2447 | Ratio-only spatial finding |
| ALFF-to-GMV | HC > HE | 4 | 142 | 108 | 76.1 | 0 | 0.0 | 108 | (0, -30, 45) | 3 | 31.8904 | Numerator-dominant spatial correspondence |
| ALFF-to-GMV | HC > HE | 5 | 45 | 40 | 88.9 | 0 | 0.0 | 40 | (-48, -72, 0) | 0 | 50.6458 | Numerator-dominant spatial correspondence |
| ALFF-to-GMV | HC > HE | 12 | 34 | 32 | 94.1 | 0 | 0.0 | 32 | (-3, 27, 51) | 0 | 53.1601 | Numerator-dominant spatial correspondence |
| ALFF-to-GMV | HC > HE | 13 | 32 | 0 | 0.0 | 0 | 0.0 | 0 | (48, -18, 39) | 37.9473 | 46.1844 | Ratio-only spatial finding |
| ALFF-to-GMV | HC > HE | 19 | 41 | 40 | 97.6 | 0 | 0.0 | 40 | (9, -63, 39) | 15.2971 | 45.793 | Numerator-dominant spatial correspondence |
| ALFF-to-GMV | HE > HC | 1 | 120 | 57 | 47.5 | 0 | 0.0 | 57 | (-21, -42, -3) | 0 | 27.8209 | Numerator-dominant spatial correspondence |
| ALFF-to-GMV | HE > HC | 4 | 49 | 3 | 6.1 | 0 | 0.0 | 3 | (27, -39, -6) | 18.2483 | 22.0454 | Numerator-dominant spatial correspondence |

Note.—This table is restricted to the 17 directional primary ratio clusters used in the directional component-overlap summary; omnibus component summaries are reported at the contrast level in Supplementary Table 10. Spatial comparisons are threshold dependent and descriptive. MNI = Montreal Neurological Institute; NA = not applicable because the corresponding corrected component map contained no cluster or peak.

Supplementary Table 12. Final-97-mask comparison quality control.

| **All-118 mask voxels** | **Final-97 mask voxels** | **Intersection voxels** | **Dice** | **Jaccard** | **Gained voxels** | **Lost voxels** | **Gained MNI minimum** | **Gained MNI maximum** | **Lost MNI minimum** | **Lost MNI maximum** |
| --- | --- | --- | --- | --- | --- | --- | --- | --- | --- | --- |
| 42885 | 42981 | 42883 | 0.9988 | 0.9977 | 98 | 2 | [-45.0 -60.0 0.0] | [42.0 66.0 51.0] | [-33.0 -21.0 39.0] | [9.0 0.0 75.0] |

Note.—The final-97 mask was reconstructed using the same thresholds as the all-118 mask.

Supplementary Table 13. Final-97-mask primary-cluster correspondence using canonical primary cluster IDs.

| **Metric** | **Contrast** | **Primary cluster** | **Primary cluster voxels** | **Sensitivity overlap voxels** | **Primary cluster coverage, %** | **Direction spatially retained** |
| --- | --- | --- | --- | --- | --- | --- |
| ReHo-to-GMV | Omnibus F test | ReHo_Omnibus_C001 | 31 | 31 | 100.0 | Yes |
| ReHo-to-GMV | Omnibus F test | ReHo_Omnibus_C002 | 40 | 40 | 100.0 | Yes |
| ReHo-to-GMV | Omnibus F test | ReHo_Omnibus_C003 | 35 | 35 | 100.0 | Yes |
| ReHo-to-GMV | Omnibus F test | ReHo_Omnibus_C004 | 77 | 77 | 100.0 | Yes |
| ReHo-to-GMV | Omnibus F test | ReHo_Omnibus_C006 | 38 | 38 | 100.0 | Yes |
| ReHo-to-GMV | Omnibus F test | ReHo_Omnibus_C007 | 75 | 75 | 100.0 | Yes |
| ReHo-to-GMV | HE > HC | ReHo_HE>HC_C001 | 65 | 65 | 100.0 | Yes |
| ReHo-to-GMV | HC > HE | ReHo_HC>HE_C001 | 99 | 99 | 100.0 | Yes |
| ReHo-to-GMV | HC > HE | ReHo_HC>HE_C002 | 92 | 92 | 100.0 | Yes |
| ReHo-to-GMV | HC > HE | ReHo_HC>HE_C003 | 238 | 238 | 100.0 | Yes |
| ReHo-to-GMV | HC > HE | ReHo_HC>HE_C004 | 100 | 100 | 100.0 | Yes |
| ReHo-to-GMV | HC > HE | ReHo_HC>HE_C006 | 118 | 118 | 100.0 | Yes |
| ReHo-to-GMV | NHE > HC | ReHo_NHE>HC_C001 | 80 | 80 | 100.0 | Yes |
| ALFF-to-GMV | Omnibus F test | ALFF_Omnibus_C001 | 40 | 40 | 100.0 | Yes |
| ALFF-to-GMV | Omnibus F test | ALFF_Omnibus_C002 | 36 | 36 | 100.0 | Yes |
| ALFF-to-GMV | Omnibus F test | ALFF_Omnibus_C010 | 22 | 22 | 100.0 | Yes |
| ALFF-to-GMV | HE > HC | ALFF_HE>HC_C001 | 120 | 120 | 100.0 | Yes |
| ALFF-to-GMV | HE > HC | ALFF_HE>HC_C004 | 49 | 49 | 100.0 | Yes |
| ALFF-to-GMV | HC > HE | ALFF_HC>HE_C001 | 111 | 111 | 100.0 | Yes |
| ALFF-to-GMV | HC > HE | ALFF_HC>HE_C002 | 50 | 50 | 100.0 | Yes |
| ALFF-to-GMV | HC > HE | ALFF_HC>HE_C005 | 45 | 45 | 100.0 | Yes |
| ALFF-to-GMV | HC > HE | ALFF_HC>HE_C004 | 142 | 142 | 100.0 | Yes |
| ALFF-to-GMV | HC > HE | ALFF_HC>HE_C005 | 45 | 45 | 100.0 | Yes |
| ALFF-to-GMV | HC > HE | ALFF_HC>HE_C012 | 34 | 34 | 100.0 | Yes |
| ALFF-to-GMV | HC > HE | ALFF_HC>HE_C013 | 32 | 32 | 100.0 | Yes |
| ALFF-to-GMV | HC > HE | ALFF_HC>HE_C019 | 41 | 41 | 100.0 | Yes |

Note.—Direction spatially retained indicates same-direction overlap between the original and final-97-mask primary clusters. Canonical cluster IDs follow Supplementary Table 3.

Supplementary Table 14. Expanded-motion model diagnostics.

| **Design rank** | **Design columns** | **Scaled condition number** | **Reciprocal condition number (XᵀX)** | **Maximum VIF** | **Precheck passed** |
| --- | --- | --- | --- | --- | --- |
| 9 | 9 | 2.6251 | 1.222e-05 | 1.8773 | Yes |

Note.—VIF = variance-inflation factor.

Supplementary Table 15. Expanded-motion variance-inflation factors.

| **Covariate** | **VIF** |
| --- | --- |
| Age | 1.4967 |
| Sex | 1.2324 |
| Education | 1.5672 |
| Mean Jenkinson FD | 1.7616 |
| Maximum translation | 1.8773 |
| Maximum rotation | 1.6635 |

Note.—FD = framewise displacement; VIF = variance-inflation factor.

Supplementary Table 16. Expanded-motion primary-cluster correspondence using canonical primary cluster IDs.

| **Metric** | **Contrast** | **Primary cluster** | **Primary cluster voxels** | **Sensitivity overlap voxels** | **Primary cluster coverage, %** | **Direction spatially retained** |
| --- | --- | --- | --- | --- | --- | --- |
| ReHo-to-GMV | Omnibus F test | ReHo_Omnibus_C001 | 31 | 0 | 0.0 | No |
| ReHo-to-GMV | Omnibus F test | ReHo_Omnibus_C002 | 40 | 37 | 92.5 | Yes |
| ReHo-to-GMV | Omnibus F test | ReHo_Omnibus_C003 | 35 | 33 | 94.3 | Yes |
| ReHo-to-GMV | Omnibus F test | ReHo_Omnibus_C004 | 77 | 63 | 81.8 | Yes |
| ReHo-to-GMV | Omnibus F test | ReHo_Omnibus_C006 | 38 | 37 | 97.4 | Yes |
| ReHo-to-GMV | Omnibus F test | ReHo_Omnibus_C007 | 75 | 33 | 44.0 | Yes |
| ReHo-to-GMV | HE > HC | ReHo_HE>HC_C001 | 65 | 62 | 95.4 | Yes |
| ReHo-to-GMV | HC > HE | ReHo_HC>HE_C001 | 99 | 90 | 90.9 | Yes |
| ReHo-to-GMV | HC > HE | ReHo_HC>HE_C002 | 92 | 71 | 77.2 | Yes |
| ReHo-to-GMV | HC > HE | ReHo_HC>HE_C003 | 238 | 218 | 91.6 | Yes |
| ReHo-to-GMV | HC > HE | ReHo_HC>HE_C004 | 100 | 92 | 92.0 | Yes |
| ReHo-to-GMV | HC > HE | ReHo_HC>HE_C006 | 118 | 102 | 86.4 | Yes |
| ReHo-to-GMV | NHE > HC | ReHo_NHE>HC_C001 | 80 | 68 | 85.0 | Yes |
| ALFF-to-GMV | Omnibus F test | ALFF_Omnibus_C001 | 40 | 36 | 90.0 | Yes |
| ALFF-to-GMV | Omnibus F test | ALFF_Omnibus_C002 | 36 | 28 | 77.8 | Yes |
| ALFF-to-GMV | Omnibus F test | ALFF_Omnibus_C010 | 22 | 20 | 90.9 | Yes |
| ALFF-to-GMV | HE > HC | ALFF_HE>HC_C001 | 120 | 106 | 88.3 | Yes |
| ALFF-to-GMV | HE > HC | ALFF_HE>HC_C004 | 49 | 45 | 91.8 | Yes |
| ALFF-to-GMV | HC > HE | ALFF_HC>HE_C001 | 111 | 108 | 97.3 | Yes |
| ALFF-to-GMV | HC > HE | ALFF_HC>HE_C002 | 50 | 47 | 94.0 | Yes |
| ALFF-to-GMV | HC > HE | ALFF_HC>HE_C005 | 45 | 41 | 91.1 | Yes |
| ALFF-to-GMV | HC > HE | ALFF_HC>HE_C004 | 142 | 132 | 93.0 | Yes |
| ALFF-to-GMV | HC > HE | ALFF_HC>HE_C005 | 45 | 40 | 88.9 | Yes |
| ALFF-to-GMV | HC > HE | ALFF_HC>HE_C012 | 34 | 0 | 0.0 | No |
| ALFF-to-GMV | HC > HE | ALFF_HC>HE_C013 | 32 | 0 | 0.0 | No |
| ALFF-to-GMV | HC > HE | ALFF_HC>HE_C019 | 41 | 34 | 82.9 | Yes |

Note.—Direction spatially retained indicates same-direction overlap between the primary and expanded-motion models. Canonical cluster IDs follow Supplementary Table 3.

Supplementary Table 17. Same-sample adjusted component estimates with HC3 confidence intervals.

| **Ratio metric** | **Source contrast** | **Ratio cluster ID** | **Cluster voxels** | **Measure** | **Group** | **N** | **Adjusted mean** | **95% CI lower** | **95% CI upper** | **CI method** | **Selection caveat** |
| --- | --- | --- | --- | --- | --- | --- | --- | --- | --- | --- | --- |
| ReHo-to-GMV | HC > HE | 1 | 99 | ReHo-to-GMV | HC | 38 | 1.7854 | 1.42 | 2.15 | HC3 descriptive CI | Same-sample selected cluster; winner’s curse possible |
| ReHo-to-GMV | HC > HE | 1 | 99 | ReHo-to-GMV | NHE | 29 | 0.7791 | 0.42 | 1.14 | HC3 descriptive CI | Same-sample selected cluster; winner’s curse possible |
| ReHo-to-GMV | HC > HE | 1 | 99 | ReHo-to-GMV | HE | 30 | 0.2716 | -0.14 | 0.69 | HC3 descriptive CI | Same-sample selected cluster; winner’s curse possible |
| ReHo-to-GMV | HC > HE | 1 | 99 | ReHo | HC | 38 | 0.6247 | 0.49 | 0.76 | HC3 descriptive CI | Same-sample selected cluster; winner’s curse possible |
| ReHo-to-GMV | HC > HE | 1 | 99 | ReHo | NHE | 29 | 0.2633 | 0.13 | 0.40 | HC3 descriptive CI | Same-sample selected cluster; winner’s curse possible |
| ReHo-to-GMV | HC > HE | 1 | 99 | ReHo | HE | 30 | 0.0727 | -0.09 | 0.23 | HC3 descriptive CI | Same-sample selected cluster; winner’s curse possible |
| ReHo-to-GMV | HC > HE | 1 | 99 | GMV-A | HC | 38 | 0.4039 | 0.39 | 0.42 | HC3 descriptive CI | Same-sample selected cluster; winner’s curse possible |
| ReHo-to-GMV | HC > HE | 1 | 99 | GMV-A | NHE | 29 | 0.4102 | 0.39 | 0.43 | HC3 descriptive CI | Same-sample selected cluster; winner’s curse possible |
| ReHo-to-GMV | HC > HE | 1 | 99 | GMV-A | HE | 30 | 0.4159 | 0.40 | 0.44 | HC3 descriptive CI | Same-sample selected cluster; winner’s curse possible |
| ReHo-to-GMV | HC > HE | 1 | 99 | GMV-B | HC | 38 | 0.4139 | 0.40 | 0.43 | HC3 descriptive CI | Same-sample selected cluster; winner’s curse possible |
| ReHo-to-GMV | HC > HE | 1 | 99 | GMV-B | NHE | 29 | 0.4080 | 0.39 | 0.43 | HC3 descriptive CI | Same-sample selected cluster; winner’s curse possible |
| ReHo-to-GMV | HC > HE | 1 | 99 | GMV-B | HE | 30 | 0.4054 | 0.39 | 0.42 | HC3 descriptive CI | Same-sample selected cluster; winner’s curse possible |
| ReHo-to-GMV | HC > HE | 2 | 92 | ReHo-to-GMV | HC | 38 | 1.2661 | 0.82 | 1.71 | HC3 descriptive CI | Same-sample selected cluster; winner’s curse possible |
| ReHo-to-GMV | HC > HE | 2 | 92 | ReHo-to-GMV | NHE | 29 | 0.5369 | 0.12 | 0.95 | HC3 descriptive CI | Same-sample selected cluster; winner’s curse possible |
| ReHo-to-GMV | HC > HE | 2 | 92 | ReHo-to-GMV | HE | 30 | -0.6113 | -1.10 | -0.13 | HC3 descriptive CI | Same-sample selected cluster; winner’s curse possible |
| ReHo-to-GMV | HC > HE | 2 | 92 | ReHo | HC | 38 | 0.4277 | 0.30 | 0.56 | HC3 descriptive CI | Same-sample selected cluster; winner’s curse possible |
| ReHo-to-GMV | HC > HE | 2 | 92 | ReHo | NHE | 29 | 0.2070 | 0.08 | 0.33 | HC3 descriptive CI | Same-sample selected cluster; winner’s curse possible |
| ReHo-to-GMV | HC > HE | 2 | 92 | ReHo | HE | 30 | -0.0872 | -0.21 | 0.04 | HC3 descriptive CI | Same-sample selected cluster; winner’s curse possible |
| ReHo-to-GMV | HC > HE | 2 | 92 | GMV-A | HC | 38 | 0.3533 | 0.34 | 0.37 | HC3 descriptive CI | Same-sample selected cluster; winner’s curse possible |
| ReHo-to-GMV | HC > HE | 2 | 92 | GMV-A | NHE | 29 | 0.3172 | 0.30 | 0.33 | HC3 descriptive CI | Same-sample selected cluster; winner’s curse possible |
| ReHo-to-GMV | HC > HE | 2 | 92 | GMV-A | HE | 30 | 0.3089 | 0.29 | 0.33 | HC3 descriptive CI | Same-sample selected cluster; winner’s curse possible |
| ReHo-to-GMV | HC > HE | 2 | 92 | GMV-B | HC | 38 | 0.3555 | 0.34 | 0.37 | HC3 descriptive CI | Same-sample selected cluster; winner’s curse possible |
| ReHo-to-GMV | HC > HE | 2 | 92 | GMV-B | NHE | 29 | 0.3167 | 0.30 | 0.33 | HC3 descriptive CI | Same-sample selected cluster; winner’s curse possible |
| ReHo-to-GMV | HC > HE | 2 | 92 | GMV-B | HE | 30 | 0.3066 | 0.29 | 0.33 | HC3 descriptive CI | Same-sample selected cluster; winner’s curse possible |
| ReHo-to-GMV | HC > HE | 3 | 238 | ReHo-to-GMV | HC | 38 | -0.3660 | -0.57 | -0.16 | HC3 descriptive CI | Same-sample selected cluster; winner’s curse possible |
| ReHo-to-GMV | HC > HE | 3 | 238 | ReHo-to-GMV | NHE | 29 | -1.0040 | -1.24 | -0.77 | HC3 descriptive CI | Same-sample selected cluster; winner’s curse possible |
| ReHo-to-GMV | HC > HE | 3 | 238 | ReHo-to-GMV | HE | 30 | -1.3603 | -1.57 | -1.15 | HC3 descriptive CI | Same-sample selected cluster; winner’s curse possible |
| ReHo-to-GMV | HC > HE | 3 | 238 | ReHo | HC | 38 | -0.1567 | -0.24 | -0.07 | HC3 descriptive CI | Same-sample selected cluster; winner’s curse possible |
| ReHo-to-GMV | HC > HE | 3 | 238 | ReHo | NHE | 29 | -0.3813 | -0.47 | -0.29 | HC3 descriptive CI | Same-sample selected cluster; winner’s curse possible |
| ReHo-to-GMV | HC > HE | 3 | 238 | ReHo | HE | 30 | -0.4969 | -0.57 | -0.43 | HC3 descriptive CI | Same-sample selected cluster; winner’s curse possible |
| ReHo-to-GMV | HC > HE | 3 | 238 | GMV-A | HC | 38 | 0.5120 | 0.50 | 0.53 | HC3 descriptive CI | Same-sample selected cluster; winner’s curse possible |
| ReHo-to-GMV | HC > HE | 3 | 238 | GMV-A | NHE | 29 | 0.4741 | 0.46 | 0.49 | HC3 descriptive CI | Same-sample selected cluster; winner’s curse possible |
| ReHo-to-GMV | HC > HE | 3 | 238 | GMV-A | HE | 30 | 0.4601 | 0.44 | 0.48 | HC3 descriptive CI | Same-sample selected cluster; winner’s curse possible |
| ReHo-to-GMV | HC > HE | 3 | 238 | GMV-B | HC | 38 | 0.5183 | 0.51 | 0.53 | HC3 descriptive CI | Same-sample selected cluster; winner’s curse possible |
| ReHo-to-GMV | HC > HE | 3 | 238 | GMV-B | NHE | 29 | 0.4727 | 0.46 | 0.49 | HC3 descriptive CI | Same-sample selected cluster; winner’s curse possible |
| ReHo-to-GMV | HC > HE | 3 | 238 | GMV-B | HE | 30 | 0.4536 | 0.43 | 0.47 | HC3 descriptive CI | Same-sample selected cluster; winner’s curse possible |
| ReHo-to-GMV | HC > HE | 4 | 100 | ReHo-to-GMV | HC | 38 | 1.0323 | 0.73 | 1.33 | HC3 descriptive CI | Same-sample selected cluster; winner’s curse possible |
| ReHo-to-GMV | HC > HE | 4 | 100 | ReHo-to-GMV | NHE | 29 | 0.2746 | -0.10 | 0.65 | HC3 descriptive CI | Same-sample selected cluster; winner’s curse possible |
| ReHo-to-GMV | HC > HE | 4 | 100 | ReHo-to-GMV | HE | 30 | -0.3518 | -0.55 | -0.15 | HC3 descriptive CI | Same-sample selected cluster; winner’s curse possible |
| ReHo-to-GMV | HC > HE | 4 | 100 | ReHo | HC | 38 | 0.3825 | 0.27 | 0.49 | HC3 descriptive CI | Same-sample selected cluster; winner’s curse possible |
| ReHo-to-GMV | HC > HE | 4 | 100 | ReHo | NHE | 29 | 0.1156 | -0.03 | 0.26 | HC3 descriptive CI | Same-sample selected cluster; winner’s curse possible |
| ReHo-to-GMV | HC > HE | 4 | 100 | ReHo | HE | 30 | -0.1251 | -0.20 | -0.05 | HC3 descriptive CI | Same-sample selected cluster; winner’s curse possible |
| ReHo-to-GMV | HC > HE | 4 | 100 | GMV-A | HC | 38 | 0.3996 | 0.38 | 0.42 | HC3 descriptive CI | Same-sample selected cluster; winner’s curse possible |
| ReHo-to-GMV | HC > HE | 4 | 100 | GMV-A | NHE | 29 | 0.3930 | 0.37 | 0.41 | HC3 descriptive CI | Same-sample selected cluster; winner’s curse possible |
| ReHo-to-GMV | HC > HE | 4 | 100 | GMV-A | HE | 30 | 0.3944 | 0.37 | 0.41 | HC3 descriptive CI | Same-sample selected cluster; winner’s curse possible |
| ReHo-to-GMV | HC > HE | 4 | 100 | GMV-B | HC | 38 | 0.4062 | 0.39 | 0.42 | HC3 descriptive CI | Same-sample selected cluster; winner’s curse possible |
| ReHo-to-GMV | HC > HE | 4 | 100 | GMV-B | NHE | 29 | 0.3915 | 0.37 | 0.41 | HC3 descriptive CI | Same-sample selected cluster; winner’s curse possible |
| ReHo-to-GMV | HC > HE | 4 | 100 | GMV-B | HE | 30 | 0.3874 | 0.37 | 0.40 | HC3 descriptive CI | Same-sample selected cluster; winner’s curse possible |
| ReHo-to-GMV | HC > HE | 6 | 118 | ReHo-to-GMV | HC | 38 | -0.4205 | -0.64 | -0.20 | HC3 descriptive CI | Same-sample selected cluster; winner’s curse possible |
| ReHo-to-GMV | HC > HE | 6 | 118 | ReHo-to-GMV | NHE | 29 | -1.3028 | -1.51 | -1.10 | HC3 descriptive CI | Same-sample selected cluster; winner’s curse possible |
| ReHo-to-GMV | HC > HE | 6 | 118 | ReHo-to-GMV | HE | 30 | -1.5617 | -1.81 | -1.32 | HC3 descriptive CI | Same-sample selected cluster; winner’s curse possible |
| ReHo-to-GMV | HC > HE | 6 | 118 | ReHo | HC | 38 | -0.1436 | -0.23 | -0.06 | HC3 descriptive CI | Same-sample selected cluster; winner’s curse possible |
| ReHo-to-GMV | HC > HE | 6 | 118 | ReHo | NHE | 29 | -0.4289 | -0.51 | -0.35 | HC3 descriptive CI | Same-sample selected cluster; winner’s curse possible |
| ReHo-to-GMV | HC > HE | 6 | 118 | ReHo | HE | 30 | -0.5083 | -0.59 | -0.43 | HC3 descriptive CI | Same-sample selected cluster; winner’s curse possible |
| ReHo-to-GMV | HC > HE | 6 | 118 | GMV-A | HC | 38 | 0.4361 | 0.42 | 0.45 | HC3 descriptive CI | Same-sample selected cluster; winner’s curse possible |
| ReHo-to-GMV | HC > HE | 6 | 118 | GMV-A | NHE | 29 | 0.4129 | 0.40 | 0.43 | HC3 descriptive CI | Same-sample selected cluster; winner’s curse possible |
| ReHo-to-GMV | HC > HE | 6 | 118 | GMV-A | HE | 30 | 0.4070 | 0.39 | 0.42 | HC3 descriptive CI | Same-sample selected cluster; winner’s curse possible |
| ReHo-to-GMV | HC > HE | 6 | 118 | GMV-B | HC | 38 | 0.4420 | 0.43 | 0.45 | HC3 descriptive CI | Same-sample selected cluster; winner’s curse possible |
| ReHo-to-GMV | HC > HE | 6 | 118 | GMV-B | NHE | 29 | 0.4116 | 0.40 | 0.43 | HC3 descriptive CI | Same-sample selected cluster; winner’s curse possible |
| ReHo-to-GMV | HC > HE | 6 | 118 | GMV-B | HE | 30 | 0.4008 | 0.39 | 0.41 | HC3 descriptive CI | Same-sample selected cluster; winner’s curse possible |
| ReHo-to-GMV | HE > HC | 1 | 65 | ReHo-to-GMV | HC | 38 | -1.9338 | -2.15 | -1.72 | HC3 descriptive CI | Same-sample selected cluster; winner’s curse possible |
| ReHo-to-GMV | HE > HC | 1 | 65 | ReHo-to-GMV | NHE | 29 | -1.1808 | -1.50 | -0.86 | HC3 descriptive CI | Same-sample selected cluster; winner’s curse possible |
| ReHo-to-GMV | HE > HC | 1 | 65 | ReHo-to-GMV | HE | 30 | -0.5825 | -0.95 | -0.21 | HC3 descriptive CI | Same-sample selected cluster; winner’s curse possible |
| ReHo-to-GMV | HE > HC | 1 | 65 | ReHo | HC | 38 | -0.5358 | -0.61 | -0.46 | HC3 descriptive CI | Same-sample selected cluster; winner’s curse possible |
| ReHo-to-GMV | HE > HC | 1 | 65 | ReHo | NHE | 29 | -0.3668 | -0.48 | -0.26 | HC3 descriptive CI | Same-sample selected cluster; winner’s curse possible |
| ReHo-to-GMV | HE > HC | 1 | 65 | ReHo | HE | 30 | -0.2143 | -0.36 | -0.07 | HC3 descriptive CI | Same-sample selected cluster; winner’s curse possible |
| ReHo-to-GMV | HE > HC | 1 | 65 | GMV-A | HC | 38 | 0.3206 | 0.30 | 0.34 | HC3 descriptive CI | Same-sample selected cluster; winner’s curse possible |
| ReHo-to-GMV | HE > HC | 1 | 65 | GMV-A | NHE | 29 | 0.3903 | 0.36 | 0.42 | HC3 descriptive CI | Same-sample selected cluster; winner’s curse possible |
| ReHo-to-GMV | HE > HC | 1 | 65 | GMV-A | HE | 30 | 0.4404 | 0.41 | 0.47 | HC3 descriptive CI | Same-sample selected cluster; winner’s curse possible |
| ReHo-to-GMV | HE > HC | 1 | 65 | GMV-B | HC | 38 | 0.3256 | 0.31 | 0.34 | HC3 descriptive CI | Same-sample selected cluster; winner’s curse possible |
| ReHo-to-GMV | HE > HC | 1 | 65 | GMV-B | NHE | 29 | 0.3892 | 0.36 | 0.42 | HC3 descriptive CI | Same-sample selected cluster; winner’s curse possible |
| ReHo-to-GMV | HE > HC | 1 | 65 | GMV-B | HE | 30 | 0.4352 | 0.41 | 0.46 | HC3 descriptive CI | Same-sample selected cluster; winner’s curse possible |
| ReHo-to-GMV | NHE > HC | 1 | 80 | ReHo-to-GMV | HC | 38 | -1.3829 | -1.94 | -0.83 | HC3 descriptive CI | Same-sample selected cluster; winner’s curse possible |
| ReHo-to-GMV | NHE > HC | 1 | 80 | ReHo-to-GMV | NHE | 29 | 1.2646 | 0.42 | 2.11 | HC3 descriptive CI | Same-sample selected cluster; winner’s curse possible |
| ReHo-to-GMV | NHE > HC | 1 | 80 | ReHo-to-GMV | HE | 30 | -0.0643 | -0.70 | 0.57 | HC3 descriptive CI | Same-sample selected cluster; winner’s curse possible |
| ReHo-to-GMV | NHE > HC | 1 | 80 | ReHo | HC | 38 | -0.3579 | -0.50 | -0.22 | HC3 descriptive CI | Same-sample selected cluster; winner’s curse possible |
| ReHo-to-GMV | NHE > HC | 1 | 80 | ReHo | NHE | 29 | 0.2682 | 0.08 | 0.46 | HC3 descriptive CI | Same-sample selected cluster; winner’s curse possible |
| ReHo-to-GMV | NHE > HC | 1 | 80 | ReHo | HE | 30 | 0.0086 | -0.16 | 0.18 | HC3 descriptive CI | Same-sample selected cluster; winner’s curse possible |
| ReHo-to-GMV | NHE > HC | 1 | 80 | GMV-A | HC | 38 | 0.2784 | 0.27 | 0.29 | HC3 descriptive CI | Same-sample selected cluster; winner’s curse possible |
| ReHo-to-GMV | NHE > HC | 1 | 80 | GMV-A | NHE | 29 | 0.2701 | 0.26 | 0.28 | HC3 descriptive CI | Same-sample selected cluster; winner’s curse possible |
| ReHo-to-GMV | NHE > HC | 1 | 80 | GMV-A | HE | 30 | 0.2904 | 0.27 | 0.31 | HC3 descriptive CI | Same-sample selected cluster; winner’s curse possible |
| ReHo-to-GMV | NHE > HC | 1 | 80 | GMV-B | HC | 38 | 0.2836 | 0.27 | 0.29 | HC3 descriptive CI | Same-sample selected cluster; winner’s curse possible |
| ReHo-to-GMV | NHE > HC | 1 | 80 | GMV-B | NHE | 29 | 0.2690 | 0.26 | 0.28 | HC3 descriptive CI | Same-sample selected cluster; winner’s curse possible |
| ReHo-to-GMV | NHE > HC | 1 | 80 | GMV-B | HE | 30 | 0.2849 | 0.27 | 0.30 | HC3 descriptive CI | Same-sample selected cluster; winner’s curse possible |
| ReHo-to-GMV | Omnibus F test | 1 | 31 | ReHo-to-GMV | HC | 38 | -1.8745 | -2.08 | -1.67 | HC3 descriptive CI | Same-sample selected cluster; winner’s curse possible |
| ReHo-to-GMV | Omnibus F test | 1 | 31 | ReHo-to-GMV | NHE | 29 | -1.0237 | -1.40 | -0.65 | HC3 descriptive CI | Same-sample selected cluster; winner’s curse possible |
| ReHo-to-GMV | Omnibus F test | 1 | 31 | ReHo-to-GMV | HE | 30 | -0.6268 | -0.93 | -0.32 | HC3 descriptive CI | Same-sample selected cluster; winner’s curse possible |
| ReHo-to-GMV | Omnibus F test | 1 | 31 | ReHo | HC | 38 | -0.5879 | -0.67 | -0.51 | HC3 descriptive CI | Same-sample selected cluster; winner’s curse possible |
| ReHo-to-GMV | Omnibus F test | 1 | 31 | ReHo | NHE | 29 | -0.3827 | -0.52 | -0.24 | HC3 descriptive CI | Same-sample selected cluster; winner’s curse possible |
| ReHo-to-GMV | Omnibus F test | 1 | 31 | ReHo | HE | 30 | -0.2808 | -0.43 | -0.13 | HC3 descriptive CI | Same-sample selected cluster; winner’s curse possible |
| ReHo-to-GMV | Omnibus F test | 1 | 31 | GMV-A | HC | 38 | 0.3484 | 0.33 | 0.37 | HC3 descriptive CI | Same-sample selected cluster; winner’s curse possible |
| ReHo-to-GMV | Omnibus F test | 1 | 31 | GMV-A | NHE | 29 | 0.4349 | 0.40 | 0.47 | HC3 descriptive CI | Same-sample selected cluster; winner’s curse possible |
| ReHo-to-GMV | Omnibus F test | 1 | 31 | GMV-A | HE | 30 | 0.4942 | 0.46 | 0.53 | HC3 descriptive CI | Same-sample selected cluster; winner’s curse possible |
| ReHo-to-GMV | Omnibus F test | 1 | 31 | GMV-B | HC | 38 | 0.3537 | 0.33 | 0.37 | HC3 descriptive CI | Same-sample selected cluster; winner’s curse possible |
| ReHo-to-GMV | Omnibus F test | 1 | 31 | GMV-B | NHE | 29 | 0.4338 | 0.40 | 0.47 | HC3 descriptive CI | Same-sample selected cluster; winner’s curse possible |
| ReHo-to-GMV | Omnibus F test | 1 | 31 | GMV-B | HE | 30 | 0.4886 | 0.46 | 0.52 | HC3 descriptive CI | Same-sample selected cluster; winner’s curse possible |
| ReHo-to-GMV | Omnibus F test | 2 | 40 | ReHo-to-GMV | HC | 38 | 2.3131 | 1.86 | 2.76 | HC3 descriptive CI | Same-sample selected cluster; winner’s curse possible |
| ReHo-to-GMV | Omnibus F test | 2 | 40 | ReHo-to-GMV | NHE | 29 | 0.9882 | 0.53 | 1.44 | HC3 descriptive CI | Same-sample selected cluster; winner’s curse possible |
| ReHo-to-GMV | Omnibus F test | 2 | 40 | ReHo-to-GMV | HE | 30 | 0.4743 | -0.04 | 0.99 | HC3 descriptive CI | Same-sample selected cluster; winner’s curse possible |
| ReHo-to-GMV | Omnibus F test | 2 | 40 | ReHo | HC | 38 | 0.7537 | 0.61 | 0.90 | HC3 descriptive CI | Same-sample selected cluster; winner’s curse possible |
| ReHo-to-GMV | Omnibus F test | 2 | 40 | ReHo | NHE | 29 | 0.3119 | 0.16 | 0.47 | HC3 descriptive CI | Same-sample selected cluster; winner’s curse possible |
| ReHo-to-GMV | Omnibus F test | 2 | 40 | ReHo | HE | 30 | 0.1422 | -0.04 | 0.33 | HC3 descriptive CI | Same-sample selected cluster; winner’s curse possible |
| ReHo-to-GMV | Omnibus F test | 2 | 40 | GMV-A | HC | 38 | 0.3656 | 0.35 | 0.38 | HC3 descriptive CI | Same-sample selected cluster; winner’s curse possible |
| ReHo-to-GMV | Omnibus F test | 2 | 40 | GMV-A | NHE | 29 | 0.3807 | 0.36 | 0.40 | HC3 descriptive CI | Same-sample selected cluster; winner’s curse possible |
| ReHo-to-GMV | Omnibus F test | 2 | 40 | GMV-A | HE | 30 | 0.3891 | 0.37 | 0.41 | HC3 descriptive CI | Same-sample selected cluster; winner’s curse possible |
| ReHo-to-GMV | Omnibus F test | 2 | 40 | GMV-B | HC | 38 | 0.3756 | 0.36 | 0.39 | HC3 descriptive CI | Same-sample selected cluster; winner’s curse possible |
| ReHo-to-GMV | Omnibus F test | 2 | 40 | GMV-B | NHE | 29 | 0.3785 | 0.36 | 0.39 | HC3 descriptive CI | Same-sample selected cluster; winner’s curse possible |
| ReHo-to-GMV | Omnibus F test | 2 | 40 | GMV-B | HE | 30 | 0.3785 | 0.36 | 0.39 | HC3 descriptive CI | Same-sample selected cluster; winner’s curse possible |
| ReHo-to-GMV | Omnibus F test | 3 | 35 | ReHo-to-GMV | HC | 38 | 1.3746 | 0.81 | 1.94 | HC3 descriptive CI | Same-sample selected cluster; winner’s curse possible |
| ReHo-to-GMV | Omnibus F test | 3 | 35 | ReHo-to-GMV | NHE | 29 | 0.2924 | -0.21 | 0.79 | HC3 descriptive CI | Same-sample selected cluster; winner’s curse possible |
| ReHo-to-GMV | Omnibus F test | 3 | 35 | ReHo-to-GMV | HE | 30 | -0.9035 | -1.53 | -0.28 | HC3 descriptive CI | Same-sample selected cluster; winner’s curse possible |
| ReHo-to-GMV | Omnibus F test | 3 | 35 | ReHo | HC | 38 | 0.4047 | 0.24 | 0.56 | HC3 descriptive CI | Same-sample selected cluster; winner’s curse possible |
| ReHo-to-GMV | Omnibus F test | 3 | 35 | ReHo | NHE | 29 | 0.1133 | -0.03 | 0.25 | HC3 descriptive CI | Same-sample selected cluster; winner’s curse possible |
| ReHo-to-GMV | Omnibus F test | 3 | 35 | ReHo | HE | 30 | -0.1593 | -0.30 | -0.02 | HC3 descriptive CI | Same-sample selected cluster; winner’s curse possible |
| ReHo-to-GMV | Omnibus F test | 3 | 35 | GMV-A | HC | 38 | 0.3104 | 0.30 | 0.33 | HC3 descriptive CI | Same-sample selected cluster; winner’s curse possible |
| ReHo-to-GMV | Omnibus F test | 3 | 35 | GMV-A | NHE | 29 | 0.2814 | 0.26 | 0.30 | HC3 descriptive CI | Same-sample selected cluster; winner’s curse possible |
| ReHo-to-GMV | Omnibus F test | 3 | 35 | GMV-A | HE | 30 | 0.2751 | 0.25 | 0.30 | HC3 descriptive CI | Same-sample selected cluster; winner’s curse possible |
| ReHo-to-GMV | Omnibus F test | 3 | 35 | GMV-B | HC | 38 | 0.3127 | 0.30 | 0.33 | HC3 descriptive CI | Same-sample selected cluster; winner’s curse possible |
| ReHo-to-GMV | Omnibus F test | 3 | 35 | GMV-B | NHE | 29 | 0.2809 | 0.27 | 0.30 | HC3 descriptive CI | Same-sample selected cluster; winner’s curse possible |
| ReHo-to-GMV | Omnibus F test | 3 | 35 | GMV-B | HE | 30 | 0.2727 | 0.25 | 0.29 | HC3 descriptive CI | Same-sample selected cluster; winner’s curse possible |
| ReHo-to-GMV | Omnibus F test | 4 | 77 | ReHo-to-GMV | HC | 38 | 0.3383 | 0.08 | 0.60 | HC3 descriptive CI | Same-sample selected cluster; winner’s curse possible |
| ReHo-to-GMV | Omnibus F test | 4 | 77 | ReHo-to-GMV | NHE | 29 | -0.4734 | -0.69 | -0.25 | HC3 descriptive CI | Same-sample selected cluster; winner’s curse possible |
| ReHo-to-GMV | Omnibus F test | 4 | 77 | ReHo-to-GMV | HE | 30 | -0.7267 | -0.97 | -0.49 | HC3 descriptive CI | Same-sample selected cluster; winner’s curse possible |
| ReHo-to-GMV | Omnibus F test | 4 | 77 | ReHo | HC | 38 | 0.1320 | 0.02 | 0.24 | HC3 descriptive CI | Same-sample selected cluster; winner’s curse possible |
| ReHo-to-GMV | Omnibus F test | 4 | 77 | ReHo | NHE | 29 | -0.2099 | -0.30 | -0.12 | HC3 descriptive CI | Same-sample selected cluster; winner’s curse possible |
| ReHo-to-GMV | Omnibus F test | 4 | 77 | ReHo | HE | 30 | -0.3251 | -0.43 | -0.22 | HC3 descriptive CI | Same-sample selected cluster; winner’s curse possible |
| ReHo-to-GMV | Omnibus F test | 4 | 77 | GMV-A | HC | 38 | 0.5115 | 0.49 | 0.53 | HC3 descriptive CI | Same-sample selected cluster; winner’s curse possible |
| ReHo-to-GMV | Omnibus F test | 4 | 77 | GMV-A | NHE | 29 | 0.4863 | 0.47 | 0.51 | HC3 descriptive CI | Same-sample selected cluster; winner’s curse possible |
| ReHo-to-GMV | Omnibus F test | 4 | 77 | GMV-A | HE | 30 | 0.4884 | 0.47 | 0.51 | HC3 descriptive CI | Same-sample selected cluster; winner’s curse possible |
| ReHo-to-GMV | Omnibus F test | 4 | 77 | GMV-B | HC | 38 | 0.5191 | 0.51 | 0.53 | HC3 descriptive CI | Same-sample selected cluster; winner’s curse possible |
| ReHo-to-GMV | Omnibus F test | 4 | 77 | GMV-B | NHE | 29 | 0.4846 | 0.47 | 0.50 | HC3 descriptive CI | Same-sample selected cluster; winner’s curse possible |
| ReHo-to-GMV | Omnibus F test | 4 | 77 | GMV-B | HE | 30 | 0.4804 | 0.46 | 0.50 | HC3 descriptive CI | Same-sample selected cluster; winner’s curse possible |
| ReHo-to-GMV | Omnibus F test | 5 | 38 | ReHo-to-GMV | HC | 38 | 1.1797 | 0.84 | 1.52 | HC3 descriptive CI | Same-sample selected cluster; winner’s curse possible |
| ReHo-to-GMV | Omnibus F test | 5 | 38 | ReHo-to-GMV | NHE | 29 | 0.2633 | -0.18 | 0.70 | HC3 descriptive CI | Same-sample selected cluster; winner’s curse possible |
| ReHo-to-GMV | Omnibus F test | 5 | 38 | ReHo-to-GMV | HE | 30 | -0.2977 | -0.54 | -0.06 | HC3 descriptive CI | Same-sample selected cluster; winner’s curse possible |
| ReHo-to-GMV | Omnibus F test | 5 | 38 | ReHo | HC | 38 | 0.4713 | 0.34 | 0.60 | HC3 descriptive CI | Same-sample selected cluster; winner’s curse possible |
| ReHo-to-GMV | Omnibus F test | 5 | 38 | ReHo | NHE | 29 | 0.1349 | -0.04 | 0.31 | HC3 descriptive CI | Same-sample selected cluster; winner’s curse possible |
| ReHo-to-GMV | Omnibus F test | 5 | 38 | ReHo | HE | 30 | -0.0992 | -0.19 | -0.01 | HC3 descriptive CI | Same-sample selected cluster; winner’s curse possible |
| ReHo-to-GMV | Omnibus F test | 5 | 38 | GMV-A | HC | 38 | 0.4049 | 0.39 | 0.42 | HC3 descriptive CI | Same-sample selected cluster; winner’s curse possible |
| ReHo-to-GMV | Omnibus F test | 5 | 38 | GMV-A | NHE | 29 | 0.4033 | 0.38 | 0.42 | HC3 descriptive CI | Same-sample selected cluster; winner’s curse possible |
| ReHo-to-GMV | Omnibus F test | 5 | 38 | GMV-A | HE | 30 | 0.4051 | 0.38 | 0.43 | HC3 descriptive CI | Same-sample selected cluster; winner’s curse possible |
| ReHo-to-GMV | Omnibus F test | 5 | 38 | GMV-B | HC | 38 | 0.4119 | 0.39 | 0.43 | HC3 descriptive CI | Same-sample selected cluster; winner’s curse possible |
| ReHo-to-GMV | Omnibus F test | 5 | 38 | GMV-B | NHE | 29 | 0.4018 | 0.38 | 0.42 | HC3 descriptive CI | Same-sample selected cluster; winner’s curse possible |
| ReHo-to-GMV | Omnibus F test | 5 | 38 | GMV-B | HE | 30 | 0.3977 | 0.38 | 0.42 | HC3 descriptive CI | Same-sample selected cluster; winner’s curse possible |
| ReHo-to-GMV | Omnibus F test | 6 | 75 | ReHo-to-GMV | HC | 38 | -0.4481 | -0.68 | -0.22 | HC3 descriptive CI | Same-sample selected cluster; winner’s curse possible |
| ReHo-to-GMV | Omnibus F test | 6 | 75 | ReHo-to-GMV | NHE | 29 | -1.4772 | -1.70 | -1.25 | HC3 descriptive CI | Same-sample selected cluster; winner’s curse possible |
| ReHo-to-GMV | Omnibus F test | 6 | 75 | ReHo-to-GMV | HE | 30 | -1.6136 | -1.87 | -1.36 | HC3 descriptive CI | Same-sample selected cluster; winner’s curse possible |
| ReHo-to-GMV | Omnibus F test | 6 | 75 | ReHo | HC | 38 | -0.1362 | -0.22 | -0.05 | HC3 descriptive CI | Same-sample selected cluster; winner’s curse possible |
| ReHo-to-GMV | Omnibus F test | 6 | 75 | ReHo | NHE | 29 | -0.4726 | -0.55 | -0.39 | HC3 descriptive CI | Same-sample selected cluster; winner’s curse possible |
| ReHo-to-GMV | Omnibus F test | 6 | 75 | ReHo | HE | 30 | -0.5027 | -0.59 | -0.42 | HC3 descriptive CI | Same-sample selected cluster; winner’s curse possible |
| ReHo-to-GMV | Omnibus F test | 6 | 75 | GMV-A | HC | 38 | 0.4395 | 0.43 | 0.45 | HC3 descriptive CI | Same-sample selected cluster; winner’s curse possible |
| ReHo-to-GMV | Omnibus F test | 6 | 75 | GMV-A | NHE | 29 | 0.4166 | 0.40 | 0.43 | HC3 descriptive CI | Same-sample selected cluster; winner’s curse possible |
| ReHo-to-GMV | Omnibus F test | 6 | 75 | GMV-A | HE | 30 | 0.4117 | 0.40 | 0.43 | HC3 descriptive CI | Same-sample selected cluster; winner’s curse possible |
| ReHo-to-GMV | Omnibus F test | 6 | 75 | GMV-B | HC | 38 | 0.4456 | 0.44 | 0.46 | HC3 descriptive CI | Same-sample selected cluster; winner’s curse possible |
| ReHo-to-GMV | Omnibus F test | 6 | 75 | GMV-B | NHE | 29 | 0.4153 | 0.40 | 0.43 | HC3 descriptive CI | Same-sample selected cluster; winner’s curse possible |
| ReHo-to-GMV | Omnibus F test | 6 | 75 | GMV-B | HE | 30 | 0.4053 | 0.39 | 0.42 | HC3 descriptive CI | Same-sample selected cluster; winner’s curse possible |
| ALFF-to-GMV | HC > HE | 1 | 111 | ALFF-to-GMV | HC | 38 | 1.2631 | 0.90 | 1.62 | HC3 descriptive CI | Same-sample selected cluster; winner’s curse possible |
| ALFF-to-GMV | HC > HE | 1 | 111 | ALFF-to-GMV | NHE | 29 | 0.3445 | 0.09 | 0.60 | HC3 descriptive CI | Same-sample selected cluster; winner’s curse possible |
| ALFF-to-GMV | HC > HE | 1 | 111 | ALFF-to-GMV | HE | 30 | -0.2610 | -0.45 | -0.07 | HC3 descriptive CI | Same-sample selected cluster; winner’s curse possible |
| ALFF-to-GMV | HC > HE | 1 | 111 | ALFF | HC | 38 | 0.4721 | 0.34 | 0.60 | HC3 descriptive CI | Same-sample selected cluster; winner’s curse possible |
| ALFF-to-GMV | HC > HE | 1 | 111 | ALFF | NHE | 29 | 0.1246 | 0.02 | 0.22 | HC3 descriptive CI | Same-sample selected cluster; winner’s curse possible |
| ALFF-to-GMV | HC > HE | 1 | 111 | ALFF | HE | 30 | -0.0888 | -0.17 | -0.01 | HC3 descriptive CI | Same-sample selected cluster; winner’s curse possible |
| ALFF-to-GMV | HC > HE | 1 | 111 | GMV-A | HC | 38 | 0.4156 | 0.40 | 0.44 | HC3 descriptive CI | Same-sample selected cluster; winner’s curse possible |
| ALFF-to-GMV | HC > HE | 1 | 111 | GMV-A | NHE | 29 | 0.4199 | 0.39 | 0.45 | HC3 descriptive CI | Same-sample selected cluster; winner’s curse possible |
| ALFF-to-GMV | HC > HE | 1 | 111 | GMV-A | HE | 30 | 0.4429 | 0.42 | 0.46 | HC3 descriptive CI | Same-sample selected cluster; winner’s curse possible |
| ALFF-to-GMV | HC > HE | 1 | 111 | GMV-B | HC | 38 | 0.4251 | 0.41 | 0.44 | HC3 descriptive CI | Same-sample selected cluster; winner’s curse possible |
| ALFF-to-GMV | HC > HE | 1 | 111 | GMV-B | NHE | 29 | 0.4178 | 0.39 | 0.44 | HC3 descriptive CI | Same-sample selected cluster; winner’s curse possible |
| ALFF-to-GMV | HC > HE | 1 | 111 | GMV-B | HE | 30 | 0.4329 | 0.42 | 0.45 | HC3 descriptive CI | Same-sample selected cluster; winner’s curse possible |
| ALFF-to-GMV | HC > HE | 2 | 50 | ALFF-to-GMV | HC | 38 | 0.8886 | 0.61 | 1.17 | HC3 descriptive CI | Same-sample selected cluster; winner’s curse possible |
| ALFF-to-GMV | HC > HE | 2 | 50 | ALFF-to-GMV | NHE | 29 | 0.1613 | -0.11 | 0.43 | HC3 descriptive CI | Same-sample selected cluster; winner’s curse possible |
| ALFF-to-GMV | HC > HE | 2 | 50 | ALFF-to-GMV | HE | 30 | -0.2682 | -0.42 | -0.12 | HC3 descriptive CI | Same-sample selected cluster; winner’s curse possible |
| ALFF-to-GMV | HC > HE | 2 | 50 | ALFF | HC | 38 | 0.3906 | 0.27 | 0.51 | HC3 descriptive CI | Same-sample selected cluster; winner’s curse possible |
| ALFF-to-GMV | HC > HE | 2 | 50 | ALFF | NHE | 29 | 0.0781 | -0.04 | 0.20 | HC3 descriptive CI | Same-sample selected cluster; winner’s curse possible |
| ALFF-to-GMV | HC > HE | 2 | 50 | ALFF | HE | 30 | -0.1081 | -0.17 | -0.04 | HC3 descriptive CI | Same-sample selected cluster; winner’s curse possible |
| ALFF-to-GMV | HC > HE | 2 | 50 | GMV-A | HC | 38 | 0.4576 | 0.44 | 0.48 | HC3 descriptive CI | Same-sample selected cluster; winner’s curse possible |
| ALFF-to-GMV | HC > HE | 2 | 50 | GMV-A | NHE | 29 | 0.4408 | 0.42 | 0.46 | HC3 descriptive CI | Same-sample selected cluster; winner’s curse possible |
| ALFF-to-GMV | HC > HE | 2 | 50 | GMV-A | HE | 30 | 0.4590 | 0.44 | 0.48 | HC3 descriptive CI | Same-sample selected cluster; winner’s curse possible |
| ALFF-to-GMV | HC > HE | 2 | 50 | GMV-B | HC | 38 | 0.4669 | 0.45 | 0.48 | HC3 descriptive CI | Same-sample selected cluster; winner’s curse possible |
| ALFF-to-GMV | HC > HE | 2 | 50 | GMV-B | NHE | 29 | 0.4387 | 0.42 | 0.46 | HC3 descriptive CI | Same-sample selected cluster; winner’s curse possible |
| ALFF-to-GMV | HC > HE | 2 | 50 | GMV-B | HE | 30 | 0.4492 | 0.43 | 0.47 | HC3 descriptive CI | Same-sample selected cluster; winner’s curse possible |
| ALFF-to-GMV | HC > HE | 3 | 45 | ALFF-to-GMV | HC | 38 | 0.6559 | 0.38 | 0.93 | HC3 descriptive CI | Same-sample selected cluster; winner’s curse possible |
| ALFF-to-GMV | HC > HE | 3 | 45 | ALFF-to-GMV | NHE | 29 | -0.1311 | -0.39 | 0.13 | HC3 descriptive CI | Same-sample selected cluster; winner’s curse possible |
| ALFF-to-GMV | HC > HE | 3 | 45 | ALFF-to-GMV | HE | 30 | -0.4983 | -0.72 | -0.28 | HC3 descriptive CI | Same-sample selected cluster; winner’s curse possible |
| ALFF-to-GMV | HC > HE | 3 | 45 | ALFF | HC | 38 | 0.2909 | 0.16 | 0.42 | HC3 descriptive CI | Same-sample selected cluster; winner’s curse possible |
| ALFF-to-GMV | HC > HE | 3 | 45 | ALFF | NHE | 29 | -0.0099 | -0.13 | 0.11 | HC3 descriptive CI | Same-sample selected cluster; winner’s curse possible |
| ALFF-to-GMV | HC > HE | 3 | 45 | ALFF | HE | 30 | -0.1659 | -0.25 | -0.08 | HC3 descriptive CI | Same-sample selected cluster; winner’s curse possible |
| ALFF-to-GMV | HC > HE | 3 | 45 | GMV-A | HC | 38 | 0.4558 | 0.43 | 0.48 | HC3 descriptive CI | Same-sample selected cluster; winner’s curse possible |
| ALFF-to-GMV | HC > HE | 3 | 45 | GMV-A | NHE | 29 | 0.4058 | 0.38 | 0.43 | HC3 descriptive CI | Same-sample selected cluster; winner’s curse possible |
| ALFF-to-GMV | HC > HE | 3 | 45 | GMV-A | HE | 30 | 0.4013 | 0.38 | 0.43 | HC3 descriptive CI | Same-sample selected cluster; winner’s curse possible |
| ALFF-to-GMV | HC > HE | 3 | 45 | GMV-B | HC | 38 | 0.4588 | 0.44 | 0.48 | HC3 descriptive CI | Same-sample selected cluster; winner’s curse possible |
| ALFF-to-GMV | HC > HE | 3 | 45 | GMV-B | NHE | 29 | 0.4051 | 0.38 | 0.43 | HC3 descriptive CI | Same-sample selected cluster; winner’s curse possible |
| ALFF-to-GMV | HC > HE | 3 | 45 | GMV-B | HE | 30 | 0.3981 | 0.37 | 0.42 | HC3 descriptive CI | Same-sample selected cluster; winner’s curse possible |
| ALFF-to-GMV | HC > HE | 4 | 142 | ALFF-to-GMV | HC | 38 | 2.2086 | 1.88 | 2.54 | HC3 descriptive CI | Same-sample selected cluster; winner’s curse possible |
| ALFF-to-GMV | HC > HE | 4 | 142 | ALFF-to-GMV | NHE | 29 | 1.0374 | 0.64 | 1.43 | HC3 descriptive CI | Same-sample selected cluster; winner’s curse possible |
| ALFF-to-GMV | HC > HE | 4 | 142 | ALFF-to-GMV | HE | 30 | 0.3668 | 0.09 | 0.65 | HC3 descriptive CI | Same-sample selected cluster; winner’s curse possible |
| ALFF-to-GMV | HC > HE | 4 | 142 | ALFF | HC | 38 | 0.7859 | 0.67 | 0.90 | HC3 descriptive CI | Same-sample selected cluster; winner’s curse possible |
| ALFF-to-GMV | HC > HE | 4 | 142 | ALFF | NHE | 29 | 0.3589 | 0.21 | 0.50 | HC3 descriptive CI | Same-sample selected cluster; winner’s curse possible |
| ALFF-to-GMV | HC > HE | 4 | 142 | ALFF | HE | 30 | 0.1235 | 0.02 | 0.23 | HC3 descriptive CI | Same-sample selected cluster; winner’s curse possible |
| ALFF-to-GMV | HC > HE | 4 | 142 | GMV-A | HC | 38 | 0.4357 | 0.42 | 0.45 | HC3 descriptive CI | Same-sample selected cluster; winner’s curse possible |
| ALFF-to-GMV | HC > HE | 4 | 142 | GMV-A | NHE | 29 | 0.4251 | 0.40 | 0.45 | HC3 descriptive CI | Same-sample selected cluster; winner’s curse possible |
| ALFF-to-GMV | HC > HE | 4 | 142 | GMV-A | HE | 30 | 0.4467 | 0.43 | 0.47 | HC3 descriptive CI | Same-sample selected cluster; winner’s curse possible |
| ALFF-to-GMV | HC > HE | 4 | 142 | GMV-B | HC | 38 | 0.4441 | 0.43 | 0.46 | HC3 descriptive CI | Same-sample selected cluster; winner’s curse possible |
| ALFF-to-GMV | HC > HE | 4 | 142 | GMV-B | NHE | 29 | 0.4233 | 0.41 | 0.44 | HC3 descriptive CI | Same-sample selected cluster; winner’s curse possible |
| ALFF-to-GMV | HC > HE | 4 | 142 | GMV-B | HE | 30 | 0.4379 | 0.42 | 0.45 | HC3 descriptive CI | Same-sample selected cluster; winner’s curse possible |
| ALFF-to-GMV | HC > HE | 5 | 45 | ALFF-to-GMV | HC | 38 | 1.3784 | 0.99 | 1.77 | HC3 descriptive CI | Same-sample selected cluster; winner’s curse possible |
| ALFF-to-GMV | HC > HE | 5 | 45 | ALFF-to-GMV | NHE | 29 | 0.4310 | 0.10 | 0.76 | HC3 descriptive CI | Same-sample selected cluster; winner’s curse possible |
| ALFF-to-GMV | HC > HE | 5 | 45 | ALFF-to-GMV | HE | 30 | -0.0546 | -0.30 | 0.19 | HC3 descriptive CI | Same-sample selected cluster; winner’s curse possible |
| ALFF-to-GMV | HC > HE | 5 | 45 | ALFF | HC | 38 | 0.6525 | 0.47 | 0.83 | HC3 descriptive CI | Same-sample selected cluster; winner’s curse possible |
| ALFF-to-GMV | HC > HE | 5 | 45 | ALFF | NHE | 29 | 0.2210 | 0.05 | 0.39 | HC3 descriptive CI | Same-sample selected cluster; winner’s curse possible |
| ALFF-to-GMV | HC > HE | 5 | 45 | ALFF | HE | 30 | -0.0399 | -0.16 | 0.08 | HC3 descriptive CI | Same-sample selected cluster; winner’s curse possible |
| ALFF-to-GMV | HC > HE | 5 | 45 | GMV-A | HC | 38 | 0.5047 | 0.48 | 0.53 | HC3 descriptive CI | Same-sample selected cluster; winner’s curse possible |
| ALFF-to-GMV | HC > HE | 5 | 45 | GMV-A | NHE | 29 | 0.4919 | 0.47 | 0.52 | HC3 descriptive CI | Same-sample selected cluster; winner’s curse possible |
| ALFF-to-GMV | HC > HE | 5 | 45 | GMV-A | HE | 30 | 0.5158 | 0.49 | 0.54 | HC3 descriptive CI | Same-sample selected cluster; winner’s curse possible |
| ALFF-to-GMV | HC > HE | 5 | 45 | GMV-B | HC | 38 | 0.5133 | 0.49 | 0.53 | HC3 descriptive CI | Same-sample selected cluster; winner’s curse possible |
| ALFF-to-GMV | HC > HE | 5 | 45 | GMV-B | NHE | 29 | 0.4900 | 0.46 | 0.52 | HC3 descriptive CI | Same-sample selected cluster; winner’s curse possible |
| ALFF-to-GMV | HC > HE | 5 | 45 | GMV-B | HE | 30 | 0.5067 | 0.48 | 0.53 | HC3 descriptive CI | Same-sample selected cluster; winner’s curse possible |
| ALFF-to-GMV | HC > HE | 12 | 34 | ALFF-to-GMV | HC | 38 | 3.0858 | 2.39 | 3.78 | HC3 descriptive CI | Same-sample selected cluster; winner’s curse possible |
| ALFF-to-GMV | HC > HE | 12 | 34 | ALFF-to-GMV | NHE | 29 | 1.1747 | 0.65 | 1.70 | HC3 descriptive CI | Same-sample selected cluster; winner’s curse possible |
| ALFF-to-GMV | HC > HE | 12 | 34 | ALFF-to-GMV | HE | 30 | 0.7438 | 0.15 | 1.34 | HC3 descriptive CI | Same-sample selected cluster; winner’s curse possible |
| ALFF-to-GMV | HC > HE | 12 | 34 | ALFF | HC | 38 | 0.8395 | 0.65 | 1.03 | HC3 descriptive CI | Same-sample selected cluster; winner’s curse possible |
| ALFF-to-GMV | HC > HE | 12 | 34 | ALFF | NHE | 29 | 0.3279 | 0.18 | 0.48 | HC3 descriptive CI | Same-sample selected cluster; winner’s curse possible |
| ALFF-to-GMV | HC > HE | 12 | 34 | ALFF | HE | 30 | 0.1882 | 0.03 | 0.34 | HC3 descriptive CI | Same-sample selected cluster; winner’s curse possible |
| ALFF-to-GMV | HC > HE | 12 | 34 | GMV-A | HC | 38 | 0.2974 | 0.28 | 0.31 | HC3 descriptive CI | Same-sample selected cluster; winner’s curse possible |
| ALFF-to-GMV | HC > HE | 12 | 34 | GMV-A | NHE | 29 | 0.2914 | 0.28 | 0.30 | HC3 descriptive CI | Same-sample selected cluster; winner’s curse possible |
| ALFF-to-GMV | HC > HE | 12 | 34 | GMV-A | HE | 30 | 0.3013 | 0.29 | 0.32 | HC3 descriptive CI | Same-sample selected cluster; winner’s curse possible |
| ALFF-to-GMV | HC > HE | 12 | 34 | GMV-B | HC | 38 | 0.3031 | 0.29 | 0.32 | HC3 descriptive CI | Same-sample selected cluster; winner’s curse possible |
| ALFF-to-GMV | HC > HE | 12 | 34 | GMV-B | NHE | 29 | 0.2901 | 0.28 | 0.30 | HC3 descriptive CI | Same-sample selected cluster; winner’s curse possible |
| ALFF-to-GMV | HC > HE | 12 | 34 | GMV-B | HE | 30 | 0.2953 | 0.28 | 0.31 | HC3 descriptive CI | Same-sample selected cluster; winner’s curse possible |
| ALFF-to-GMV | HC > HE | 13 | 32 | ALFF-to-GMV | HC | 38 | -0.2104 | -0.49 | 0.07 | HC3 descriptive CI | Same-sample selected cluster; winner’s curse possible |
| ALFF-to-GMV | HC > HE | 13 | 32 | ALFF-to-GMV | NHE | 29 | -1.0961 | -1.36 | -0.83 | HC3 descriptive CI | Same-sample selected cluster; winner’s curse possible |
| ALFF-to-GMV | HC > HE | 13 | 32 | ALFF-to-GMV | HE | 30 | -1.3180 | -1.56 | -1.08 | HC3 descriptive CI | Same-sample selected cluster; winner’s curse possible |
| ALFF-to-GMV | HC > HE | 13 | 32 | ALFF | HC | 38 | -0.0711 | -0.16 | 0.02 | HC3 descriptive CI | Same-sample selected cluster; winner’s curse possible |
| ALFF-to-GMV | HC > HE | 13 | 32 | ALFF | NHE | 29 | -0.3067 | -0.39 | -0.23 | HC3 descriptive CI | Same-sample selected cluster; winner’s curse possible |
| ALFF-to-GMV | HC > HE | 13 | 32 | ALFF | HE | 30 | -0.3620 | -0.43 | -0.29 | HC3 descriptive CI | Same-sample selected cluster; winner’s curse possible |
| ALFF-to-GMV | HC > HE | 13 | 32 | GMV-A | HC | 38 | 0.3418 | 0.33 | 0.36 | HC3 descriptive CI | Same-sample selected cluster; winner’s curse possible |
| ALFF-to-GMV | HC > HE | 13 | 32 | GMV-A | NHE | 29 | 0.3090 | 0.29 | 0.32 | HC3 descriptive CI | Same-sample selected cluster; winner’s curse possible |
| ALFF-to-GMV | HC > HE | 13 | 32 | GMV-A | HE | 30 | 0.3058 | 0.28 | 0.33 | HC3 descriptive CI | Same-sample selected cluster; winner’s curse possible |
| ALFF-to-GMV | HC > HE | 13 | 32 | GMV-B | HC | 38 | 0.3449 | 0.33 | 0.36 | HC3 descriptive CI | Same-sample selected cluster; winner’s curse possible |
| ALFF-to-GMV | HC > HE | 13 | 32 | GMV-B | NHE | 29 | 0.3084 | 0.29 | 0.32 | HC3 descriptive CI | Same-sample selected cluster; winner’s curse possible |
| ALFF-to-GMV | HC > HE | 13 | 32 | GMV-B | HE | 30 | 0.3026 | 0.28 | 0.32 | HC3 descriptive CI | Same-sample selected cluster; winner’s curse possible |
| ALFF-to-GMV | HC > HE | 19 | 41 | ALFF-to-GMV | HC | 38 | 1.9966 | 1.51 | 2.49 | HC3 descriptive CI | Same-sample selected cluster; winner’s curse possible |
| ALFF-to-GMV | HC > HE | 19 | 41 | ALFF-to-GMV | NHE | 29 | 1.2055 | 0.67 | 1.74 | HC3 descriptive CI | Same-sample selected cluster; winner’s curse possible |
| ALFF-to-GMV | HC > HE | 19 | 41 | ALFF-to-GMV | HE | 30 | 0.2451 | -0.06 | 0.55 | HC3 descriptive CI | Same-sample selected cluster; winner’s curse possible |
| ALFF-to-GMV | HC > HE | 19 | 41 | ALFF | HC | 38 | 0.8617 | 0.66 | 1.07 | HC3 descriptive CI | Same-sample selected cluster; winner’s curse possible |
| ALFF-to-GMV | HC > HE | 19 | 41 | ALFF | NHE | 29 | 0.5297 | 0.30 | 0.76 | HC3 descriptive CI | Same-sample selected cluster; winner’s curse possible |
| ALFF-to-GMV | HC > HE | 19 | 41 | ALFF | HE | 30 | 0.1424 | 0.03 | 0.26 | HC3 descriptive CI | Same-sample selected cluster; winner’s curse possible |
| ALFF-to-GMV | HC > HE | 19 | 41 | GMV-A | HC | 38 | 0.4295 | 0.41 | 0.45 | HC3 descriptive CI | Same-sample selected cluster; winner’s curse possible |
| ALFF-to-GMV | HC > HE | 19 | 41 | GMV-A | NHE | 29 | 0.4084 | 0.39 | 0.43 | HC3 descriptive CI | Same-sample selected cluster; winner’s curse possible |
| ALFF-to-GMV | HC > HE | 19 | 41 | GMV-A | HE | 30 | 0.4112 | 0.39 | 0.43 | HC3 descriptive CI | Same-sample selected cluster; winner’s curse possible |
| ALFF-to-GMV | HC > HE | 19 | 41 | GMV-B | HC | 38 | 0.4369 | 0.42 | 0.45 | HC3 descriptive CI | Same-sample selected cluster; winner’s curse possible |
| ALFF-to-GMV | HC > HE | 19 | 41 | GMV-B | NHE | 29 | 0.4068 | 0.39 | 0.42 | HC3 descriptive CI | Same-sample selected cluster; winner’s curse possible |
| ALFF-to-GMV | HC > HE | 19 | 41 | GMV-B | HE | 30 | 0.4035 | 0.39 | 0.42 | HC3 descriptive CI | Same-sample selected cluster; winner’s curse possible |
| ALFF-to-GMV | HE > HC | 1 | 120 | ALFF-to-GMV | HC | 38 | -1.4329 | -1.51 | -1.36 | HC3 descriptive CI | Same-sample selected cluster; winner’s curse possible |
| ALFF-to-GMV | HE > HC | 1 | 120 | ALFF-to-GMV | NHE | 29 | -1.0459 | -1.17 | -0.92 | HC3 descriptive CI | Same-sample selected cluster; winner’s curse possible |
| ALFF-to-GMV | HE > HC | 1 | 120 | ALFF-to-GMV | HE | 30 | -0.7168 | -0.85 | -0.59 | HC3 descriptive CI | Same-sample selected cluster; winner’s curse possible |
| ALFF-to-GMV | HE > HC | 1 | 120 | ALFF | HC | 38 | -0.5637 | -0.59 | -0.53 | HC3 descriptive CI | Same-sample selected cluster; winner’s curse possible |
| ALFF-to-GMV | HE > HC | 1 | 120 | ALFF | NHE | 29 | -0.4148 | -0.46 | -0.37 | HC3 descriptive CI | Same-sample selected cluster; winner’s curse possible |
| ALFF-to-GMV | HE > HC | 1 | 120 | ALFF | HE | 30 | -0.3066 | -0.36 | -0.25 | HC3 descriptive CI | Same-sample selected cluster; winner’s curse possible |
| ALFF-to-GMV | HE > HC | 1 | 120 | GMV-A | HC | 38 | 0.4150 | 0.40 | 0.43 | HC3 descriptive CI | Same-sample selected cluster; winner’s curse possible |
| ALFF-to-GMV | HE > HC | 1 | 120 | GMV-A | NHE | 29 | 0.4182 | 0.40 | 0.44 | HC3 descriptive CI | Same-sample selected cluster; winner’s curse possible |
| ALFF-to-GMV | HE > HC | 1 | 120 | GMV-A | HE | 30 | 0.4446 | 0.43 | 0.46 | HC3 descriptive CI | Same-sample selected cluster; winner’s curse possible |
| ALFF-to-GMV | HE > HC | 1 | 120 | GMV-B | HC | 38 | 0.4224 | 0.41 | 0.43 | HC3 descriptive CI | Same-sample selected cluster; winner’s curse possible |
| ALFF-to-GMV | HE > HC | 1 | 120 | GMV-B | NHE | 29 | 0.4166 | 0.40 | 0.43 | HC3 descriptive CI | Same-sample selected cluster; winner’s curse possible |
| ALFF-to-GMV | HE > HC | 1 | 120 | GMV-B | HE | 30 | 0.4369 | 0.42 | 0.45 | HC3 descriptive CI | Same-sample selected cluster; winner’s curse possible |
| ALFF-to-GMV | HE > HC | 4 | 49 | ALFF-to-GMV | HC | 38 | -1.7578 | -1.86 | -1.65 | HC3 descriptive CI | Same-sample selected cluster; winner’s curse possible |
| ALFF-to-GMV | HE > HC | 4 | 49 | ALFF-to-GMV | NHE | 29 | -1.4298 | -1.56 | -1.30 | HC3 descriptive CI | Same-sample selected cluster; winner’s curse possible |
| ALFF-to-GMV | HE > HC | 4 | 49 | ALFF-to-GMV | HE | 30 | -1.2055 | -1.33 | -1.08 | HC3 descriptive CI | Same-sample selected cluster; winner’s curse possible |
| ALFF-to-GMV | HE > HC | 4 | 49 | ALFF | HC | 38 | -0.6682 | -0.70 | -0.63 | HC3 descriptive CI | Same-sample selected cluster; winner’s curse possible |
| ALFF-to-GMV | HE > HC | 4 | 49 | ALFF | NHE | 29 | -0.5473 | -0.59 | -0.50 | HC3 descriptive CI | Same-sample selected cluster; winner’s curse possible |
| ALFF-to-GMV | HE > HC | 4 | 49 | ALFF | HE | 30 | -0.4857 | -0.53 | -0.44 | HC3 descriptive CI | Same-sample selected cluster; winner’s curse possible |
| ALFF-to-GMV | HE > HC | 4 | 49 | GMV-A | HC | 38 | 0.4094 | 0.40 | 0.42 | HC3 descriptive CI | Same-sample selected cluster; winner’s curse possible |
| ALFF-to-GMV | HE > HC | 4 | 49 | GMV-A | NHE | 29 | 0.4165 | 0.40 | 0.43 | HC3 descriptive CI | Same-sample selected cluster; winner’s curse possible |
| ALFF-to-GMV | HE > HC | 4 | 49 | GMV-A | HE | 30 | 0.4389 | 0.42 | 0.46 | HC3 descriptive CI | Same-sample selected cluster; winner’s curse possible |
| ALFF-to-GMV | HE > HC | 4 | 49 | GMV-B | HC | 38 | 0.4169 | 0.41 | 0.43 | HC3 descriptive CI | Same-sample selected cluster; winner’s curse possible |
| ALFF-to-GMV | HE > HC | 4 | 49 | GMV-B | NHE | 29 | 0.4148 | 0.40 | 0.43 | HC3 descriptive CI | Same-sample selected cluster; winner’s curse possible |
| ALFF-to-GMV | HE > HC | 4 | 49 | GMV-B | HE | 30 | 0.4309 | 0.42 | 0.45 | HC3 descriptive CI | Same-sample selected cluster; winner’s curse possible |
| ALFF-to-GMV | Omnibus F test | 1 | 40 | ALFF-to-GMV | HC | 38 | -1.3956 | -1.49 | -1.30 | HC3 descriptive CI | Same-sample selected cluster; winner’s curse possible |
| ALFF-to-GMV | Omnibus F test | 1 | 40 | ALFF-to-GMV | NHE | 29 | -0.9390 | -1.08 | -0.80 | HC3 descriptive CI | Same-sample selected cluster; winner’s curse possible |
| ALFF-to-GMV | Omnibus F test | 1 | 40 | ALFF-to-GMV | HE | 30 | -0.5461 | -0.70 | -0.39 | HC3 descriptive CI | Same-sample selected cluster; winner’s curse possible |
| ALFF-to-GMV | Omnibus F test | 1 | 40 | ALFF | HC | 38 | -0.5494 | -0.59 | -0.51 | HC3 descriptive CI | Same-sample selected cluster; winner’s curse possible |
| ALFF-to-GMV | Omnibus F test | 1 | 40 | ALFF | NHE | 29 | -0.3741 | -0.43 | -0.32 | HC3 descriptive CI | Same-sample selected cluster; winner’s curse possible |
| ALFF-to-GMV | Omnibus F test | 1 | 40 | ALFF | HE | 30 | -0.2386 | -0.30 | -0.17 | HC3 descriptive CI | Same-sample selected cluster; winner’s curse possible |
| ALFF-to-GMV | Omnibus F test | 1 | 40 | GMV-A | HC | 38 | 0.4148 | 0.40 | 0.43 | HC3 descriptive CI | Same-sample selected cluster; winner’s curse possible |
| ALFF-to-GMV | Omnibus F test | 1 | 40 | GMV-A | NHE | 29 | 0.4166 | 0.40 | 0.43 | HC3 descriptive CI | Same-sample selected cluster; winner’s curse possible |
| ALFF-to-GMV | Omnibus F test | 1 | 40 | GMV-A | HE | 30 | 0.4432 | 0.43 | 0.46 | HC3 descriptive CI | Same-sample selected cluster; winner’s curse possible |
| ALFF-to-GMV | Omnibus F test | 1 | 40 | GMV-B | HC | 38 | 0.4223 | 0.41 | 0.43 | HC3 descriptive CI | Same-sample selected cluster; winner’s curse possible |
| ALFF-to-GMV | Omnibus F test | 1 | 40 | GMV-B | NHE | 29 | 0.4150 | 0.40 | 0.43 | HC3 descriptive CI | Same-sample selected cluster; winner’s curse possible |
| ALFF-to-GMV | Omnibus F test | 1 | 40 | GMV-B | HE | 30 | 0.4352 | 0.42 | 0.45 | HC3 descriptive CI | Same-sample selected cluster; winner’s curse possible |
| ALFF-to-GMV | Omnibus F test | 2 | 36 | ALFF-to-GMV | HC | 38 | 0.6888 | 0.43 | 0.95 | HC3 descriptive CI | Same-sample selected cluster; winner’s curse possible |
| ALFF-to-GMV | Omnibus F test | 2 | 36 | ALFF-to-GMV | NHE | 29 | -0.1209 | -0.32 | 0.08 | HC3 descriptive CI | Same-sample selected cluster; winner’s curse possible |
| ALFF-to-GMV | Omnibus F test | 2 | 36 | ALFF-to-GMV | HE | 30 | -0.5807 | -0.79 | -0.37 | HC3 descriptive CI | Same-sample selected cluster; winner’s curse possible |
| ALFF-to-GMV | Omnibus F test | 2 | 36 | ALFF | HC | 38 | 0.3214 | 0.21 | 0.43 | HC3 descriptive CI | Same-sample selected cluster; winner’s curse possible |
| ALFF-to-GMV | Omnibus F test | 2 | 36 | ALFF | NHE | 29 | -0.0344 | -0.11 | 0.05 | HC3 descriptive CI | Same-sample selected cluster; winner’s curse possible |
| ALFF-to-GMV | Omnibus F test | 2 | 36 | ALFF | HE | 30 | -0.2005 | -0.29 | -0.11 | HC3 descriptive CI | Same-sample selected cluster; winner’s curse possible |
| ALFF-to-GMV | Omnibus F test | 2 | 36 | GMV-A | HC | 38 | 0.4391 | 0.42 | 0.46 | HC3 descriptive CI | Same-sample selected cluster; winner’s curse possible |
| ALFF-to-GMV | Omnibus F test | 2 | 36 | GMV-A | NHE | 29 | 0.4229 | 0.39 | 0.45 | HC3 descriptive CI | Same-sample selected cluster; winner’s curse possible |
| ALFF-to-GMV | Omnibus F test | 2 | 36 | GMV-A | HE | 30 | 0.4380 | 0.41 | 0.46 | HC3 descriptive CI | Same-sample selected cluster; winner’s curse possible |
| ALFF-to-GMV | Omnibus F test | 2 | 36 | GMV-B | HC | 38 | 0.4476 | 0.43 | 0.47 | HC3 descriptive CI | Same-sample selected cluster; winner’s curse possible |
| ALFF-to-GMV | Omnibus F test | 2 | 36 | GMV-B | NHE | 29 | 0.4211 | 0.39 | 0.45 | HC3 descriptive CI | Same-sample selected cluster; winner’s curse possible |
| ALFF-to-GMV | Omnibus F test | 2 | 36 | GMV-B | HE | 30 | 0.4291 | 0.41 | 0.45 | HC3 descriptive CI | Same-sample selected cluster; winner’s curse possible |
| ALFF-to-GMV | Omnibus F test | 10 | 22 | ALFF-to-GMV | HC | 38 | 2.0819 | 1.42 | 2.75 | HC3 descriptive CI | Same-sample selected cluster; winner’s curse possible |
| ALFF-to-GMV | Omnibus F test | 10 | 22 | ALFF-to-GMV | NHE | 29 | 0.8874 | 0.38 | 1.39 | HC3 descriptive CI | Same-sample selected cluster; winner’s curse possible |
| ALFF-to-GMV | Omnibus F test | 10 | 22 | ALFF-to-GMV | HE | 30 | 0.0197 | -0.27 | 0.30 | HC3 descriptive CI | Same-sample selected cluster; winner’s curse possible |
| ALFF-to-GMV | Omnibus F test | 10 | 22 | ALFF | HC | 38 | 0.7385 | 0.51 | 0.97 | HC3 descriptive CI | Same-sample selected cluster; winner’s curse possible |
| ALFF-to-GMV | Omnibus F test | 10 | 22 | ALFF | NHE | 29 | 0.3165 | 0.13 | 0.51 | HC3 descriptive CI | Same-sample selected cluster; winner’s curse possible |
| ALFF-to-GMV | Omnibus F test | 10 | 22 | ALFF | HE | 30 | 0.0195 | -0.10 | 0.14 | HC3 descriptive CI | Same-sample selected cluster; winner’s curse possible |
| ALFF-to-GMV | Omnibus F test | 10 | 22 | GMV-A | HC | 38 | 0.3862 | 0.36 | 0.41 | HC3 descriptive CI | Same-sample selected cluster; winner’s curse possible |
| ALFF-to-GMV | Omnibus F test | 10 | 22 | GMV-A | NHE | 29 | 0.4153 | 0.38 | 0.45 | HC3 descriptive CI | Same-sample selected cluster; winner’s curse possible |
| ALFF-to-GMV | Omnibus F test | 10 | 22 | GMV-A | HE | 30 | 0.4449 | 0.42 | 0.47 | HC3 descriptive CI | Same-sample selected cluster; winner’s curse possible |
| ALFF-to-GMV | Omnibus F test | 10 | 22 | GMV-B | HC | 38 | 0.3964 | 0.37 | 0.42 | HC3 descriptive CI | Same-sample selected cluster; winner’s curse possible |
| ALFF-to-GMV | Omnibus F test | 10 | 22 | GMV-B | NHE | 29 | 0.4131 | 0.39 | 0.44 | HC3 descriptive CI | Same-sample selected cluster; winner’s curse possible |
| ALFF-to-GMV | Omnibus F test | 10 | 22 | GMV-B | HE | 30 | 0.4342 | 0.42 | 0.45 | HC3 descriptive CI | Same-sample selected cluster; winner’s curse possible |

Note.—Adjusted means and HC3 confidence intervals are descriptive because clusters were selected in the same sample; winner’s curse is possible. CI = confidence interval; GMV = gray matter volume; HC3 = heteroscedasticity-consistent covariance estimator type 3.

Supplementary Figure 1. Complete ReHo-to-GMV TIV-adjusted sensitivity results. Corrected TIV-adjusted maps are shown for the omnibus F test and directional contrasts. A sensitivity-only HC > NHE cluster is displayed separately. These maps summarize within-sample sensitivity to TIV adjustment and do not constitute independent validation. HC = healthy controls, HE = hepatic encephalopathy, NHE = no previous overt hepatic encephalopathy and West Haven grade 0 at enrollment, ReHo = regional homogeneity, TIV = total intracranial volume.

Supplementary Figure 2. Complete ALFF-to-GMV TIV-adjusted sensitivity results. Corrected TIV-adjusted maps are shown for the omnibus F test and directional contrasts. These maps summarize within-sample sensitivity to TIV adjustment and do not constitute independent validation. ALFF = amplitude of low-frequency fluctuation, HC = healthy controls, HE = hepatic encephalopathy, NHE = no previous overt hepatic encephalopathy and West Haven grade 0 at enrollment, TIV = total intracranial volume.

Supplementary Figure 3. All exploratory ROI-clinical association estimates. Points show covariate-adjusted partial Spearman correlations and lines show bootstrap 95% confidence intervals. No association survived within-family Benjamini–Hochberg FDR correction. Anatomical abbreviations shown in the panel correspond to the ROI identifiers and full anatomical labels listed in Supplementary Table 8. FDR = false discovery rate; ROI = region of interest.

# S2. Nonparametric Permutation-TFCE Sensitivity Analysis

The primary Gaussian random-field analyses remained primary. ReHo-to-GMV and ALFF-to-GMV were reassessed separately in PALM using the final 97 participants, a group cell-means design, and age, sex, education, and mean Jenkinson framewise displacement as covariates. Three pairwise contrasts were evaluated: C1, NHE minus HC; C2, HE minus HC; and C3, HE minus NHE. Inference used 10,000 permutations, the Freedman–Lane procedure, threshold-free cluster enhancement, and two-tailed testing. Corrected P-value images were thresholded at within-contrast TFCE-FWE P <0.05; joint correction across C1–C3 was an additional conservative analysis. Omnibus F tests were run separately without directional attribution.

All analyses used the strict 42,885-voxel mask and 18-connectivity for post-threshold spatial components. One-to-one cluster retention was audited only for the 19 primary GRF clusters with archived exact spatial masks; seven other primary clusters were not included in that audit.

Supplementary Table 18. PALM completion and quality control.

| **Run** | **Runtime, s** | **Status** | **Geometry** | **Finite** | **P-value range** |
| --- | --- | --- | --- | --- | --- |
| ReHo pairwise | 10811.984 | Completed | Pass | Pass | Pass |
| ReHo omnibus | 3713.823 | Completed | Pass | Pass | Pass |
| ALFF pairwise | 11692.886 | Completed | Pass | Pass | Pass |
| ALFF omnibus | 4074.039 | Completed | Pass | Pass | Pass |

Note.—PALM = Permutation Analysis of Linear Models.

Supplementary Table 19. Complete directional and omnibus TFCE-FWE results.

| **Metric** | **Test** | **Correction** | **Direction** | **Voxels** | **18-connected clusters** | **Peak statistic** | **Peak MNI** | **Threshold** |
| --- | --- | --- | --- | --- | --- | --- | --- | --- |
| ReHo-to-GMV | C1 | Within-contrast TFCE-FWE | Positive | 16 | 3 | 4.5785 | [27.0, -12.0, 72.0] | P < 0.05 |
| ReHo-to-GMV | C1 | Within-contrast TFCE-FWE | Negative | 56 | 3 | -4.5322 | [-27.0, -9.0, -6.0] | P < 0.05 |
| ReHo-to-GMV | C1 | Across-C1-C3 TFCE-FWE | Positive | 0 | 0 | NA | NA | P < 0.05 |
| ReHo-to-GMV | C1 | Across-C1-C3 TFCE-FWE | Negative | 0 | 0 | NA | NA | P < 0.05 |
| ReHo-to-GMV | C2 | Within-contrast TFCE-FWE | Positive | 43 | 2 | 5.9821 | [15.0, -18.0, 15.0] | P < 0.05 |
| ReHo-to-GMV | C2 | Within-contrast TFCE-FWE | Negative | 2376 | 9 | -5.8933 | [0.0, 51.0, 6.0] | P < 0.05 |
| ReHo-to-GMV | C2 | Across-C1-C3 TFCE-FWE | Positive | 10 | 1 | 5.9821 | [15.0, -18.0, 15.0] | P < 0.05 |
| ReHo-to-GMV | C2 | Across-C1-C3 TFCE-FWE | Negative | 1451 | 7 | -5.8933 | [0.0, 51.0, 6.0] | P < 0.05 |
| ReHo-to-GMV | C3 | Within-contrast TFCE-FWE | Positive | 0 | 0 | NA | NA | P < 0.05 |
| ReHo-to-GMV | C3 | Within-contrast TFCE-FWE | Negative | 0 | 0 | NA | NA | P < 0.05 |
| ReHo-to-GMV | C3 | Across-C1-C3 TFCE-FWE | Positive | 0 | 0 | NA | NA | P < 0.05 |
| ReHo-to-GMV | C3 | Across-C1-C3 TFCE-FWE | Negative | 0 | 0 | NA | NA | P < 0.05 |
| ReHo-to-GMV | Omnibus | Omnibus TFCE-FWE | Omnibus | 1015 | 13 | 19.6211 | [15.0, -18.0, 15.0] | P < 0.05 |
| ALFF-to-GMV | C1 | Within-contrast TFCE-FWE | Positive | 0 | 0 | NA | NA | P < 0.05 |
| ALFF-to-GMV | C1 | Within-contrast TFCE-FWE | Negative | 1406 | 13 | -4.7181 | [-12.0, -72.0, 45.0] | P < 0.05 |
| ALFF-to-GMV | C1 | Across-C1-C3 TFCE-FWE | Positive | 0 | 0 | NA | NA | P < 0.05 |
| ALFF-to-GMV | C1 | Across-C1-C3 TFCE-FWE | Negative | 381 | 16 | -4.7181 | [-12.0, -72.0, 45.0] | P < 0.05 |
| ALFF-to-GMV | C2 | Within-contrast TFCE-FWE | Positive | 1558 | 4 | 6.6689 | [-21.0, -42.0, -3.0] | P < 0.05 |
| ALFF-to-GMV | C2 | Within-contrast TFCE-FWE | Negative | 8280 | 3 | -5.5039 | [42.0, 42.0, 6.0] | P < 0.05 |
| ALFF-to-GMV | C2 | Across-C1-C3 TFCE-FWE | Positive | 820 | 16 | 6.6689 | [-21.0, -42.0, -3.0] | P < 0.05 |
| ALFF-to-GMV | C2 | Across-C1-C3 TFCE-FWE | Negative | 6614 | 11 | -5.5039 | [42.0, 42.0, 6.0] | P < 0.05 |
| ALFF-to-GMV | C3 | Within-contrast TFCE-FWE | Positive | 0 | 0 | NA | NA | P < 0.05 |
| ALFF-to-GMV | C3 | Within-contrast TFCE-FWE | Negative | 0 | 0 | NA | NA | P < 0.05 |
| ALFF-to-GMV | C3 | Across-C1-C3 TFCE-FWE | Positive | 0 | 0 | NA | NA | P < 0.05 |
| ALFF-to-GMV | C3 | Across-C1-C3 TFCE-FWE | Negative | 0 | 0 | NA | NA | P < 0.05 |
| ALFF-to-GMV | Omnibus | Omnibus TFCE-FWE | Omnibus | 3875 | 73 | 22.8579 | [-21.0, -42.0, -3.0] | P < 0.05 |

Note.—C1 = NHE − HC; C2 = HE − HC; C3 = HE − NHE. FWE = family-wise error; MNI = Montreal Neurological Institute; TFCE = threshold-free cluster enhancement.

Supplementary Table 20. Comparisons of the 19 archived exact GRF cluster masks with TFCE results using canonical primary cluster IDs.

| **Metric** | **Contrast** | **Cluster** | **Direction** | **GRF voxels** | **Within-contrast overlap** | **Retained** | **Dice** | **Jaccard** | **Within-contrast class** | **Joint-correction overlap** | **Joint-correction class** |
| --- | --- | --- | --- | --- | --- | --- | --- | --- | --- | --- | --- |
| ReHo-to-GMV | Omnibus | ReHo_Omnibus_C001 | Omnibus group effect | 31 | 1 | 3.2% | 0.0019 | 0.0010 | Partially retained | NA | Not applicable |
| ReHo-to-GMV | Omnibus | ReHo_Omnibus_C002 | Omnibus group effect | 40 | 40 | 100.0% | 0.0758 | 0.0394 | Fully retained | NA | Not applicable |
| ReHo-to-GMV | Omnibus | ReHo_Omnibus_C003 | Omnibus group effect | 35 | 35 | 100.0% | 0.0667 | 0.0345 | Fully retained | NA | Not applicable |
| ReHo-to-GMV | Omnibus | ReHo_Omnibus_C004 | Omnibus group effect | 77 | 77 | 100.0% | 0.1410 | 0.0759 | Fully retained | NA | Not applicable |
| ReHo-to-GMV | Omnibus | ReHo_Omnibus_C006 | Omnibus group effect | 38 | 38 | 100.0% | 0.0722 | 0.0374 | Fully retained | NA | Not applicable |
| ReHo-to-GMV | Omnibus | ReHo_Omnibus_C007 | Omnibus group effect | 75 | 75 | 100.0% | 0.1376 | 0.0739 | Fully retained | NA | Not applicable |
| ALFF-to-GMV | C2 | ALFF_HC>HE_C001 | HC > HE | 111 | 111 | 100.0% | 0.0265 | 0.0134 | Fully retained | 111 | Fully retained |
| ALFF-to-GMV | C2 | ALFF_HC>HE_C002 | HC > HE | 50 | 50 | 100.0% | 0.0120 | 0.0060 | Fully retained | 5 | Fully retained |
| ALFF-to-GMV | C2 | ALFF_HC>HE_C005 | HC > HE | 45 | 45 | 100.0% | 0.0108 | 0.0054 | Fully retained | 45 | Fully retained |
| ALFF-to-GMV | C2 | ALFF_HC>HE_C004 | HC > HE | 142 | 142 | 100.0% | 0.0337 | 0.0171 | Fully retained | 142 | Fully retained |
| ALFF-to-GMV | C2 | ALFF_HC>HE_C005 | HC > HE | 45 | 45 | 100.0% | 0.0108 | 0.0054 | Fully retained | 45 | Fully retained |
| ALFF-to-GMV | C2 | ALFF_HC>HE_C012 | HC > HE | 34 | 34 | 100.0% | 0.0082 | 0.0041 | Fully retained | 34 | Fully retained |
| ALFF-to-GMV | C2 | ALFF_HC>HE_C013 | HC > HE | 32 | 32 | 100.0% | 0.0077 | 0.0039 | Fully retained | 32 | Fully retained |
| ALFF-to-GMV | C2 | ALFF_HC>HE_C019 | HC > HE | 41 | 41 | 100.0% | 0.0099 | 0.0050 | Fully retained | 41 | Fully retained |
| ALFF-to-GMV | C2 | ALFF_HE>HC_C001 | HE > HC | 120 | 120 | 100.0% | 0.1430 | 0.0770 | Fully retained | 12 | Fully retained |
| ALFF-to-GMV | C2 | ALFF_HE>HC_C004 | HE > HC | 49 | 49 | 100.0% | 0.0610 | 0.0315 | Fully retained | 49 | Fully retained |
| ALFF-to-GMV | Omnibus | ALFF_Omnibus_C001 | Omnibus group effect | 40 | 40 | 100.0% | 0.0204 | 0.0103 | Fully retained | NA | Not applicable |
| ALFF-to-GMV | Omnibus | ALFF_Omnibus_C002 | Omnibus group effect | 36 | 36 | 100.0% | 0.0184 | 0.0093 | Fully retained | NA | Not applicable |
| ALFF-to-GMV | Omnibus | ALFF_Omnibus_C010 | Omnibus group effect | 22 | 22 | 100.0% | 0.0113 | 0.0057 | Fully retained | NA | Not applicable |

Note.—Only the 19 primary GRF clusters with archived exact masks were available for one-to-one comparison. Seven other primary clusters were not included in this audit. Cluster identifiers are canonical primary IDs from Supplementary Table 3; archived filenames were mapped to these IDs by metric, contrast, and extent. GRF = Gaussian random field; TFCE = threshold-free cluster enhancement.

Supplementary Table 21. Contrast-level TFCE findings relative to archived exact GRF masks.

| Metric | Test | Correction | Archived exact GRF-mask voxels | TFCE voxels | Overlap with archived exact masks | Archived clusters retained | Archived clusters not retained | TFCE voxels outside archived exact masks |
| --- | --- | --- | --- | --- | --- | --- | --- | --- |
| ReHo-to-GMV | C1 | Within-contrast TFCE-FWE | 0 | 72 | 0 | 0 | 0 | 72 |
| ReHo-to-GMV | C1 | Across-C1-C3 TFCE-FWE | 0 | 0 | 0 | 0 | 0 | 0 |
| ReHo-to-GMV | C2 | Within-contrast TFCE-FWE | 0 | 2419 | 0 | 0 | 0 | 2419 |
| ReHo-to-GMV | C2 | Across-C1-C3 TFCE-FWE | 0 | 1461 | 0 | 0 | 0 | 1461 |
| ReHo-to-GMV | C3 | Within-contrast TFCE-FWE | 0 | 0 | 0 | 0 | 0 | 0 |
| ReHo-to-GMV | C3 | Across-C1-C3 TFCE-FWE | 0 | 0 | 0 | 0 | 0 | 0 |
| ReHo-to-GMV | Omnibus | Omnibus TFCE-FWE | 296 | 1015 | 266 | 6 | 0 | 749 |
| ALFF-to-GMV | C1 | Within-contrast TFCE-FWE | 0 | 1406 | 0 | 0 | 0 | 1406 |
| ALFF-to-GMV | C1 | Across-C1-C3 TFCE-FWE | 0 | 381 | 0 | 0 | 0 | 381 |
| ALFF-to-GMV | C2 | Within-contrast TFCE-FWE | 669 | 9838 | 669 | 10 | 0 | 9169 |
| ALFF-to-GMV | C2 | Across-C1-C3 TFCE-FWE | 669 | 7434 | 669 | 10 | 0 | 6765 |
| ALFF-to-GMV | C3 | Within-contrast TFCE-FWE | 0 | 0 | 0 | 0 | 0 | 0 |
| ALFF-to-GMV | C3 | Across-C1-C3 TFCE-FWE | 0 | 0 | 0 | 0 | 0 | 0 |
| ALFF-to-GMV | Omnibus | Omnibus TFCE-FWE | 98 | 3875 | 98 | 3 | 0 | 3777 |

Note.—Columns referring to GRF masks are restricted to the exact primary masks available in the archive. A value of 0 archived exact-mask voxels does not indicate absence of a primary GRF effect. For example, the primary ReHo analysis contained 80 GRF voxels for C1 and 712 GRF voxels across the two C2 directions, but the corresponding exact directional masks were unavailable for one-to-one retention auditing. Accordingly, “TFCE voxels outside archived exact masks” should not be interpreted as TFCE-only voxels relative to the complete primary GRF result. TFCE = threshold-free cluster enhancement; GRF = Gaussian random field.

# S3. Voxel-wise local-GMV-adjusted functional sensitivity analysis

To address denominator sensitivity directly, separate voxel-wise PALM models were fitted for szReHo and zALFF in the final 97 participants. Group was the factor of interest, and age, sex, education, mean Jenkinson framewise displacement, and voxel-wise local GMV were entered as covariates. Local GMV was centered across participants at each voxel and entered as a voxel-wise explanatory variable with a common slope across groups. Three pairwise contrasts (NHE-HC, HE-HC, and HE-NHE) were evaluated using 500 permutations, Freedman-Lane nuisance handling, two-tailed threshold-free cluster enhancement (TFCE), PALM tail acceleration, and joint correction across the three pairwise contrasts within each metric. These models were intended as internal sensitivity analyses of denominator dependence rather than as replacements for the prespecified ratio analyses.

The strict common gray-matter mask contained 42,885 voxels. The ReHo model retained all 42,885 voxels. For ALFF, PALM excluded 365 voxels whose zALFF values were identically zero in all 97 participants, yielding an effective mask of 42,520 voxels. None of these exclusions was attributable to local-GMV values or nonfinite values. Direction was determined from the signed raw t-statistic at voxels surviving corrected two-tailed TFCE inference. Spatial correspondence with the original ratio results was summarized using same-direction voxel intersection, union, Dice coefficient, Jaccard index, and directional retention; opposite-direction intersections were examined as control comparisons.

After local-GMV adjustment, the dominant corrected findings remained concentrated in the HE-versus-HC contrast. ReHo showed 722 corrected HC>HE voxels and no corrected HE>NHE or NHE>HE effect. ALFF showed 1,683 HE>HC voxels and 6,221 HC>HE voxels for HE versus HC, while HE versus NHE remained null; a small NHE-versus-HC ALFF effect comprised four HC>NHE voxels. Same-direction spatial correspondence showed retention of 64.1% of the original ReHo-to-GMV HC>HE voxels (Dice=0.6063) and retention of 94.7% of the original ALFF-to-GMV HE>HC voxels and 100% of the original ALFF-to-GMV HC>HE voxels. The adjusted ALFF maps were substantially more extensive than the original ratio maps, resulting in lower symmetric Dice coefficients despite high original-map retention. Opposite-direction intersections were zero for both metrics. Detailed contrast-level, direction-specific cluster, and same-direction overlap results are reported in Supplementary Tables 22-25.

Supplementary Table 22. Local-GMV-adjusted PALM contrast-level results and effective-mask quality control.

| Metric | Contrast | Effective mask, voxels | Minimum corrected P | Significant voxels | Direction-specific voxels |
| --- | --- | --- | --- | --- | --- |
| ReHo | NHE - HC | 42,885 | 0.1160 | 0 | — |
| ReHo | HE - HC | 42,885 | 0.00171 | 722 | HC > HE: 722; HE > HC: 0 |
| ReHo | HE - NHE | 42,885 | 0.5300 | 0 | — |
| ALFF | NHE - HC | 42,520 | 0.03331 | 4 | HC > NHE: 4; NHE > HC: 0 |
| ALFF | HE - HC | 42,520 | 0.000408 | 7,904 | HE > HC: 1,683; HC > HE: 6,221 |
| ALFF | HE - NHE | 42,520 | 0.8760 | 0 | — |

Note.—Corrected P values are two-tailed TFCE family-wise error P values after joint correction across the three pairwise contrasts within each metric. The ALFF effective mask was smaller because 365 strict-mask voxels were identically zero across all 97 participants; no voxel was excluded because of nonfinite local-GMV values. ALFF = amplitude of low-frequency fluctuation; HC = healthy controls; HE = hepatic encephalopathy; NHE = no previous overt hepatic encephalopathy and West Haven grade 0 at enrollment; ReHo = regional homogeneity; TFCE = threshold-free cluster enhancement.

Supplementary Table 23. Direction-specific clusters from the local-GMV-adjusted ReHo HE-versus-HC contrast.

| Direction | Cluster ID | Extent, voxels | Minimum corrected P | Peak raw t | Peak MNI coordinates |
| --- | --- | --- | --- | --- | --- |
| HC > HE | 1 | 443 | 0.001710 | -5.026 | (45, 12, -6) |
| HC > HE | 2 | 142 | 0.009090 | -4.603 | (45, -9, 36) |
| HC > HE | 3 | 100 | 0.004490 | -5.367 | (0, 48, 6) |
| HC > HE | 4 | 17 | 0.02626 | -4.645 | (0, 6, 36) |
| HC > HE | 5 | 9 | 0.03904 | -4.309 | (-39, 0, -3) |
| HC > HE | 6 | 5 | 0.04560 | -3.577 | (27, 54, 9) |
| HC > HE | 7 | 4 | 0.04377 | -3.817 | (45, 15, 27) |
| HC > HE | 8 | 1 | 0.04850 | -3.954 | (51, -21, -12) |
| HC > HE | 9 | 1 | 0.04944 | -2.968 | (57, -12, 27) |

Note.—Clusters were defined after separating corrected HE-HC voxels by the sign of the raw t-statistic and applying 26-neighbor connectivity. Negative t values for the HE-HC contrast indicate HC > HE. MNI = Montreal Neurological Institute.

Supplementary Table 24. Direction-specific clusters from significant local-GMV-adjusted ALFF pairwise contrasts.

| Contrast | Direction | Cluster ID | Extent, voxels | Minimum corrected P | Peak raw t | Peak MNI coordinates |
| --- | --- | --- | --- | --- | --- | --- |
| NHE - HC | HC > NHE | 1 | 4 | 0.03331 | -4.736 | (-9, -75, 42) |
| HE - HC | HE > HC | 1 | 1664 | 0.000562 | 6.667 | (-18, -45, -6) |
| HE - HC | HE > HC | 2 | 18 | 0.03761 | 3.242 | (-3, -24, 9) |
| HE - HC | HE > HC | 3 | 1 | 0.04978 | 2.704 | (-18, 3, -15) |
| HE - HC | HC > HE | 1 | 6193 | 0.000408 | -5.769 | (45, 39, 3) |
| HE - HC | HC > HE | 2 | 17 | 0.03260 | -3.846 | (30, 21, 42) |
| HE - HC | HC > HE | 3 | 10 | 0.03904 | -3.477 | (21, -48, -12) |
| HE - HC | HC > HE | 4 | 1 | 0.04998 | -3.245 | (21, -54, -15) |

Note.—Direction-specific masks were created by combining corrected P ≤ 0.05 with the sign of the raw t-statistic before 26-neighbor connected-component analysis; opposite t-sign voxels were therefore not allowed to merge into the same directional cluster. The HE-NHE ALFF contrast was null after correction. ALFF = amplitude of low-frequency fluctuation; HC = healthy controls; HE = hepatic encephalopathy; NHE = no previous overt hepatic encephalopathy and West Haven grade 0 at enrollment; MNI = Montreal Neurological Institute.

Supplementary Table 25. Same-direction spatial correspondence between original functional-to-GMV ratio maps and local-GMV-adjusted functional maps.

| Metric / direction | Original ratio voxels | Local-GMV-adjusted voxels | Intersection | Union | Dice | Jaccard | Local-GMV overlap, % | Original retained, % | Opposite-direction intersection |
| --- | --- | --- | --- | --- | --- | --- | --- | --- | --- |
| ReHo HC > HE | 647 | 722 | 415 | 954 | 0.6063 | 0.4350 | 57.5 | 64.1 | 0 |
| ALFF HE > HC | 169 | 1,683 | 160 | 1,692 | 0.1728 | 0.0946 | 9.5 | 94.7 | 0 |
| ALFF HC > HE | 500 | 6,221 | 500 | 6,221 | 0.1488 | 0.0804 | 8.0 | 100.0 | 0 |

Note.—“Local-GMV overlap” is the percentage of the local-GMV-adjusted map overlapping the corresponding original ratio map; “Original retained” is the percentage of the original ratio map retained in the same direction after local-GMV adjustment. The low ALFF Dice coefficients reflect the substantially greater extent of the local-GMV-adjusted ALFF maps despite near-complete retention of the original ratio-defined voxels. Opposite-direction intersections compare each local-GMV-adjusted direction with the original ratio map in the reverse direction. ALFF = amplitude of low-frequency fluctuation; GMV = gray matter volume; ReHo = regional homogeneity.

# S4. Interpretive Boundaries

The primary ratio analyses remain the evidential focus. Component overlap, TIV adjustment, final-sample mask reconstruction, expanded-motion models, TFCE, and clinical associations are within-sample sensitivity or exploratory analyses. The ratios are operational mathematical measures and should not be interpreted as direct physiological coupling or as validated diagnostic, staging, predictive, biomarker, or causal measures. Supplementary Figure 4 provides a conceptual illustration of how similar functional-to-GMV ratio changes can arise from different numerator-denominator configurations, underscoring that these ratios are operational imaging measures rather than direct physiological measures of structure-function coupling. Local-GMV adjustment is likewise an internal sensitivity analysis and does not constitute independent validation. The exploratory within-HE current-versus-recovered analysis is likewise a sensitivity analysis and does not establish subgroup equivalence.

Supplementary Figure 4. Conceptual interpretation of functional-to-GMV ratios. Similar ratio changes can arise from different configurations of the functional numerator and GMV denominator. An increased ratio may reflect an increased functional numerator, a decreased GMV denominator, or both; when both numerator and denominator decrease, the ratio direction depends on their relative magnitudes. Therefore, functional-to-GMV ratios do not uniquely identify the underlying functional or structural contribution and should not be interpreted as direct physiological measures of structure-function coupling. Component analyses, local-GMV-adjusted models, and sensitivity analyses provide complementary interpretive safeguards.

# S5. Exploratory within-HE phenotype sensitivity analysis

To address heterogeneity within the operational HE group, an exploratory within-HE analysis compared participants with current West Haven grade 1–2 HE (n = 19) with participants who had previous OHE but had recovered to West Haven grade 0 at enrollment (n = 11). ReHo-to-GMV and ALFF-to-GMV were analyzed separately using PALM with age, sex, education, and mean Jenkinson framewise displacement as covariates. The original strict gray-matter mask was retained. Inference used 10,000 permutations, Freedman-Lane nuisance handling, two-tailed TFCE, and FWE correction at P < 0.050. The effective masks contained 42,885 voxels for ReHo-to-GMV and 42,520 voxels for ALFF-to-GMV.

No corrected subgroup difference was detected for either metric. The minimum corrected P values were 0.2231 for ReHo-to-GMV and 0.4599 for ALFF-to-GMV, with zero TFCE-FWE-corrected voxels in either direction for both metrics. In exploratory descriptive comparisons, the current WH1–2 subgroup showed lower PHES, MMSE, and MoCA scores than the recovered WH0 subgroup, whereas age, sex, education, mean FD, ammonia, Child-Pugh score, and MELD score did not differ significantly. These null imaging findings should not be interpreted as evidence of subgroup equivalence because the subgroup sizes were modest.

Supplementary Table 26. Exploratory current-versus-recovered HE subgroup characteristics and PALM results.

**A. Subgroup characteristics**

| **Variable** | **Recovered WH0 (n = 11)** | **Current WH1–2 (n = 19)** | **P value** |
| --- | --- | --- | --- |
| Age, years | 53.73 ± 6.47 | 58.58 ± 7.84 | 0.0797 |
| Male, n (%) | 8/11 (72.7%) | 13/19 (68.4%) | 1.0000 |
| Education, years | 8.91 ± 2.52 | 8.13 ± 2.54 | 0.4255 |
| Mean FD, mm | 0.1256 ± 0.0419 | 0.1125 ± 0.0406 | 0.4151 |
| PHES | -3.09 ± 2.07 | -6.05 ± 1.90 | 0.000946 |
| MMSE | 29.00 ± 0.77 | 26.05 ± 1.08 | 3.29 × 10^-9 |
| MoCA | 28.45 ± 1.13 | 24.11 ± 2.56 | 7.58 × 10^-7 |
| Blood ammonia, μmol/L | 84.99 ± 24.74 | 99.80 ± 44.45 | 0.2512 |
| Child-Pugh score | 9.00 ± 1.41 | 8.74 ± 2.02 | 0.6796 |
| MELD score | 13.32 ± 2.97 | 12.86 ± 4.88 | 0.7508 |

**B. PALM corrected results**

| **Metric** | **Effective mask, voxels** | **Minimum corrected P** | **Corrected voxels, P ≤ 0.05** |
| --- | --- | --- | --- |
| ReHo-to-GMV | 42,885 | 0.2231 | 0 |
| ALFF-to-GMV | 42,520 | 0.4599 | 0 |

Note.—Values are mean ± SD or n (%). Continuous-variable P values are from exploratory Welch t tests and the sex comparison used Fisher exact test; these descriptive P values were not adjusted for multiplicity. PALM models adjusted for age, sex, education, and mean FD and used 10,000 permutations with Freedman-Lane nuisance handling and two-tailed TFCE-FWE correction. No corrected subgroup difference was detected for either metric; this null finding is not evidence of equivalence. FD = framewise displacement; HE = hepatic encephalopathy; MELD = Model for End-Stage Liver Disease; MMSE = Mini-Mental State Examination; MoCA = Montreal Cognitive Assessment; OHE = overt hepatic encephalopathy; PHES = Psychometric Hepatic Encephalopathy Score; ReHo = regional homogeneity; TFCE = threshold-free cluster enhancement; WH = West Haven.
